# Supplementary material for: Estimated impact of the COVID-19 pandemic on cancer services and excess 1-year mortality in people with cancer and multimorbidity: near real-time data on cancer care, cancer deaths and a population-based cohort study
Source: BMJ Open. 2020 Nov 17;10(11):e043828. doi: 10.1136/bmjopen-2020-043828 (PMC7674020; doi:10.1136/bmjopen-2020-043828)
Supplement: Supplementary data [file bmjopen-2020-043828supp001.pdf]

Supplementary Methods

Description on 15 comorbidity clusters relevant to COVID-19

The PHE list included chronic respiratory disease, chronic heart disease, immunocompromised individuals, HIV, use of corticosteroids, obesity, diabetes, chronic kidney disease, chronic liver disease, chronic neurological disorders and splenic disorders. We have performed analyses for all the above conditions and have additionally considered hypertension, Crohn's disease, cystic fibrosis and rheumatoid arthritis. Given that condition clusters such as **(i)** chronic heart disease would involve a range of conditions, we have derived composite variables to include 15 conditions considered as cardiovascular disease (CVD) that included acute myocardial infarction, unstable angina, chronic stable angina, heart failure, cardiac arrest or sudden coronary death, transient ischemic attack, intracerebral haemorrhage, subarachnoid haemorrhage, ischemic stroke, abdominal aortic aneurysm, peripheral arterial disease, atrial fibrillation, congenital heart disease, hypertrophic and dilated cardiomyopathy and valve disease (multiple, mitral and aortic)[29]. We also considered **(ii)** Hypertension, defined as ≥140 mmHg systolic blood pressure (or ≥150 mmHg for people aged ≥60 years without diabetes and chronic kidney disease) and/or ≥90 mmHg diastolic blood pressure[30], **(iii)** type 2 diabetes, **(iv)** obesity, defined as a body mass index of ≥40kg/m^2, **(v)** chronic kidney disease (CKD), **(vi)** chronic obstructive pulmonary disease (COPD)[31], **(vii)** patients on immunosuppressive drugs (not cancer chemotherapy), **(viii)** patients with HIV or corticosteroid prescription, **(ix)** chronic neurological disorders, defined as a composite of Parkinson's disease, motor neuron disease, learning disability and cerebral palsy, **(x)** multiple sclerosis separately, **(xi)** splenic disorders, defined as a composite of splenomegaly, splenectomy and hyposplenism, **(xii)** chronic liver diseases, defined as a composite of chronic viral hepatitis B or C, primary biliary cholangitis, liver fibrosis, liver cirrhosis and non-alcoholic fatty liver disease, **(xiii)** Crohn's disease, **(xiv)** cystic fibrosis and **(xv)** rheumatoid arthritis[32].
