# Supplementary material for: Estimated impact of the COVID-19 pandemic on cancer services and excess 1-year mortality in people with cancer and multimorbidity: near real-time data on cancer care, cancer deaths and a population-based cohort study
Source: BMJ Open. 2020 Nov 17;10(11):e043828. doi: 10.1136/bmjopen-2020-043828 (PMC7674020; doi:10.1136/bmjopen-2020-043828)

Figure S1. Age-adjusted incidence rates for 24 primary cancers from England and the UK (International Agency for Research on Cancer [IARC]) For England data, cervix refers to both carcinoma in situ of cervix and primary malignancy of cervix. For IARC data, only cervix uteri are included. CRUK: Cancer Research UK.

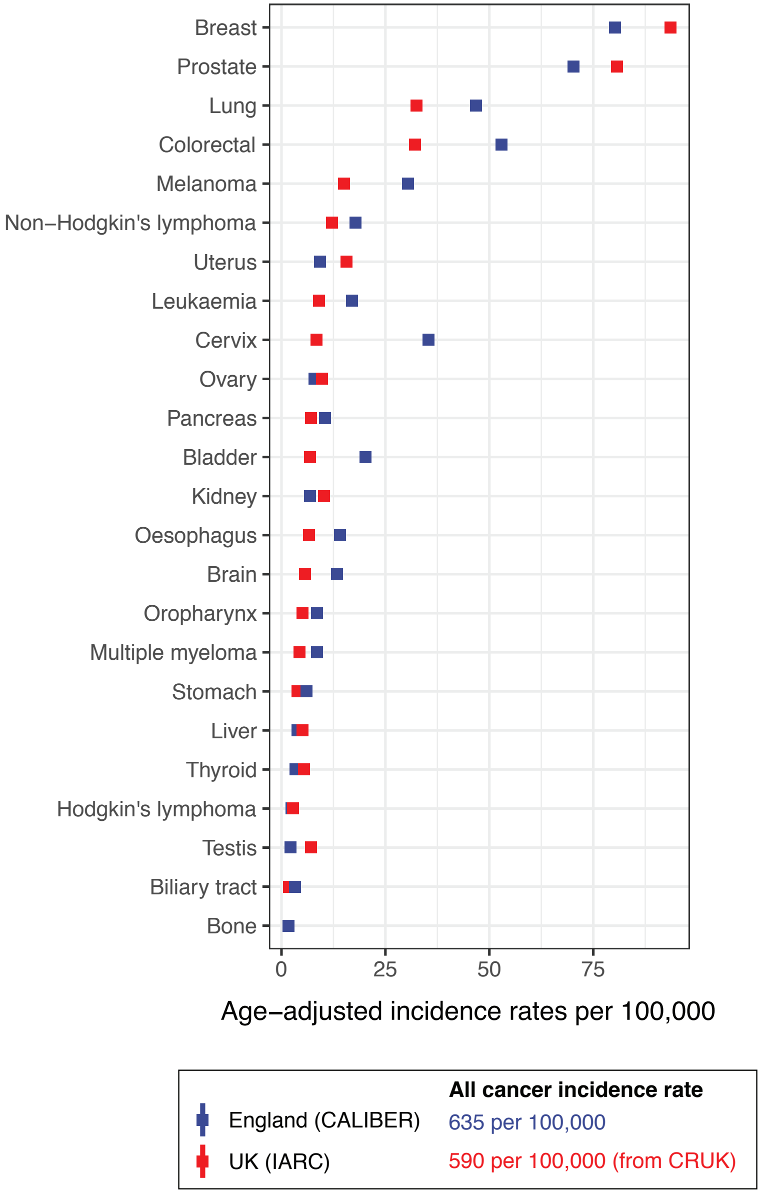

Figure S2. Total excess deaths for incident cancers over a 1-year period scaled up to the population of England aged 30+ consisting of 35 million individuals using England mortality estimates. We estimated direct excess deaths at a 10% infection rate. We estimated total (direct and indirect) excess deaths for 40% (10% infected, 30% affected) and 80% (10% infected, 70% affected) of the population.

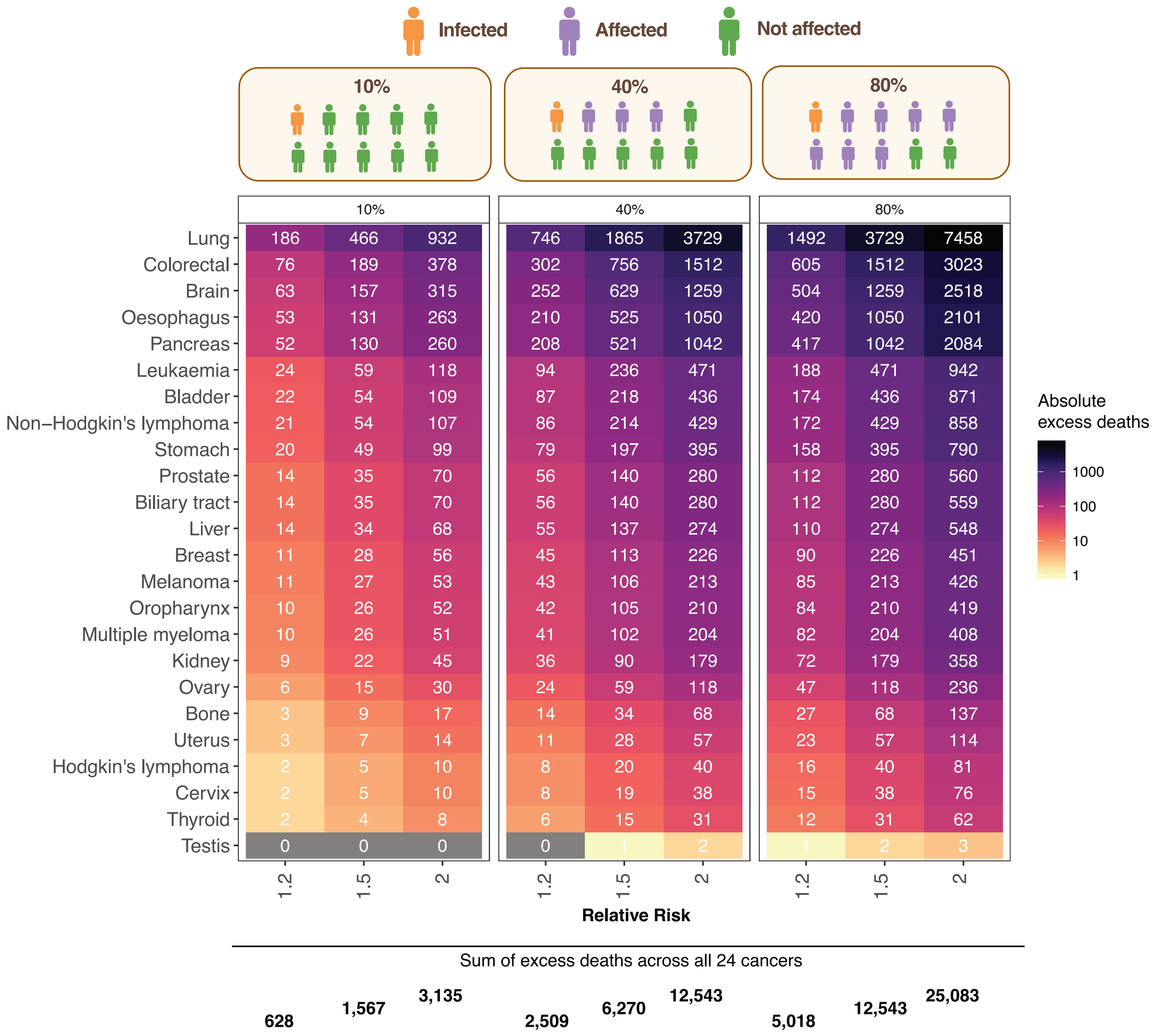

Figure S3. Total excess deaths for prevalent cancers over a 1-year period scaled up to the population of England aged 30+ consisting of 35 million individuals using England mortality estimates. We estimated direct excess deaths at a 10% infection rate. We estimated total (direct and indirect) excess deaths for 40% (10% infected, 30% affected) and 80% (10% infected, 70% affected) of the population.

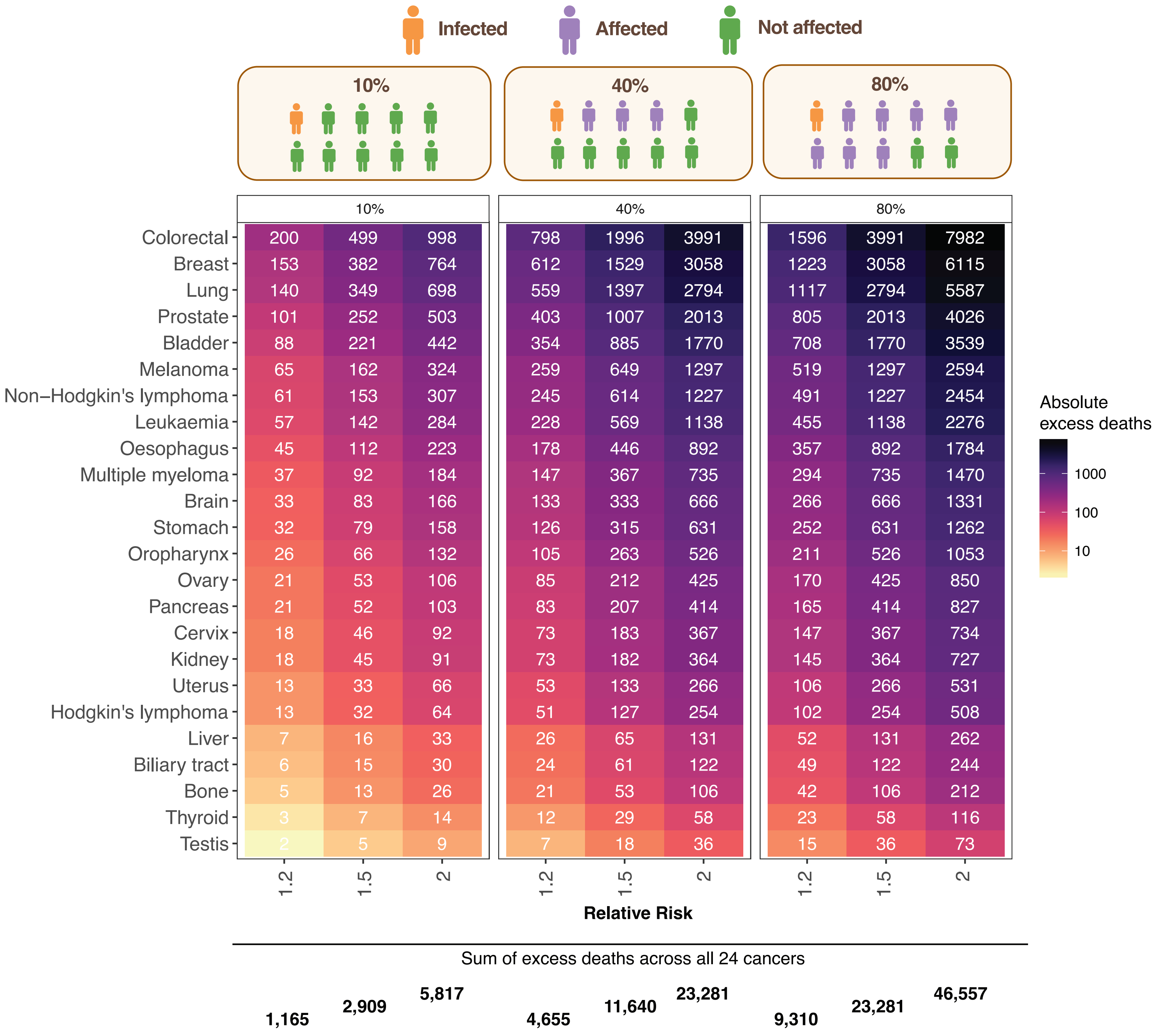

Figure S4. Proportion of patients with any of the 15 comorbidity clusters by cancer site for incident cancers (N=199,057) in a population of 3,862,012 adults in England. Age indicates mean age at diagnosis.

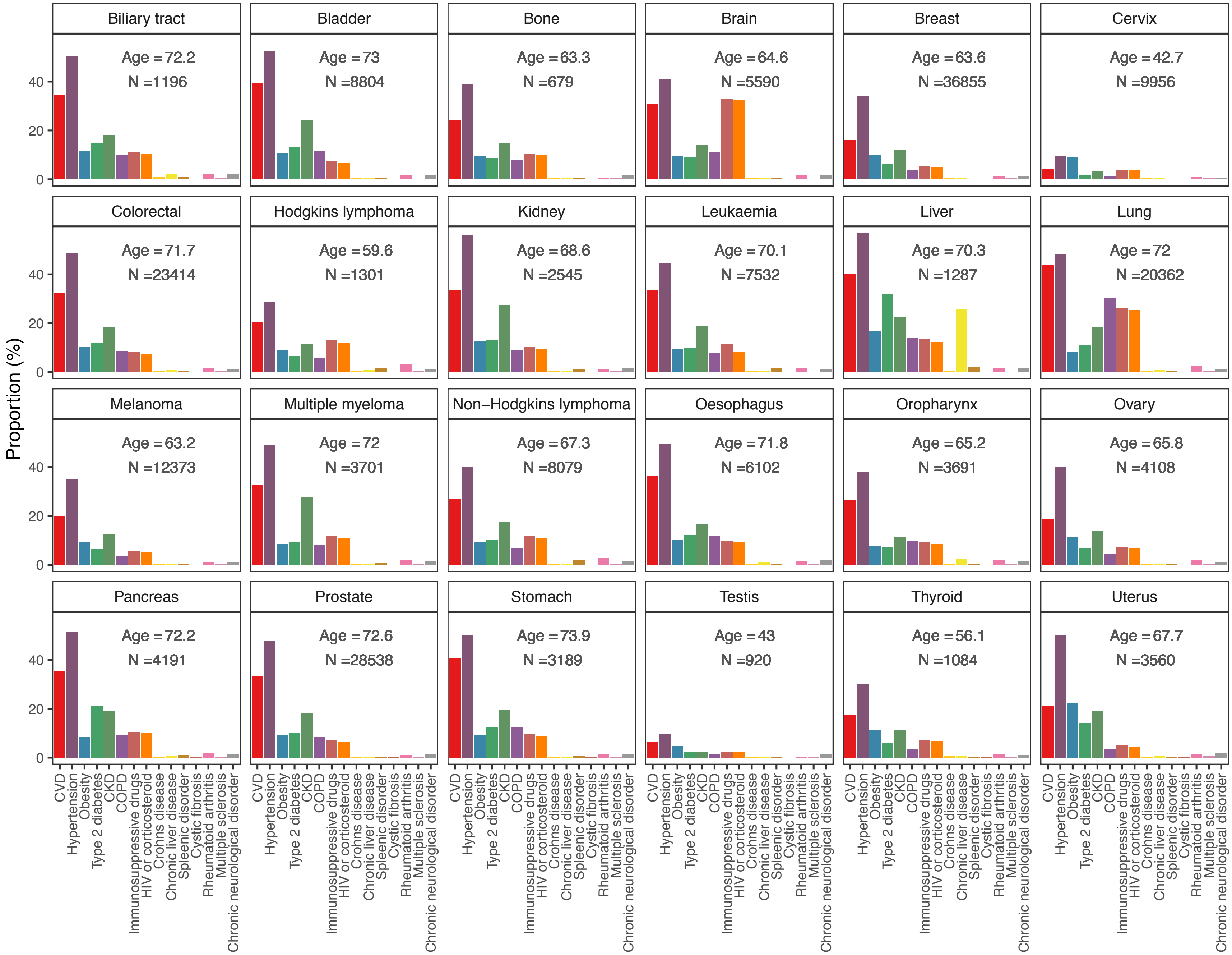

Figure S5. Proportion of patients with any of the 15 comorbidity clusters by cancer site for prevalent cancers (N=117,978) in a population of 3,862,012 adults in England. Age indicates mean age at diagnosis.

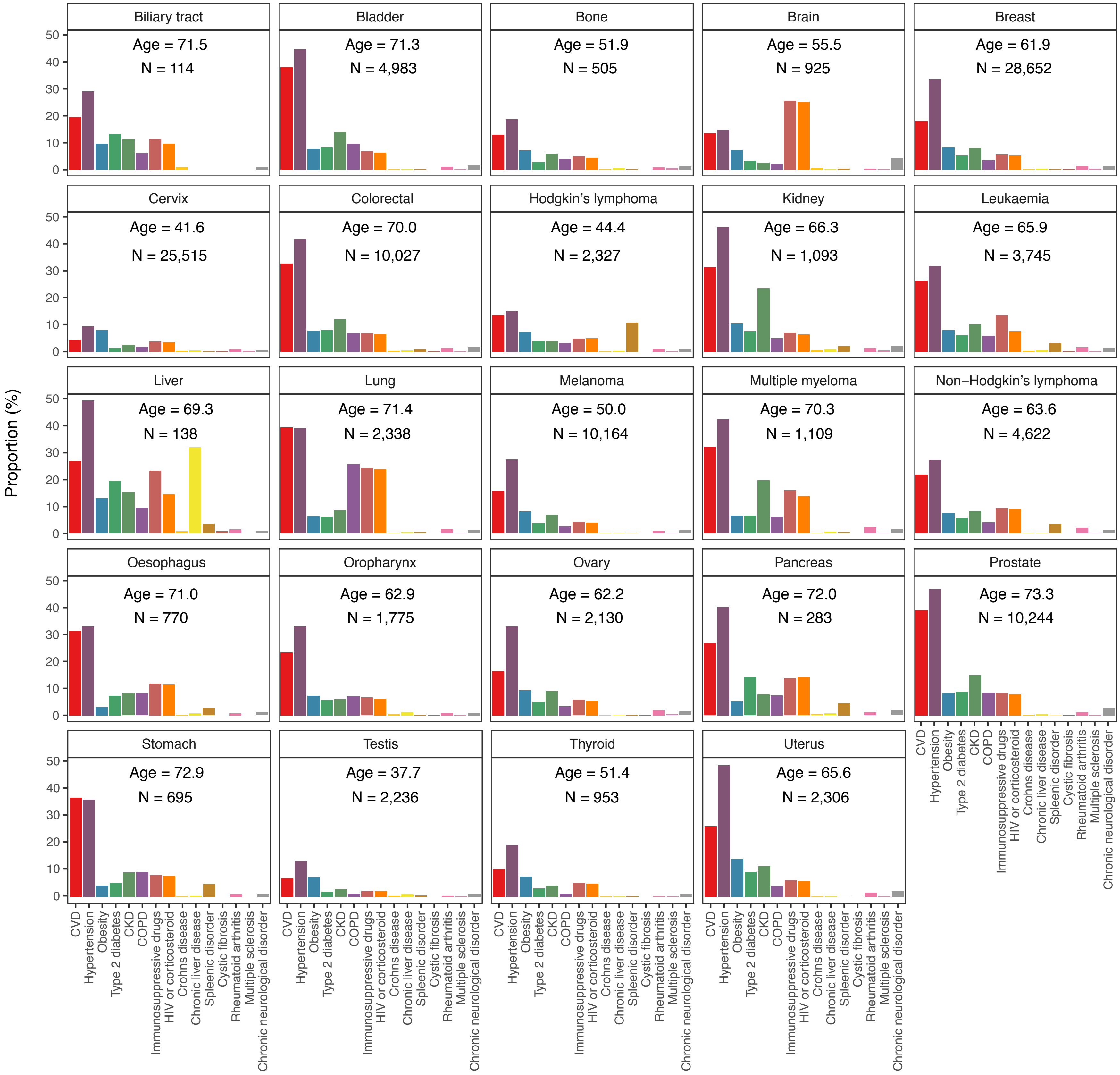

Figure S6. Forest plot of background (pre-COVID-19) 1-year cancer mortality for incident cases according to cancer site and number of underlying comorbidities in England. The whiskers are 95% confidence intervals.

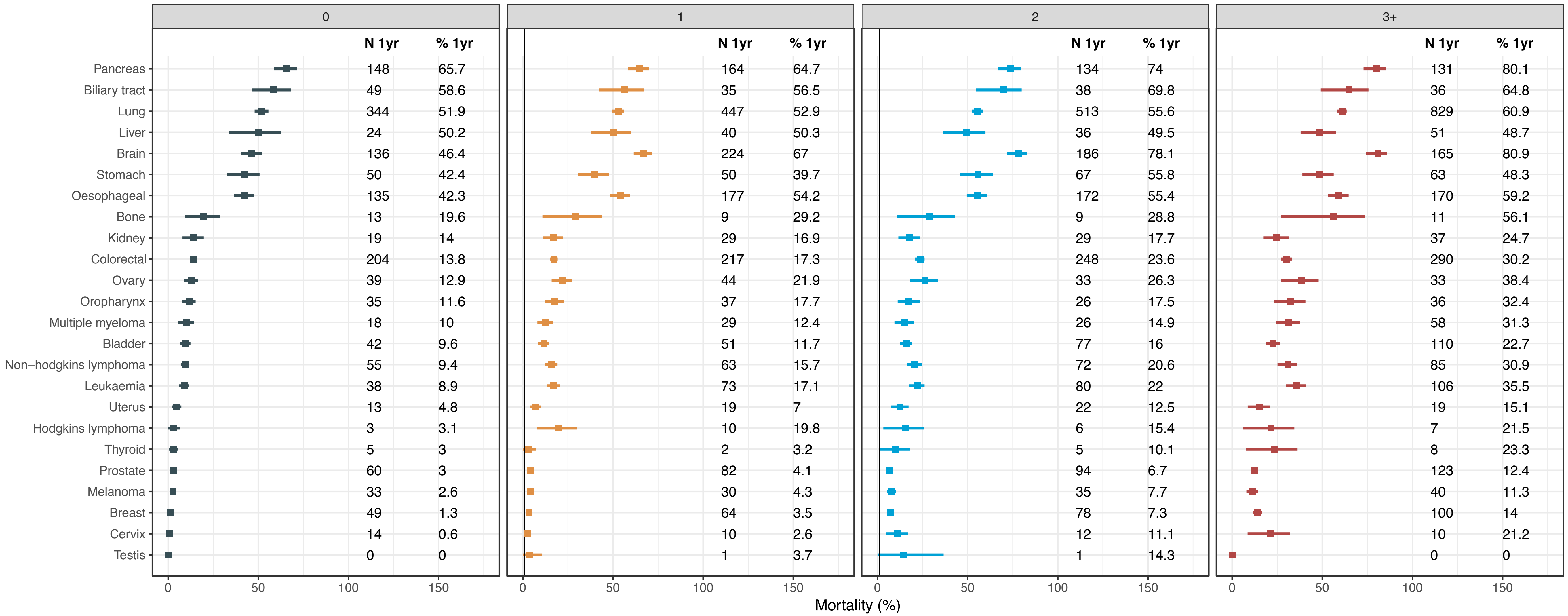

Figure S7. Forest plot of background (pre-COVID-19) 1-year cancer mortality for prevalent cases according to cancer site and number of underlying comorbidities in England. The whiskers are 95% confidence intervals.

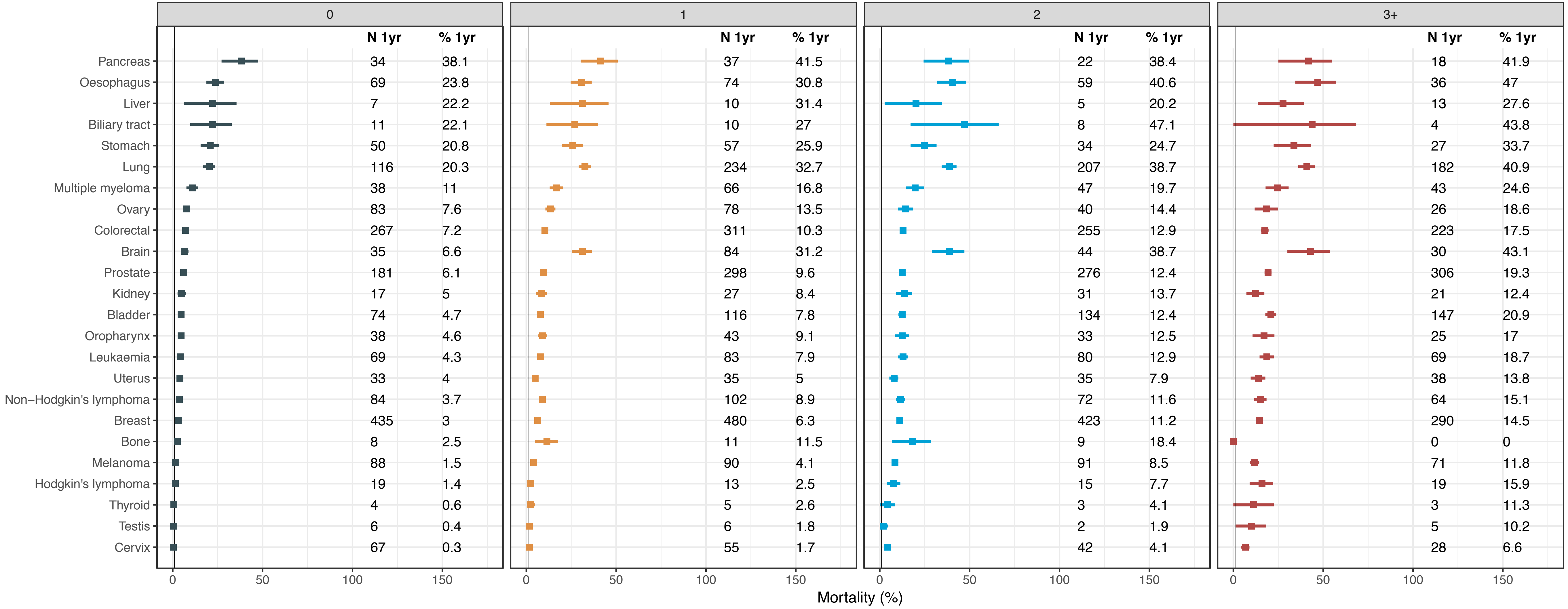

Figure S8. Total (direct and indirect) excess deaths for incident cancers by cancer site and number of comorbidities over a 1-year period. Stacked bar chart indicates the proportion of individuals with 0, 1, 2 and 3+ comorbidities by cancer site. We estimated total excess deaths for 40% (10% infected, 30% affected) of the population. Total excess deaths were scaled up to the population of England aged 30+ consisting of 35 million individuals using England mortality estimates for incident cancers.

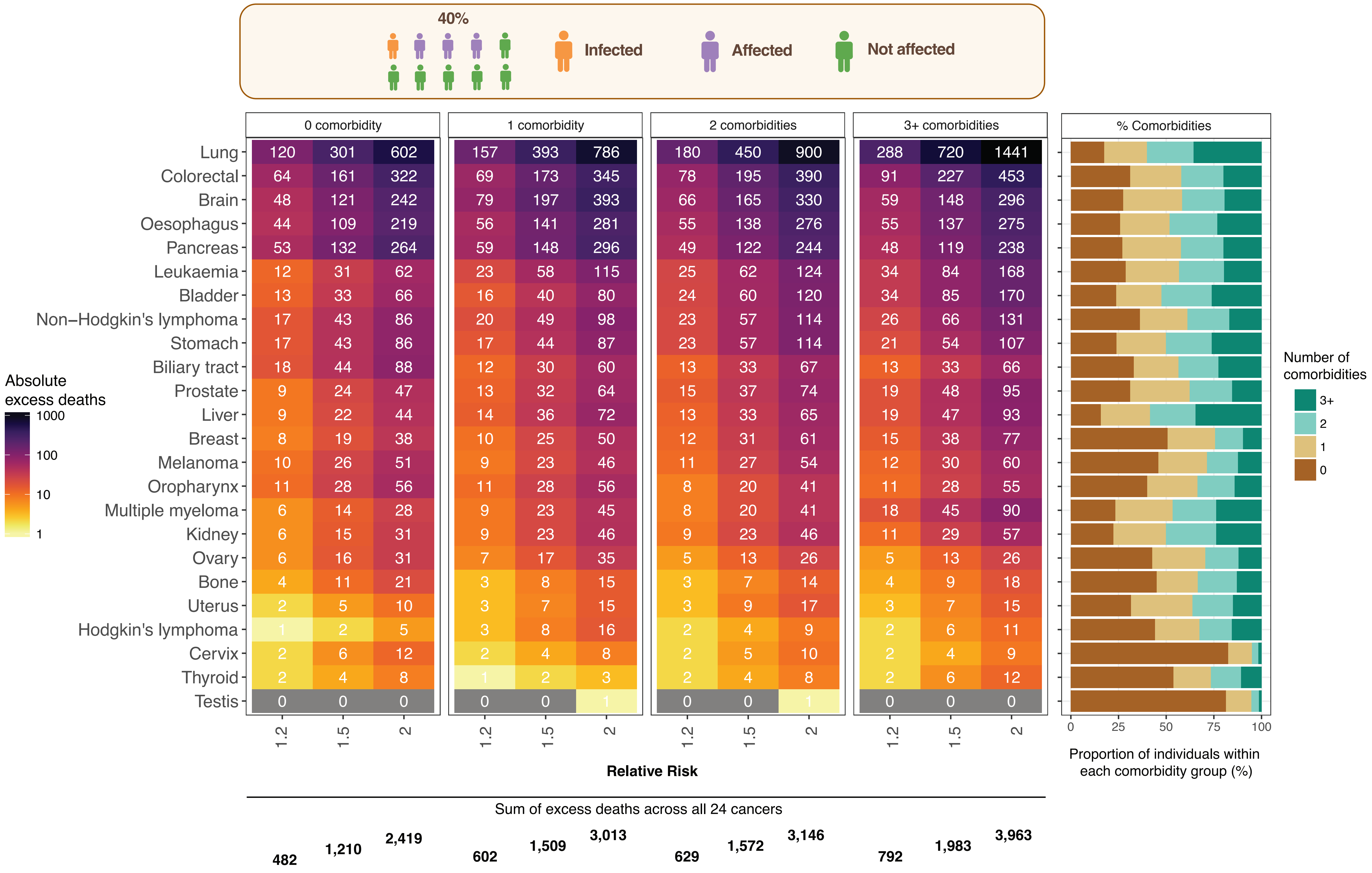

Figure S9. Total (direct and indirect) excess deaths for prevalent cancers by cancer site and number of comorbidities over a 1-year period. Stacked bar chart indicates the proportion of individuals with 0, 1, 2 and 3+ comorbidities by cancer site. We estimated total excess deaths for 40% (10% infected, 30% affected) of the population. Total excess deaths were scaled up to the population of England aged 30+ consisting of 35 million individuals using England mortality estimates for prevalent cancers.

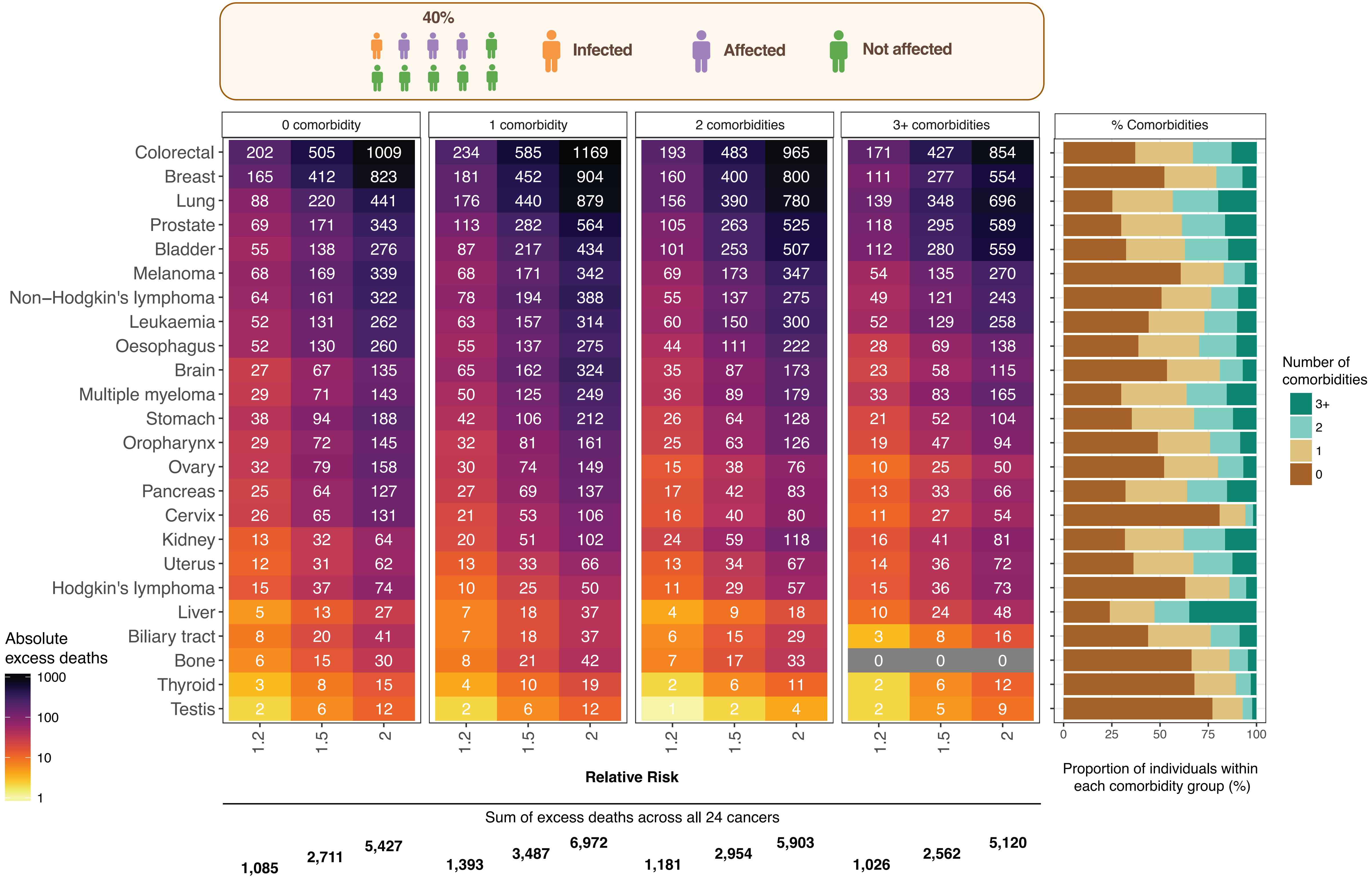

Supplement: Supplementary data [file bmjopen-2020-043828supp002.pdf]
