# Supplementary material for: Estimated impact of the COVID-19 pandemic on cancer services and excess 1-year mortality in people with cancer and multimorbidity: near real-time data on cancer care, cancer deaths and a population-based cohort study
Source: BMJ Open. 2020 Nov 17;10(11):e043828. doi: 10.1136/bmjopen-2020-043828 (PMC7674020; doi:10.1136/bmjopen-2020-043828)
Supplement: Author's manuscript [file bmjopen-2020-043828.draft_revisions.pdf]

BMJ Open is committed to open peer review. As part of this commitment we make the peer review history of every article we publish publicly available.

When an article is published we post the peer reviewers' comments and the authors' responses online. We also post the versions of the paper that were used during peer review. These are the versions that the peer review comments apply to.

The versions of the paper that follow are the versions that were submitted during the peer review process. They are not the versions of record or the final published versions. They should not be cited or distributed as the published version of this manuscript.

BMJ Open is an open access journal and the full, final, typeset and author-corrected version of record of the manuscript is available on our site with no access controls, subscription charges or pay-per-view fees (http://bmjopen.bmj.com).

If you have any questions on BMJ Open's open peer review process please email info.bmjopen@bmj.com

BMJ Open

Estimated impact of the Covid-19 pandemic on cancer services and excess 1-year mortality in people with cancer and multimorbidity: near-real-time data on cancer care, cancer deaths and a population-based cohort study

Journal:	*BMJ Open*
Manuscript ID	bmjopen-2020-043828
Article Type:	Original research
Date Submitted by the Author:	14-Aug-2020
Complete List of Authors:	Lai, Alvina; University College London, Institute of Health Informatics<br>Pasea, Laura; The Farr Institute of Health Informatics Research, University College ,<br>Banerjee, Amitava; University College London, Farr Institute of Health Informatics Research<br>Hall, Geoff; St James's University Teaching Hospital,<br>Denaxas, S; University College London, Institute of Health Informatics<br>Chang, Wai Hoong; University College London, Institute of Health Informatics<br>Katsoulis, M; University College London, Institute of Health Informatics<br>Williams, Bryan; University College London, Institute of Cardiovascular Science<br>Pillay, Deenan; University College London, Medicine<br>Noursadeghi, Mahdad; University College London, 4Division of Infection & Immunity<br>Linch, David; University College London<br>Hughes, Derralynn; Royal Free Hospital and University College Medical School, Lysosomal Storage Disorders Unit, Department of Academic Haematology<br>Forster, Martin; University College London, Institute of Health Informatics<br>Turnbull, Clare; Institute of Cancer Research<br>Fitzpatrick, Natalie; UCL, Epidemiology and Public Health<br>Boyd, Kathryn; Northern Ireland Cancer Network<br>Foster, Graham; Barts and The London School of Medicine,<br>Enver, Tariq; University College London, Cancer Institute<br>Nafilyan, Vahe; Office for National Statistics<br>Humberstone, Ben; Office for National Statistics<br>Neal, Richard; University of Leeds, Leeds Institute of Health Sciences<br>Cooper, Matt; UCL Partners<br>Jones, Monica; University of Leeds<br>Pritchard-Jones, Kathy; University College London, UCL Great Ormond Street Institute of Child Health; University College London Hospitals NHS Foundation Trust, North Central London Cancer Alliance<br>Sullivan, Richard; Kings Health Partners<br>Davie, Charlie; UCL Partners<br>Lawler, Mark; Queen's University belfast, CCRCB<br>Hemingway, Harry; UCL, Epidemiology and Public Health

Keywords:	COVID-19, ONCOLOGY, Health informatics < BIOTECHNOLOGY & BIOINFORMATICS

SCHOLARONE™ Manuscripts

BMJ

I, the Submitting Author has the right to grant and does grant on behalf of all authors of the Work (as defined in the below author licence), an exclusive licence and/or a non-exclusive licence for contributions from authors who are: i) UK Crown employees; ii) where BMJ has agreed a CC-BY licence shall apply, and/or iii) in accordance with the terms applicable for US Federal Government officers or employees acting as part of their official duties; on a worldwide, perpetual, irrevocable, royalty-free basis to BMJ Publishing Group Ltd ("BMJ") its licensees and where the relevant Journal is co-owned by BMJ to the co-owners of the Journal, to publish the Work in this journal and any other BMJ products and to exploit all rights, as set out in our licence.

The Submitting Author accepts and understands that any supply made under these terms is made by BMJ to the Submitting Author unless you are acting as an employee on behalf of your employer or a postgraduate student of an affiliated institution which is paying any applicable article publishing charge ("APC") for Open Access articles. Where the Submitting Author wishes to make the Work available on an Open Access basis (and intends to pay the relevant APC), the terms of reuse of such Open Access shall be governed by a Creative Commons licence – details of these licences and which Creative Commons licence will apply to this Work are set out in our licence referred to above.

Other than as permitted in any relevant BMJ Author's Self Archiving Policies, I confirm this Work has not been accepted for publication elsewhere, is not being considered for publication elsewhere and does not duplicate material already published. I confirm all authors consent to publication of this Work and authorise the granting of this licence.

For peer review only

Estimated impact of the Covid-19 pandemic on cancer services and excess 1-year mortality in people with cancer and multimorbidity: near-real-time data on cancer care, cancer deaths and a population-based cohort study

Alvina G. Lai§[1,2], Laura Pasea*[1,2], Amitava Banerjee*[1,2,3], Geoff Hall*[4,5,6], Spiros Denaxas[1,2,7,8], Wai Hoong Chang[1,2], Michail Katsoulis[1,2], Bryan Williams[7,9,10], Deenan Pillay[11], Mahdad Noursadeghi[11], David Linch[7,12], Derralynn Hughes[13,14], Martin D. Forster[9,13], Clare Turnbull[15], Natalie K. Fitzpatrick[1,2], Kathryn Boyd[16], Graham R. Foster[17], Tariq Enver[13], Vahe Nafilyan[18], Ben Humberstone[18], Richard D. Neal[19], Matt Cooper[4,5], Monica Jones[4,5], Kathy Pritchard-Jones[4,20,2122], Richard Sullivan[23], Charlie Davie[4,14,20], Mark Lawler§[4,24], Harry Hemingway§[1,2,6]

[1]Institute of Health Informatics, University College London, London, UK
[2]Health Data Research UK, University College London, London, UK
[3]Barts Health NHS Trust, The Royal London Hospital, Whitechapel Rd, London, UK
[4]DATA-CAN, Health Data Research UK hub for cancer hosted by UCLPartners, London, UK
[5]Leeds Institute of Medical Research, University of Leeds, Leeds, UK
[6]Leeds Teaching Hospitals NHS Trust, Leeds, UK
[7]University College London Hospitals NIHR Biomedical Research Centre, London, UK
[8]The Alan Turing Institute, London, UK
[9]University College London Hospitals NHS Trust, London, UK
[10]Institute of Cardiovascular Science, University College London, London, UK
[11]Division of Infection and Immunity, University College London, London, UK
[12]Department of Hematology, University College London Cancer Institute, London, UK
[13]University College London Cancer Institute, London, UK
[14]Royal Free NHS Foundation Trust, London, UK
[15]Division of Genetics and Epidemiology, Institute of Cancer Research, London, UK
[16]Northern Ireland Cancer Network, UK
[17]Barts Liver Centre, Blizard Institute, Queen Mary University of London, London, UK
[18]Office for National Statistics, London, UK
[19]Leeds Institute for Health Sciences, University of Leeds, Leeds, UK
[20]UCLPartners Academic Health Science Partnership, London UK
[21]Centre for Cancer Outcomes, University College London Hospitals NHS Foundation Trust, London, UK
[22]UCL Great Ormond Street Institute for Child Health, University College London, London, UK
[23]Conflict and Health Research Group, Institute of Cancer Policy, King's College London, London, UK
[24]Patrick G Johnston Centre for Cancer Research, Queen's University Belfast, UK

§ Joint senior authors
* Joint second authors

For correspondence: Alvina G. Lai (alvina.lai@ucl.ac.uk)

For peer review only - http://bmjopen.bmj.com/site/about/guidelines.xhtml

Abstract:

Objectives: To estimate the impact of the covid-19 pandemic on cancer care services and overall (direct and indirect) excess deaths in people with cancer.

Methods: We employed near real-time weekly data on cancer care to determine the adverse effect of the pandemic on cancer services. We also used these data, together with national death registrations until June 2020 to model deaths, in excess of background (pre-covid-19) mortality, in people with cancer. Background mortality risks for 24 cancers with and without covid-19-relevant comorbidities were obtained from population-based primary care cohort (Clinical Practice Research Datalink) on 3,862,012 adults in England.

Results: Declines in urgent referrals (median = -70.4%) and chemotherapy attendances (median = -41.5%) to a nadir (lowest point) in the pandemic were observed. By 31st May, these declines have only partially recovered; urgent referrals (median = -44.5%) and chemotherapy attendances (median = -31.2%). There were short-term excess death registrations for cancer (without covid-19), with peak relative risk (RR) of 1.17 at week ending 3rd April. The peak RR for all-cause deaths was 2.1 from week ending 17th April. Based on these findings and recent literature, we modelled 40% and 80% of cancer patients being affected long-term by the pandemic. At 40% affected, we estimated 1-year total (direct and indirect) excess deaths in people with cancer as between 7,165 and 17,910, using RR of 1.2 and 1.5 respectively, where 78% of excess deaths occur in patients with ≥ 1 comorbidity.

Conclusions: Dramatic reductions were detected in the demand for, and supply of, cancer services which have not fully recovered with lockdown easing. These may contribute, over a 1-year time horizon, to substantial excess mortality among people with cancer and multimorbidity. It is urgent to understand how the recovery of general practitioner, oncology and other hospital services might best mitigate these long-term excess mortality risks.

For peer review only - http://bmjopen.bmj.com/site/about/guidelines.xhtml

For peer review only

Strengths and limitations of this study

- This is the first study that used hospital data and a predictive model to dissect and quantify the adverse impact on mortality of the pandemic on patients with cancer and multimorbidity.
- This study used the breadth of longitudinal information in primary care records from the Clinical Practice Research Datalink to generate background (pre-COVID-19) mortality estimates for patients with cancer.
- This study generated 1-year mortality estimates for 24 cancer types and evaluated the extent by which multimorbidity influences mortality risk in patients with cancer. We considered 15 comorbidity clusters, which include 40 non-malignant comorbidities defined by the Public Health England as associated with severe and fatal covid-19 infection.
- This study modelled excess deaths using information on background mortality risk and plausible relative risk estimates obtained from the Office for National Statistics and other published studies.
- A limitation of this study is the use of primary care health records which may have missed cases of cancer resulting in more conservative estimations of excess deaths.

For peer review only - http://bmjopen.bmj.com/site/about/guidelines.xhtml

Introduction:

The covid-19 pandemic may cause additional (excess) deaths due both to the direct effects of infection and the indirect effects that result from the repurposing of health services designed to address the pandemic[1]. People with cancer are at increased risk of contracting and dying from SARS-CoV-2 infection[2,3]. Optimal cancer care must balance protecting patients from SARS-CoV-2 infection, with the need for continued access to early diagnosis and delivery of optimal treatment[4,5]. Professional cancer associations internationally have recommended reducing systemic anti-cancer treatment, surgery and risk-adapted radiotherapy[6]. In June 2020, the NHS released statistics for April 2020, indicating that referrals to a consultant for urgent diagnosis of cancer had fallen by 60%[7]. Some cancer surgical procedures have been postponed and cancer screening programmes paused[8–13].

However, covid-19-induced healthcare service reconfiguration and recovery have, to date, not been informed by near real-time hospital data quantifying the extent of disruption for cancer patients resulting from this service reconfiguration, nor its impact on excess deaths in people with cancer. A previous study has employed literature-based estimates to model the impact of potential diagnostic delays in colorectal cancer during the covid-19 pandemic[14,15]. Short-term (30 days) death in people with cancer and covid-19 is importantly driven by (treatable) comorbidities such as hypertension and cardiovascular disease[16]. Public Health England (PHE) have identified patients with these and a wide range of other non-malignant conditions as at greater risk of developing severe illness from SARS-CoV-2 exposure[17,18], while multimorbidity in cancer is an increasing clinical concern[19,20]. For general practitioners and oncologists, evidence is required on the pan-cancer estimation of mortality risks according to type and number of comorbid conditions. Such evidence may inform individual decisions about physical isolation and shielding, as well as the need to ensure that patients access specialist cancer care and seek preventive care for non-malignant comorbidities.

Our objectives were: (i) to quantify changes in cancer care, reporting near-real-time weekly data (to June 2020) for urgent referral (for early diagnosis of cancer) and chemotherapy attendance (for treatment of cancer); (ii) to quantify short-term direct and indirect excess deaths using near real-time weekly death registrations from the Office for National Statistics (ONS); (iii) to estimate the number of annual direct (covid-19) and indirect excess deaths using population-based 1-year Kaplan-Meier mortality estimates for 24 cancer types and (iv) to determine the extent by which multimorbidity contributes to these excess deaths.

For peer review only

For peer review only - http://bmjopen.bmj.com/site/about/guidelines.xhtml

Methods:

Weekly near real-time hospital data

To estimate the extent to which changes in cancer services during different phases of the pandemic (pre-lockdown, lockdown, post lockdown easing) have impacted on cancer care delivery, we sought weekly information for urgent cancer referrals for early diagnosis ('two-week-wait' [2WW]), an indicator of both patient demand and health service supply i.e., how well the service is ensuring that individuals with suspicious symptoms are rapidly prioritised to the diagnostic cancer pathway) and chemotherapy attendances (an indicator of supply and a proxy for possible adverse effects of the pandemic on the cancer treatment pathway). We employed the UK's Health Data Research Hub for Cancer (DATA-CAN) [21] to approach eight hospital trusts (in Leeds, London and Northern Ireland) and sought data from January 2019 to June 2020 to control for seasonal changes. Each hospital trust rapidly provided the requested data and permission to share these data in the public domain. We estimated the % change in weekly activity compared to the mean activity in 2019.

Weekly near real-time death registration data

To estimate direct (among those infected) and indirect impact of the covid-19 pandemic on deaths, we sought weekly counts of deaths in England and Wales from the Office for National Statistics (ONS), with causes classified by the ONS as covid-19 deaths, non-covid-19 deaths excluding cancer and cancer deaths.

Study population: primary care population-based cohort

To estimate pre-covid-19 incidence and mortality in individuals with cancer, we used population-based electronic health records in England from primary care data from the Clinical Practice Research Datalink (CPRD) linked to the ONS death registration. We used this primary care data source because of the extensive information on comorbidities (which may be lacking in cancer registry data). The study population was 3,862,012 adults aged ≥ 30 years, registered with a general practice from 1 January 1997 to 1 January 2017, with at least 1 year of follow-up data. CPRD data are representative of the English population in terms of age, sex, mortality and ethnicity[22–24], with extensive evidence of validity[25]. This study was performed as part of the CALIBER programme (https://www.ucl.ac.uk/health-informatics/caliber). CALIBER is an open-access research resource consisting of information, tools and phenotyping algorithms available through the CALIBER Portal (https://caliberresearch.org/portal)[26,27]. The study was approved by the MHRA (UK) Independent Scientific Advisory Committee (20_074R2), under Section 251 (NHS Social Care Act 2006).

Open-access definitions of disease using electronic health records

We defined non-fatal incident cases (as alive for at least 30 days following cancer diagnosis) and prevalent cases of cancer across 24 primary cancer sites according to previously validated CALIBER

For peer review only - http://bmjopen.bmj.com/site/about/guidelines.xhtml

electronic health record phenotypes. Incident cancers were defined as new cancer diagnoses after the study entry into CPRD (baseline). Prevalent cancers were defined as cancer diagnoses recorded at any time prior to baseline. The cancers included: biliary tract, bladder, bone, brain, breast, cervix, colorectal, Hodgkin's lymphoma, kidney, leukaemia, liver, lung, melanoma, multiple myeloma, non-Hodgkin's lymphoma, oesophagus, oropharynx, ovary, pancreas, prostate, stomach, testis, thyroid and uterus[28]. Phenotype definitions of cancers and covid-19-relevant comorbidities are available at https://caliberresearch.org/portal and have previously been validated[29–32]. Phenotypes were generated from hospital and primary care information recorded in primary care, using Read clinical terminology (version 2).

Comorbidities relevant to covid-19

We examined 15 comorbidity clusters, which include 40 non-malignant comorbidities defined by PHE as associated with severe and fatal covid-19 infection[17,18]. We separately estimated the proportion of patients with each comorbidity at study entry (prevalent cancers), and at the date of the first diagnosis of incident cancer. The PHE list included chronic respiratory disease, chronic heart disease, immunocompromised individuals, HIV, use of corticosteroids, obesity, diabetes, chronic kidney disease, chronic liver disease, chronic neurological disorders and splenic disorders. A full list of the conditions we examined, and their definitions is provided in Supplementary Methods.

Estimating incidence rates and 1-year mortality

We estimated incidence rates per 100,000 person-years and 1-year mortality in our study population. Estimated incidence rates for the number of new cancers by cancer site were compared with those for the UK from the International Agency for Research on Cancer (IARC) and were found to be representative. We estimated baseline 1-year mortality risk following cancer diagnosis for both incident and prevalent cancers using Kaplan-Meier analyses stratified by cancer sites and number of (non-cancer) comorbid conditions (0, 1, 2 and 3+). We used the most recent 5 years of data (2012-2016) to estimate 1-year mortality.

Estimating 1-year direct excess deaths

Excess deaths were estimated by applying relative risks (RRs) to the background 1-year mortality risk. Direct excess deaths (due to or with covid-19) were modelled using the range of relative risks (1.2, 1.5 and 2.0) previously reported in studies of cancer and covid-19 deaths.[3,33] We applied these RRs to 10% of the population (the directly "infected"), based on recent SARS CoV-2 seroprevalence estimates in the UK[34,35] and other countries[36,37]. Although the infection rate will change depending on the phase of the pandemic, we assumed an infection rate over 1 year in line with the first wave of the pandemic.

For peer review only - http://bmjopen.bmj.com/site/about/guidelines.xhtml

Estimating 1-year total (direct and indirect) excess deaths

Indirect excess deaths (due to pandemic-induced health service reconfiguration) were estimated by applying RRs for excess cancer deaths observed using ONS data, by taking the number of weekly cancer deaths from January 2020 divided by the weekly average over the last 5 years. We assumed that the effects of service change may not translate to an immediate increase in excess deaths. We have applied the RR of 1.2, 1.5 and 2 to 40% (10% infected, 30% affected) and 80% (10% infected, 70% affected) of the population and modelled excess deaths over a 12-month period to capture medium term effects. We chose this range of indirectly “affected” population based on our real-time estimates of the degree of perturbation in cancer care during the pandemic and patient reports that clinical care had been cancelled during the pandemic for 53%-70% of patients with cancer or other conditions[38].

To project the study estimates of excess deaths to the whole English population, we employed the 2018 population estimate, where the number of deaths is scaled up to a population of 35,407,313 individuals aged 30 and above[39]. All analyses were performed using R (version 3.4.3).

For peer review only

For peer review only - http://bmjopen.bmj.com/site/about/guidelines.xhtml

Results

Near real-time data on cancer care

Evaluating data from 291,792 people with suspected cancer and 150,636 patients with cancer attending for chemotherapy from January 2019 to June 2020, we initially characterised the pre-pandemic basal level of activity (2019 average), including seasonal variations (Figure 1). Using the date of the 50th patient diagnosed with covid-19 as the starting point of the pandemic, we observed that urgent referrals fell by 70.4% (range: -68.7% to -84.3%), while chemotherapy attendances declined by 41.5% (range: -26.3% to -63.4%) (Figure 1). To highlight these adverse impacts, we provided these data to Chief Medical Officers in all 4 nations of the United Kingdom and the National Director for Cancer (England). We have also continued to provide regular updates of this intelligence to the Scientific Advisory Group for Emergencies. Since the NHS letter on 29 April 2020 re-starting cancer and other services[40], and since easing of lockdown (11 May 2020), there has been evidence of recovery for the urgent two-week-wait referrals (-55.4% to -40.0%; median = -44.5%), and chemotherapy attendances (-37.1% to 3.9%; median = -31.2%) (Figure 1).

Near real-time data on cancer, covid-19 and other deaths

We found an excess in cancer deaths with a peak in the week ending 3rd April 2020 with a relative risk (RR) of 1.17 (Figure 2B). There were 1,307 excess cancer deaths from 13th March to 15th May 2020 compared to the 5-year average based on weekly registration of deaths for England and Wales (Figure 2A). There were 41,105 covid-19 deaths up until 15th May 2020. For non-covid-19 deaths (excluding cancers), we found that the peak occurred with a RR of 1.37 on 24th April 2020. The peak RR for all-cause deaths was 2.1, from week of 17th April 2020.

Estimations on direct excess deaths by cancer site over 1-year

We estimated direct excess covid-19 deaths based on a SARS CoV-2 infection rate of 10% and background 1-year mortality risks (Figure 3A). For both incident and prevalent cancers combined, we estimated 1,790, 4,479 and 8,957 direct excess deaths at RR of 1.2, 1.5 and 2.0 respectively (Figure 3B). Figures S2 and S3 show the separate direct excess death estimates for incident and prevalent cancers. Incidence rates for 24 cancer types were shown in Figure S1.

Estimations of total (direct and indirect) excess deaths by cancer site over a 1-year

When applying RRs of 1.2 or 1.5 to 40% (10% infected, 30% affected) of the population of people with cancer (both incident and prevalent cancers), we estimated 7,165 and 17,910 total excess deaths respectively (Figure 3B). When applying these RRs to 80% (10% infected, 70% affected) of the population of people with cancer, we estimated 14,326 and 35,817 total excess deaths respectively (Figure 3B). Figures S2 and S3 show the separate total excess death estimates for incident and prevalent cancers.

For peer review only - http://bmjopen.bmj.com/site/about/guidelines.xhtml

Comorbidities relevant to covid-19 risk: prevalence and association with 1-year mortality

Comorbidities were common in people with incident cancer: hypertension (83,313 [41.9%]), cardiovascular disease (55,742 [28.0%]), chronic kidney disease (31,935 [16.0%]), obesity (19,589 [9.8%]), type 2 diabetes (18,957 [9.5%]) and COPD (18,373 [9.2%]) (Figure S4). Similar patterns were seen in prevalent cancers (Figure S5). Multimorbidity (≥1 comorbidity) was associated with a higher 1-year mortality (Figure S6 for incident cancers; Figure S7 for prevalent cancers). For example, for incident colorectal cancer, 1-year mortality for 0, 1, 2 and 3+ comorbidities, was 13.8%, 17.3%, 23.6% and 30.2% respectively (Figure S6).

Estimations of total (direct and indirect) excess deaths by cancer site and number of comorbidities over a 1-year period

To ascertain the influence of multimorbidity on total excess deaths, we provide estimates based on 40% (10% infected, 30% affected). For both incident and prevalent cancers, 78% of the predicted excess deaths occur in people with 1+ comorbidity. For example, at RR of 1.2, there are 5,622 excess deaths in those with 1+comorbidity compared to 1,567 in those with no comorbidities (total 7,189) (Figure 4). Even though the size of patient group in 0, 1, 2 and 3+ comorbidities declines (49.8%, 24.7%, 15.0% and 10.6% respectively) the absolute numbers of excess deaths in each comorbidity group are similar, suggesting that patients with comorbidities contribute to a large proportion of excess deaths compared to those without non-cancer comorbidities. For example, at RR of 1.5, the numbers of total excess deaths for both incident and prevalent cancers were 3,922, 4,993, 4,526 and 4,542 in individuals with 0, 1, 2 and 3+ non-cancer comorbidities respectively (Figure 4). The findings for incident and prevalent cases are presented separately in Figure S8 and Figure S9.

We share the underlying study estimates from this paper (online data supplement) and provide an open-access tool for researchers to interact with the model (https://pasea.shinyapps.io/cancer_covid_app/).

For peer review only - http://bmjopen.bmj.com/site/about/guidelines.xhtml

Discussion:

Statement of principal findings

To our knowledge, this is the first study with near real–time evidence of covid-19's negative impact on cancer services at different phases of the pandemic, its potential to lead to significant excess deaths in people with cancer and the substantial role that comorbidities may play in these excess deaths.

Changes in cancer care at different phases of pandemic: We delineate both the nadir and the incomplete recovery of UK cancer services that have resulted from the covid-19 pandemic. We observed profound declines in urgent two-week wait (2WW) referrals for early cancer diagnosis, which have not returned to pre-covid-19 levels. These may reflect patients' deciding not to seek care due to the perceived risk of infection, but may also be in part due to difficulty in securing appointments due to reprioritised health systems[19]. An unintended consequence of this reprioritisation may be excess deaths due to delayed diagnoses, increased emergency presentations, more advanced stage at presentation, and changes in care pathways that adversely affect outcomes. We also observed large declines in chemotherapy attendance, presumably reflecting capacity/resources being redirected to care for infected patients (e.g., to intensive care) and the desire of clinicians and patients to minimise the risks of covid-19 for susceptible cancer patients [10].

Direct (covid-19) excess deaths: It is important to note that our model estimates deaths additional to those that would be expected (without covid-19) in people with cancer. At a RR of 2, we estimate about 9,000 direct covid-19 excess deaths in 1 year in people with cancer but acknowledge there is uncertainty in this estimate. There is increasing concern that those discharged from hospital with covid-19 may have long term, (including fatal) sequelae.

Total (direct and indirect) excess deaths: Based on our observations regarding the adverse effects of cancer service reprioritization, we consider a proportion affected by the pandemic of 40% plausible, if perhaps somewhat conservative. But, given that adverse effects could be more profound (our 2WW referrals data, for example, would suggest this), we present excess deaths for a range of both 40% and 80%. Adding credibility to our estimates, in a survey in April 2020 of 17,000 UK adults, 56% of cancer patients reported that the NHS had cancelled their treatment[38] Overall, we conservatively estimate, at RR of 1.5, that 17,910 total excess deaths for 1 year will occur in patients with cancer, but this could rise to 35,817. We note the degree of uncertainty in the observed RR at different points in the pandemic. Patients affected by changes in cancer services in March–June 2020 may not necessarily directly contribute to an increase in excess indirect deaths in these four months, as the effects on health and mortality outcomes are more likely to occur in a longer time frame.

For peer review only - http://bmjopen.bmj.com/site/about/guidelines.xhtml

Importance of multimorbidity: We demonstrate that the majority (78%) of excess deaths in people with cancer during the covid-19 pandemic occur in people with at least 1 comorbidity. While many of these comorbidities are treatable, services for these conditions have also been affected by the pandemic. For example, 65% of patients with hypertension and 70% of patients with diabetes reported that the NHS had recently cancelled their care, as captured in the same April 2020 survey noted above[38]. Importantly, the pandemic prompts new questions about which cancer patients are most vulnerable and how best to mitigate an individual's personal risk.

Strengths of this study

There are three major strengths of this study. First, the acquisition and deployment of near real-time data to signal the significant adverse impact of the covid-19 pandemic on cancer services and how this has profound implications for cancer diagnostic and treatment pathways. These data were also used to inform and enhance our existing model that estimates excess mortality due to the pandemic. Second, we provide a pan-cancer comorbidity atlas using a population-based 3.8million primary care cohort to underpin estimates of the additional adverse effect of multimorbidity in cancer patients; cancer registry data tend to lack this more comprehensive information. Third, we provide separate estimates of excess deaths for prevalent cancers and incident (newly diagnosed) cancers, because these represent different patterns of risk, treatment priorities, and roles of general practitioner and oncologist.

Weaknesses of this study

Our model has important limitations. First, there is a lack in the literature of studies on clinical cohorts of cancer patients investigating all-cause mortality rates in those with and without infection; such studies are needed in order to obtain better estimates of the direct effects of the pandemic. Second, the primary care health records we used may have missed some cases of cancer and thus underestimated incidence[41]. If so, our estimates of excess deaths may be conservative. The NHS has national linked hospital admissions and cancer registration data with information on stage and details of surgical, chemotherapeutic and radiotherapy treatment of cancer. However, information governance for such data can take months to secure, making data-enabled research and time-sensitive responsive service improvement difficult. Third, we did not have access to data on children. Fourth, we only have access to empirical cancer service change data from eight hospitals in the UK. Whilst the data may be a representative sample of the UK population, and patterns of decline in service change is corroborated in another study[42], more widespread access to other trusts may be beneficial to ascertain national and regional effects.

Implications for clinicians and policymakers

For peer review only - http://bmjopen.bmj.com/site/about/guidelines.xhtml

Our study may inform decision-making at three levels. First, from a healthcare policy and healthcare implementation perspective, it is clear that the NHS cannot simply be ‘switched on’ again at full capacity for hospital or primary care services as there will be a significant backlog of untreated patients, with waiting lists predicted to expand to 10 million patients. Data published on June 13th 2020 indicate ~100,000 “missing” cancer referrals in April 2020 alone[7]. More granular weekly intelligence from the centres contributing data to this study suggests that this negative impact will continue for at least six to nine months, placing many more patients at risk.

Second, there are currently no accessible national systems available for near real-time data on care and outcomes of cancer patients. Our study suggests that we should expand our near real-time data approach across the UK to collect actionable information on (i) death certification – in particular distinguishing the contribution of cancer, comorbid conditions and covid-19 to death; (ii) cancer health services activity data, to monitor how changes at each phase of the pandemic (including clearing backlogs for under-referral, under-diagnosis and under-treatment) might influence future health outcomes and (iii) treatment services data for non-malignant comorbidities of cancer patients, such as cardiovascular disease, diabetes and hypertension.

Third, with knowledge of mortality risk based on type of cancer, age and comorbidities that we provide in an online format (https://pasea.shinyapps.io/cancer_covid_app/), supplemented with local knowledge of health service resilience, we propose that weekly indicators and warnings for vulnerable cancer patients with multimorbidity could be provided. Using this intelligence, treatment prioritization as we resume cancer services could be enhanced by patient-specific risk/benefit assessments which include multimorbidity, particularly in situations where treatment provision outweighs non-treatment/safety issues related to covid-19[19].

Unanswered questions and future research

There are important areas for further research. First, there is a need for long-term (1 to 5 years) monitoring of the extent to which cancer patients experience excess mortality due to the pandemic. We chose a 1-year time horizon, because the adverse consequences on health are likely to extend beyond the initial wave of the pandemic. But its impact on excess mortality in patients with cancer, particularly those whose diagnosis/treatment is delayed, may take years to understand. The specific impact of paused cancer screening, particularly for breast and colorectal cancer may be profound. The social and psychological consequences of physical distancing on mortality may also be particularly important in cancer[43,44], while international studies across 75 countries signpost how unemployment negatively impacts mortality in cancer patients[45]. Hence, the socio-economic effects of the current pandemic are likely to last for a considerable period beyond one year[46]. As new empirical data become available on heath service, social/psychological and economic changes,

For peer review only

For peer review only - http://bmjopen.bmj.com/site/about/guidelines.xhtml

our model can better specify the proportion and type of cancer patients thus affected and look to develop appropriate mitigation strategies.

Conclusion

We mobilised usually inaccessible near real-time hospital data to quantify the immediate adverse impacts of the covid-19 pandemic on cancer services, on people who may demonstrate symptoms of cancer and on patients who are being treated for cancer. The marked reductions observed in the demand for, and supply of, cancer services have only partially recovered with lockdown easing. Such perturbations in cancer care may contribute, over a 1-year time horizon, to substantial excess mortality among people with cancer and multimorbidity. There is an urgent need to better understand and mitigate these excess mortality risks, some of which may be revealed only over the longer term.

For peer review only

For peer review only - http://bmjopen.bmj.com/site/about/guidelines.xhtml

Contributorship statement:

Research question: AGL, HH

Funding: AGL, AB, ML, HH, DATA-CAN

Study design and analysis plan: AGL, LP, AB, MK, WHC, HH

Preparation of data, including electronic health record phenotyping in the CALIBER portal: AGL, LP, SD

Provision of weekly hospital data: GH, KPJ, MDF, DH, ML, KB, CD

Statistical analysis: AGL, LP, WHC, MK

Drafting initial versions of manuscript: AGL, ML, HH

Drafting final versions of manuscript: AGL, GH, CD, ML, HH,

Critical review of early and final versions of manuscript: All authors

The corresponding author attests that all listed authors meet authorship criteria and that no others meeting the criteria have been omitted.

Declaration of interests:

ML has received honoraria from Pfizer, EMD Serono and Roche for presentations unrelated to this research. ML has received an unrestricted educational grant from Pfizer for research unrelated to the research presented in this paper. AB has received research funding from AstraZeneca. MDF has received research funding from AstraZeneca, Boehringer Ingelheim, Merck and MSD and honoraria from Achilles, AstraZeneca, Bayer, Boehringer Ingelheim, Bristol-Meyers Squibb, Celgene, Guardant Health, Merck, MSD, Nanobiotix, Novartis, Pharmamar, Roche and Takeda for advisory roles or presentations unrelated to this research. GRF receives funding from companies that manufacture drugs for hepatitis C virus (AbbVie, Gilead, MSD) and consult for GSK, Arbutus and Shionogi in areas unrelated to this research.

Acknowledgments:

We thank Tony Hagger, Shiva Thapa, Mohammed Emran, Cara Anderson, Louise Herron, Joy Beaumont, Maurice Loughrey, Philip Melling and Lee Cogger for their help on collating data on urgent cancer referrals and chemotherapy attendances. We thank the HDR UK DATA-CAN Patient and Public Involvement and Engagement panel for critical feedback on the manuscript. We thank Charles Swanton for his valuable comments on the manuscript. This study is based in part on data from the Clinical Practice Research Datalink obtained under licence from the UK Medicines and Healthcare products Regulatory Agency. The data is provided by patients and collected by the NHS as part of their care and support. Mortality data are from the Office for National Statistics (ONS).

Funding statement:

We acknowledge Health Data Research UK (HDR UK) support for the HDR UK substantive sites involved in this research (HDR London, HDR Wales and Northern Ireland) and DATA-CAN. DATA-

peer review only

For peer review only - http://bmjopen.bmj.com/site/about/guidelines.xhtml

CAN is part of the Digital Innovation Hub Programme, delivered by HDR UK and funded by UK Research and Innovation through the government's Industrial Strategy Challenge Fund (ISCF). AGL is supported by funding from the Wellcome Trust, National Institute for Health Research (NIHR) University College London Hospitals, NIHR Great Ormond Street Hospital Biomedical Research Centres and the Health Data Research UK Better Care Catalyst Award. AB is supported by research funding from NIHR, British Medical Association, Astra-Zeneca and UK Research and Innovation. KPJ is supported by the NIHR Great Ormond Street Hospital Biomedical Research Centre. CD and KPJ is funded by UCLPartners. HH is an NIHR Senior Investigator and is funded by the NIHR University College London Hospitals Biomedical Research Centre, supported by Health Data Research UK (grant No. LOND1), which is funded by the UK Medical Research Council, Engineering and Physical Sciences Research Council, Economic and Social Research Council, Department of Health and Social Care (England), Chief Scientist Office of the Scottish Government Health and Social Care Directorates, Health and Social Care Research and Development Division (Welsh Government), Public Health Agency (Northern Ireland), British Heart Foundation, Wellcome Trust, The BigData@Heart Consortium, funded by the Innovative Medicines Initiative-2 Joint Undertaking under grant agreement No. 116074.

Data sharing statement:

Data used in this study was accessed through the Clinical Practice Research Datalink that is subject to protocol approval by an Independent Scientific Advisory Committee and cannot directly be shared. All results are reported in the manuscript and no additional data is available.

Patient and public involvement statement:

The Health Data Research UK hub for cancer (DATA-CAN) patient and public advisory panel were consulted during the writing of this manuscript.

Ethics approval:

The study was approved by the MHRA (UK) Independent Scientific Advisory Committee (20_074R2), under Section 251 (NHS Social Care Act 2006).

For peer review only

For peer review only - http://bmjopen.bmj.com/site/about/guidelines.xhtml

References:

1 Banerjee A, Pasea L, Harris S, *et al.* Estimating excess 1-year mortality associated with the COVID-19 pandemic according to underlying conditions and age: a population-based cohort study. *Lancet* 2020;**395**:1715–25. doi:10.1016/S0140-6736(20)30854-0

2 Kuderer NM, Choueiri TK, Shah DP, *et al.* Clinical impact of COVID-19 on patients with cancer (CCC19): a cohort study. *Lancet* 2020.

3 Williamson E, Walker AJ, Bhaskaran KJ, *et al.* OpenSAFELY: factors associated with COVID-19-related hospital death in the linked electronic health records of 17 million adult NHS patients. *MedRxiv* 2020.

4 Rosenbaum L. The Untold Toll — The Pandemic's Effects on Patients without Covid-19. *N Engl J Med* doi:10.1056/NEJMms2009984

5 Vrdoljak E, Sullivan R, Lawler M. Cancer and COVID-19; how do we manage cancer optimally through a public health crisis? *Eur J Cancer* 2020;**132**:P98-99.

6 Thomson DJ, Palma D, Guckenberger M, *et al.* Practice recommendations for risk-adapted head and neck cancer radiotherapy during the COVID-19 pandemic: an ASTRO-ESTRO consensus statement. *Int J Radiat Oncol Biol Phys* 2020.

7 NHS England. Provider-based Cancer Waiting Times for April 2019. Accessed 17 June 2020. https://www.england.nhs.uk/statistics/statistical-work-areas/cancer-waiting-times/monthly-prov-cwt/2019-20-monthly-provider-cancer-waiting-times-statistics/provider-based-cancer-waiting-times-for-april-2019-provisional/

8 Burki TK. Cancer guidelines during the COVID-19 pandemic. *Lancet Oncol* 2020.

9 British Society of Gastroenterology. Endoscopy activity and COVID-19: BSG and JAG guidance. Accessed 19 April 2020. https://www.bsg.org.uk/covid-19-advice/endoscopy-activity-and-covid-19-bsg-and-jag-guidance/

10 National Institute for Health and Care Excellence. COVID-19 rapid guideline: delivery of systemic anticancer treatments. Accessed 19 April 2020. https://www.nice.org.uk/guidance/ng161/chapter/6-Prioritising-systemic-anticancer-treatments

11 American Cancer Society. Survey: COVID-19 Affecting Patients' Access to Cancer Care. Accessed 19 April 2020. https://www.fightcancer.org/releases/survey-covid-19-affecting-patients'-access-cancer-care-5

12 American College of Surgeons. COVID-19 Guidelines for Triage of Breast Cancer Patients. Accessed 19 April 2020. https://www.facs.org/covid-19/clinical-guidance/elective-case/breast-cancer

13 American Society for Radiation Oncology. COVID-19 Coding Guidance. Accessed 19 April 2020. https://www.astro.org/Daily-Practice/Coding/Coding-Guidance/Coding-Updates/COVID-19-Coding-Guidance

14 Sud A, Jones M, Broggio J, *et al.* Collateral damage: the impact on outcomes from cancer

For peer review only - http://bmjopen.bmj.com/site/about/guidelines.xhtml

surgery of the COVID-19 pandemic. *Ann Oncol* 2020.

15 Sud A, Jones ME, Broggio J, *et al.* Quantifying and mitigating the impact of the COVID-19 pandemic on outcomes in colorectal cancer. 2020.

16 Lee LYW, Cazier JB, Starkey T, *et al.* COVID-19 mortality in patients with cancer on chemotherapy or other anticancer treatments: a prospective cohort study. *Lancet* 2020.

17 Public Health England. Guidance on shielding and protecting people defined on medical grounds as extremely vulnerable from COVID-19. Accessed 19 April 2020. https://www.gov.uk/government/publications/guidance-on-shielding-and-protecting-extremely-vulnerable-persons-from-covid-19/guidance-on-shielding-and-protecting-extremely-vulnerable-persons-from-covid-19

18 Public Health England. Guidance on social distancing for everyone in the UK. Accessed 19 April 2020. https://www.gov.uk/government/publications/covid-19-guidance-on-social-distancing-and-for-vulnerable-people/guidance-on-social-distancing-for-everyone-in-the-uk-and-protecting-older-people-and-vulnerable-adults

19 Hanna TP, Evans GA, Booth CM. Cancer, COVID-19 and the precautionary principle: prioritizing treatment during a global pandemic. *Nat Rev Clin Oncol* 2020;:1–3.

20 Renzi C, Kaushal A, Emery J, *et al.* Comorbid chronic diseases and cancer diagnosis: disease-specific effects and underlying mechanisms. *Nat Rev Clin Oncol* Published Online First: 2019. doi:10.1038/s41571-019-0249-6

21 Health Data Research UK. DATA-CAN - The Health Data Research Hub for Cancer. Accessed 20 April 2020. https://www.hdruk.ac.uk/infrastructure/the-hubs/data-can/

22 George J, Mathur R, Shah AD, *et al.* Ethnicity and the first diagnosis of a wide range of cardiovascular diseases: Associations in a linked electronic health record cohort of 1 million patients. *PLoS One* 2017;**12**:1–17. doi:10.1371/journal.pone.0178945

23 Bhaskaran K, Forbes HJ, Douglas I, *et al.* Representativeness and optimal use of body mass index (BMI) in the UK Clinical Practice Research Datalink (CPRD). *BMJ Open* 2013;**3**:e003389.

24 Mathur R, Bhaskaran K, Chaturvedi N, *et al.* Completeness and usability of ethnicity data in UK-based primary care and hospital databases. *J Public Health (Bangkok)* 2014;**36**:684–92.

25 Herrett E, Thomas SL, Schoonen WM, *et al.* Validation and validity of diagnoses in the General Practice Research Database: a systematic review. *Br J Clin Pharmacol* 2010;**69**:4–14.

26 Denaxas S, Gonzalez-Izquierdo A, Direk K, *et al.* UK phenomics platform for developing and validating electronic health record phenotypes: CALIBER. *J Am Med Informatics Assoc* 2019.

27 Denaxas SC, George J, Herrett E, *et al.* Data resource profile: cardiovascular disease research using linked bespoke studies and electronic health records (CALIBER). *Int J Epidemiol* 2012;**41**:1625–38.

For peer review only

For peer review only - http://bmjopen.bmj.com/site/about/guidelines.xhtml

28 Kuan V, Denaxas S, Gonzalez-Izquierdo A, *et al.* A chronological map of 308 physical and mental health conditions from 4 million individuals in the English National Health Service. *Lancet Digit Heal* 2019;**1**:e63–77. doi:10.1016/S2589-7500(19)30012-3

29 Shah AD, Langenberg C, Rapsomaniki E, *et al.* Type 2 diabetes and incidence of cardiovascular diseases: A cohort study in 1·9 million people. *Lancet Diabetes Endocrinol* 2015;**3**:105–13. doi:10.1016/S2213-8587(14)70219-0

30 Rapsomaniki E, Timmis A, George J, *et al.* Blood pressure and incidence of twelve cardiovascular diseases: Lifetime risks, healthy life-years lost, and age-specific associations in 1·25 million people. *Lancet* 2014;**383**:1899–911. doi:10.1016/S0140-6736(14)60685-1

31 Pikoula M, Quint JK, Nissen F, *et al.* Identifying clinically important COPD sub-types using data-driven approaches in primary care population based electronic health records. *BMC Med Inform Decis Mak* 2019;**19**:86.

32 Pujades-Rodriguez M, Duyx B, Thomas SL, *et al.* Rheumatoid arthritis and incidence of twelve initial presentations of cardiovascular disease: a population record-linkage cohort study in England. *PLoS One* 2016;**11**.

33 Dai M, Liu D, Liu M, *et al.* Patients with cancer appear more vulnerable to SARS-COV-2: a multi-center study during the COVID-19 outbreak. *Cancer Discov* Published Online First: 2020. doi:10.1158/2159-8290.CD-20-0422

34 Office for National Statistics. Coronavirus (COVID-19) Infection Survey. https://www.ons.gov.uk/peoplepopulationandcommunity/healthandsocialcare/conditionsanddiseases/datasets/coronaviruscovid19infectionsurveydata. Accessed 3 September 2020.

35 GOV UK. Sero-surveillance of COVID-19. https://www.gov.uk/government/publications/national-covid-19-surveillance-reports/sero-surveillance-of-covid-19. Accessed 3 September 2020.

36 Valenti L, Bergna A, Pelusi S, *et al.* SARS-CoV-2 seroprevalence trends in healthy blood donors during the COVID-19 Milan outbreak. *medRxiv* 2020.

37 Salje H, Kiem CT, Lefrancq N, *et al.* Estimating the burden of SARS-CoV-2 in France. *Science (80-)* 2020.

38 Understanding Society COVID-19 survey. Accessed 17 June 2020. https://www.understandingsociety.ac.uk/sites/default/files/downloads/general/ukhls_briefingnote_covid_health_final.pdf.

39 Office for National Statistics. Estimates of the population for the UK, England and Wales, Scotland and Northern Ireland. Accessed 23 April 2020. https://www.ons.gov.uk/peoplepopulationandcommunity/populationandmigration/populationestimates/datasets/populationestimatesforukenglandandwalesscotlandandnorthernireland

40 NHS England. NHS warning to seek help for cancer symptoms, as half of public report concerns with getting checked. https://tinyurl.com/y6owtj4u.

41 Margulis A V, Fortuny J, Kaye JA, *et al.* Validation of cancer cases using primary care,

For peer review only

For peer review only - http://bmjopen.bmj.com/site/about/guidelines.xhtml

cancer registry, and hospitalization data in the United Kingdom. *Epidemiology* 2018;**29**:308.

42 Banerjee A, Chen S, Pasea L, *et al.* Excess deaths in people with cardiovascular diseases during the COVID-19 pandemic. *medRxiv* Published Online First: 2020. doi:10.1101/2020.06.10.20127175

43 Kroenke CH, Kubzansky LD, Schernhammer ES, *et al.* Social networks, social support, and survival after breast cancer diagnosis. *J Clin Oncol* 2006;**24**:1105–11.

44 Elovainio M, Hakulinen C, Pulkki-Råback L, *et al.* Contribution of risk factors to excess mortality in isolated and lonely individuals: an analysis of data from the UK Biobank cohort study. *Lancet Public Heal* 2017;**2**:e260--e266.

45 Maruthappu M, Watkins J, Noor AM, *et al.* Economic downturns, universal health coverage, and cancer mortality in high-income and middle-income countries, 1990--2010: a longitudinal analysis. *Lancet* 2016;**388**:684–95.

46 Longer-Run Economic Consequences of Pandemics. Accessed 1 May 2020. https://www.frbsf.org/economic-research/files/wp2020-09.pdf

For peer review only

For peer review only - http://bmjopen.bmj.com/site/about/guidelines.xhtml

Figure legends:

Figure 1. Weekly hospital data (January 2019 to June 2020) on changes in urgent referrals and chemotherapy clinic attendance from eight hospitals in the UK mapped to phases of the pandemic. Weekly changes from January 2020 to June 2020 were mapped to phases of the pandemic. Weekly values were plotted as percentage increase or decrease relative to the 2019 average. The data for Northern Ireland includes five Health and Social Care Trusts (HSCs) that cover all health service provision in Northern Ireland: Belfast HSC, Northern HSC, South Eastern HSC, Southern HSC and Western HSC. Vertical dotted lines indicate the Christmas Bank Holiday.

Figure 2. Office for National Statistics data on weekly registrations of deaths in the England and Wales from 3 January 2020 to 15 May 2020. (A) Upper panel indicates the number of weekly deaths. (B) Lower panel indicates weekly changes in relative risk estimates calculated by comparing the current weekly deaths to 5-year weekly averages. Dates indicate week ending on a particular date.

Figure 3. Estimated total (direct and indirect) excess deaths by cancer site over a 1-year period (A) 1-year mortality for incident and prevalent cancers. The whiskers are 95% confidence intervals. (B) Total excess deaths were scaled up to the population of England aged 30+ consisting of 35 million individuals using England mortality estimates for both incident and prevalent cancers combined. We estimated direct excess deaths at a 10% infection rate. We estimated total (direct and indirect) excess deaths for 40% (10% infected, 30% affected) and 80% (10% infected, 70% affected) of the population.

Figure 4. Total (direct and indirect) excess deaths for both incident and prevalent cancers by cancer site and number of comorbidities over a 1-year period. Stacked bar chart indicates the proportion of individuals with 0, 1, 2 and 3+ comorbidities by cancer site. We estimated total excess deaths for 40% (10% infected, 30% affected) of the population. Total excess deaths were scaled up to the population of England aged 30+ consisting of 35 million individuals using England mortality estimates for both incident and prevalent cancers combined.

Supplementary figure legends:

Figure S1. Age-adjusted incidence rates for 24 primary cancers from England and the UK (International Agency for Research on Cancer [IARC]) For England data, cervix refers to both carcinoma in situ of cervix and primary malignancy of cervix. For IARC data, only cervix uteri are included. CRUK: Cancer Research UK.

For peer review only - http://bmjopen.bmj.com/site/about/guidelines.xhtml

Figure S2. Total excess deaths for incident cancers over a 1-year period scaled up to the population of England aged 30+ consisting of 35 million individuals using England mortality estimates. We estimated direct excess deaths at a 10% infection rate. We estimated total (direct and indirect) excess deaths for 40% (10% infected, 30% affected) and 80% (10% infected, 70% affected) of the population.

Figure S3. Total excess deaths for prevalent cancers over a 1-year period scaled up to the population of England aged 30+ consisting of 35 million individuals using England mortality estimates. We estimated direct excess deaths at a 10% infection rate. We estimated total (direct and indirect) excess deaths for 40% (10% infected, 30% affected) and 80% (10% infected, 70% affected) of the population.

For peer review only

Figure S4. Proportion of patients with any of the 15 comorbidity clusters by cancer site for incident cancers (N=199,057) in a population of 3,862,012 adults in England. Age indicates mean age at diagnosis.

Figure S5. Proportion of patients with any of the 15 comorbidity clusters by cancer site for prevalent cancers (N=117,978) in a population of 3,862,012 adults in England. Age indicates mean age at diagnosis.

Figure S6. Forest plot of background (pre-COVID-19) 1-year cancer mortality for incident cases according to cancer site and number of underlying comorbidities in England. The whiskers are 95% confidence intervals.

Figure S7. Forest plot of background (pre-COVID-19) 1-year cancer mortality for prevalent cases according to cancer site and number of underlying comorbidities in England. The whiskers are 95% confidence intervals.

Figure S8. Total (direct and indirect) excess deaths for incident cancers by cancer site and number of comorbidities over a 1-year period. Stacked bar chart indicates the proportion of individuals with 0, 1, 2 and 3+ comorbidities by cancer site. We estimated total excess deaths for 40% (10% infected, 30% affected) of the population. Total excess deaths were scaled up to the population of England aged 30+ consisting of 35 million individuals using England mortality estimates for incident cancers.

Figure S9. Total (direct and indirect) excess deaths for prevalent cancers by cancer site and number of comorbidities over a 1-year period. Stacked bar chart indicates the proportion of individuals with 0, 1, 2 and 3+ comorbidities by cancer site. We estimated total excess deaths for 40% (10% infected,

For peer review only - http://bmjopen.bmj.com/site/about/guidelines.xhtml

30% affected) of the population. Total excess deaths were scaled up to the population of England aged 30+ consisting of 35 million individuals using England mortality estimates for prevalent cancers.

For peer review only

For peer review only - http://bmjopen.bmj.com/site/about/guidelines.xhtml

Supplementary Methods

Description on 15 comorbidity clusters relevant to COVID-19

The PHE list included chronic respiratory disease, chronic heart disease, immunocompromised individuals, HIV, use of corticosteroids, obesity, diabetes, chronic kidney disease, chronic liver disease, chronic neurological disorders and splenic disorders. We have performed analyses for all the above conditions and have additionally considered hypertension, Crohn's disease, cystic fibrosis and rheumatoid arthritis. Given that condition clusters such as **(i)** chronic heart disease would involve a range of conditions, we have derived composite variables to include 15 conditions considered as cardiovascular disease (CVD) that included acute myocardial infarction, unstable angina, chronic stable angina, heart failure, cardiac arrest or sudden coronary death, transient ischemic attack, intracerebral haemorrhage, subarachnoid haemorrhage, ischemic stroke, abdominal aortic aneurysm, peripheral arterial disease, atrial fibrillation, congenital heart disease, hypertrophic and dilated cardiomyopathy and valve disease (multiple, mitral and aortic)[29]. We also considered **(ii)** Hypertension, defined as ≥140 mmHg systolic blood pressure (or ≥150 mmHg for people aged ≥60 years without diabetes and chronic kidney disease) and/or ≥90 mmHg diastolic blood pressure[30], **(iii)** type 2 diabetes, **(iv)** obesity, defined as a body mass index of ≥40kg/m^2, **(v)** chronic kidney disease (CKD), **(vi)** chronic obstructive pulmonary disease (COPD)[31], **(vii)** patients on immunosuppressive drugs (not cancer chemotherapy), **(viii)** patients with HIV or corticosteroid prescription, **(ix)** chronic neurological disorders, defined as a composite of Parkinson's disease, motor neuron disease, learning disability and cerebral palsy, **(x)** multiple sclerosis separately, **(xi)** splenic disorders, defined as a composite of splenomegaly, splenectomy and hyposplenism, **(xii)** chronic liver diseases, defined as a composite of chronic viral hepatitis B or C, primary biliary cholangitis, liver fibrosis, liver cirrhosis and non-alcoholic fatty liver disease, **(xiii)** Crohn's disease, **(xiv)** cystic fibrosis and **(xv)** rheumatoid arthritis[32].

For peer review only

For peer review only - http://bmjopen.bmj.com/site/about/guidelines.xhtml

Figure 1. Weekly hospital data (January 2019 to June 2020) on changes in urgent referrals and chemotherapy clinic attendance from eight hospitals in the UK mapped to phases of the pandemic. Weekly changes from January 2020 to June 2020 were mapped to phases of the pandemic. Weekly values were plotted as percentage increase or decrease relative to the 2019 average. The data for Northern Ireland includes five Health and Social Care Trusts (HSCs) that cover all health service provision in Northern Ireland: Belfast HSC, Northern HSC, South Eastern HSC, Southern HSC and Western HSC. Vertical dotted lines indicate the Christmas Bank Holiday.

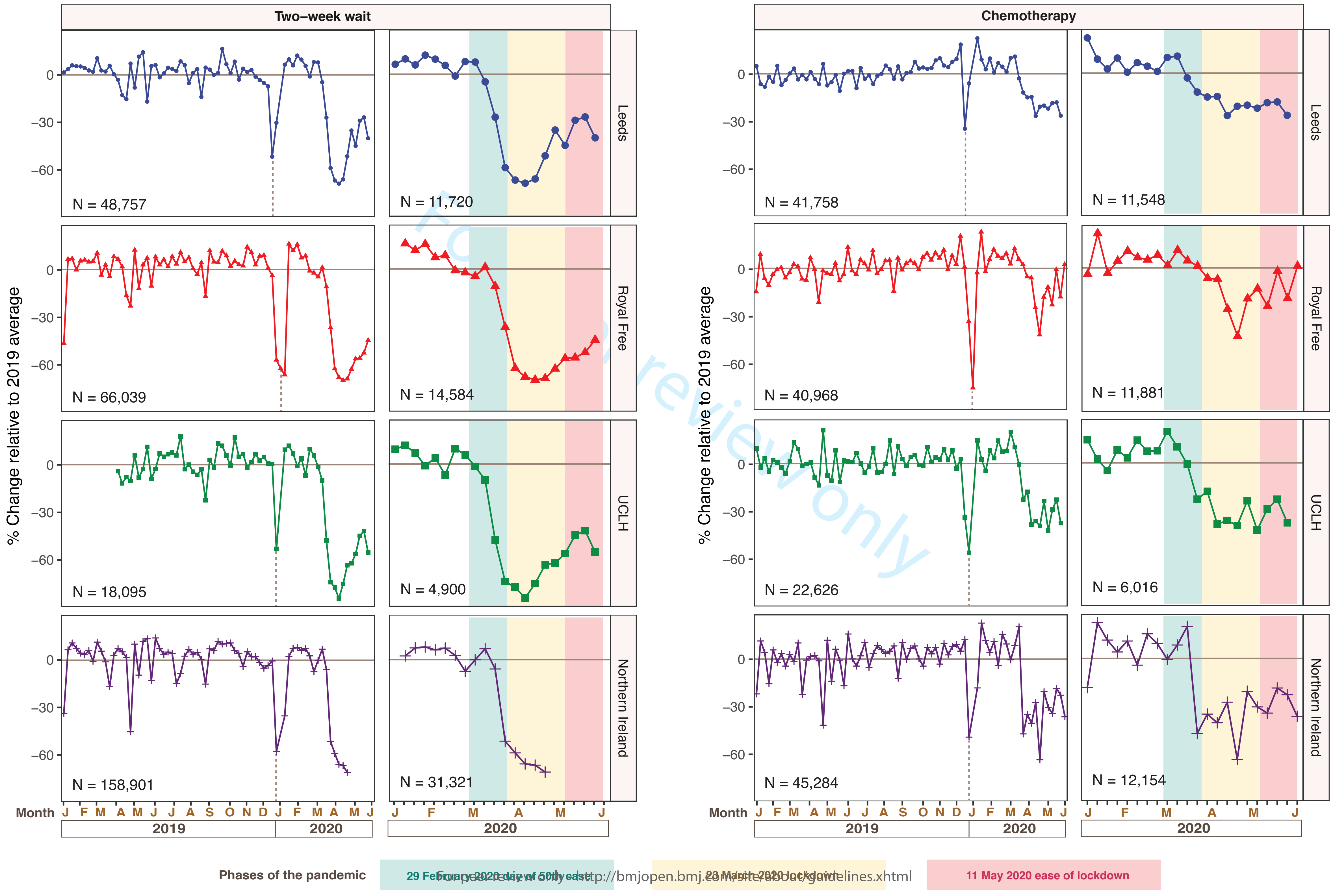

Figure 2. Office for National Statistics data on weekly registrations of deaths in the England and Wales from 3 January 2020 to 15 May 2020. (A) Upper panel indicates the number of weekly deaths. (B) Lower panel indicates weekly changes in relative risk estimates calculated by comparing the current weekly deaths to 5-year weekly averages. Dates indicate week ending on a particular date.

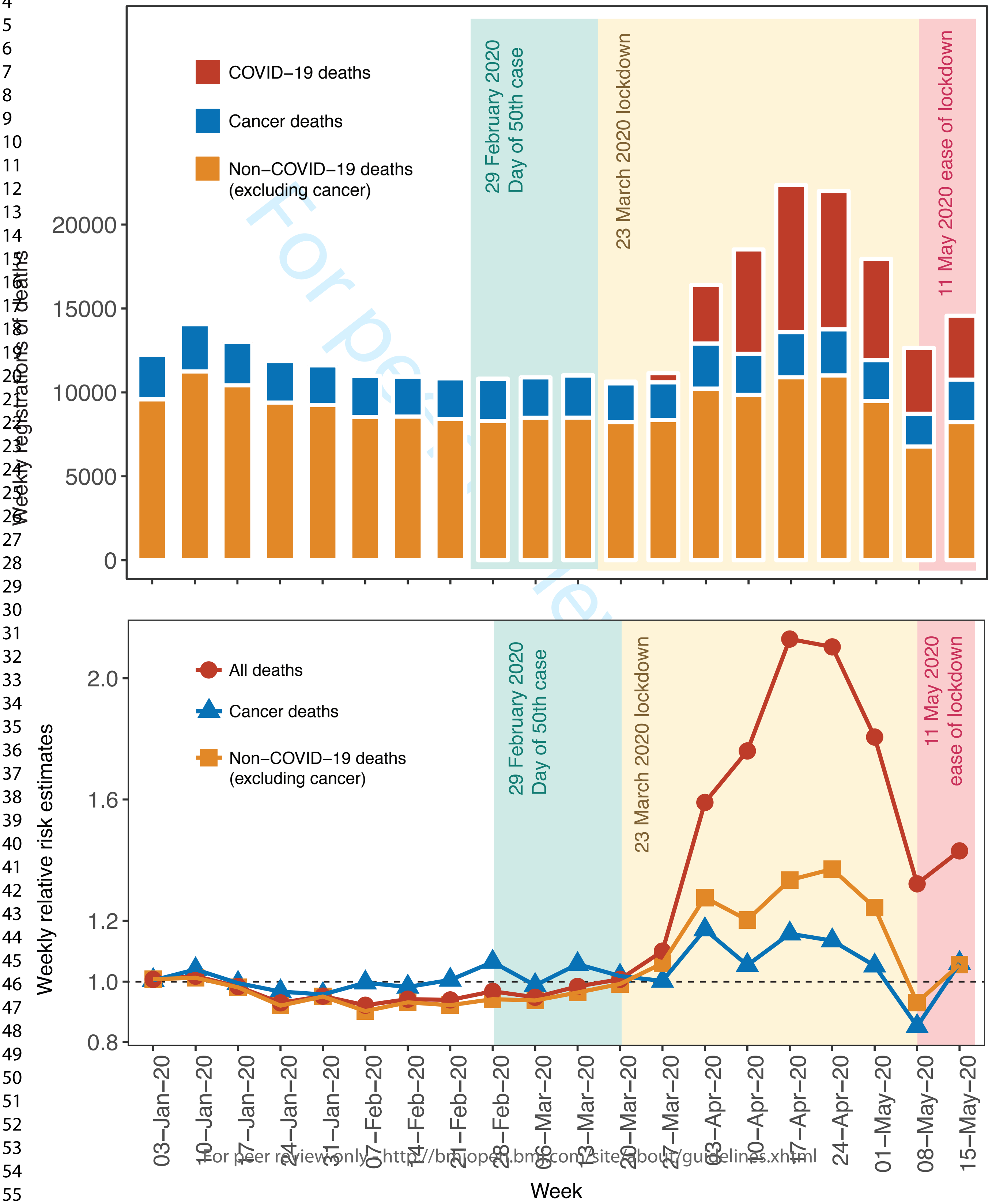

Figure 3. Estimated total (direct and indirect) excess deaths by cancer site over a 1-year period (A) 1-year mortality for incident and prevalent cancers. The whiskers are 95% confidence intervals. (B) Total excess deaths were scaled up to the population of England aged 30+ consisting of 35 million individuals using England mortality estimates for both incident and prevalent cancers combined. We estimated direct excess deaths at a 10% infection rate. We estimated total (direct and indirect) excess deaths for 40% (10% infected, 30% affected) and 80% (10% infected, 70% affected) of the population.

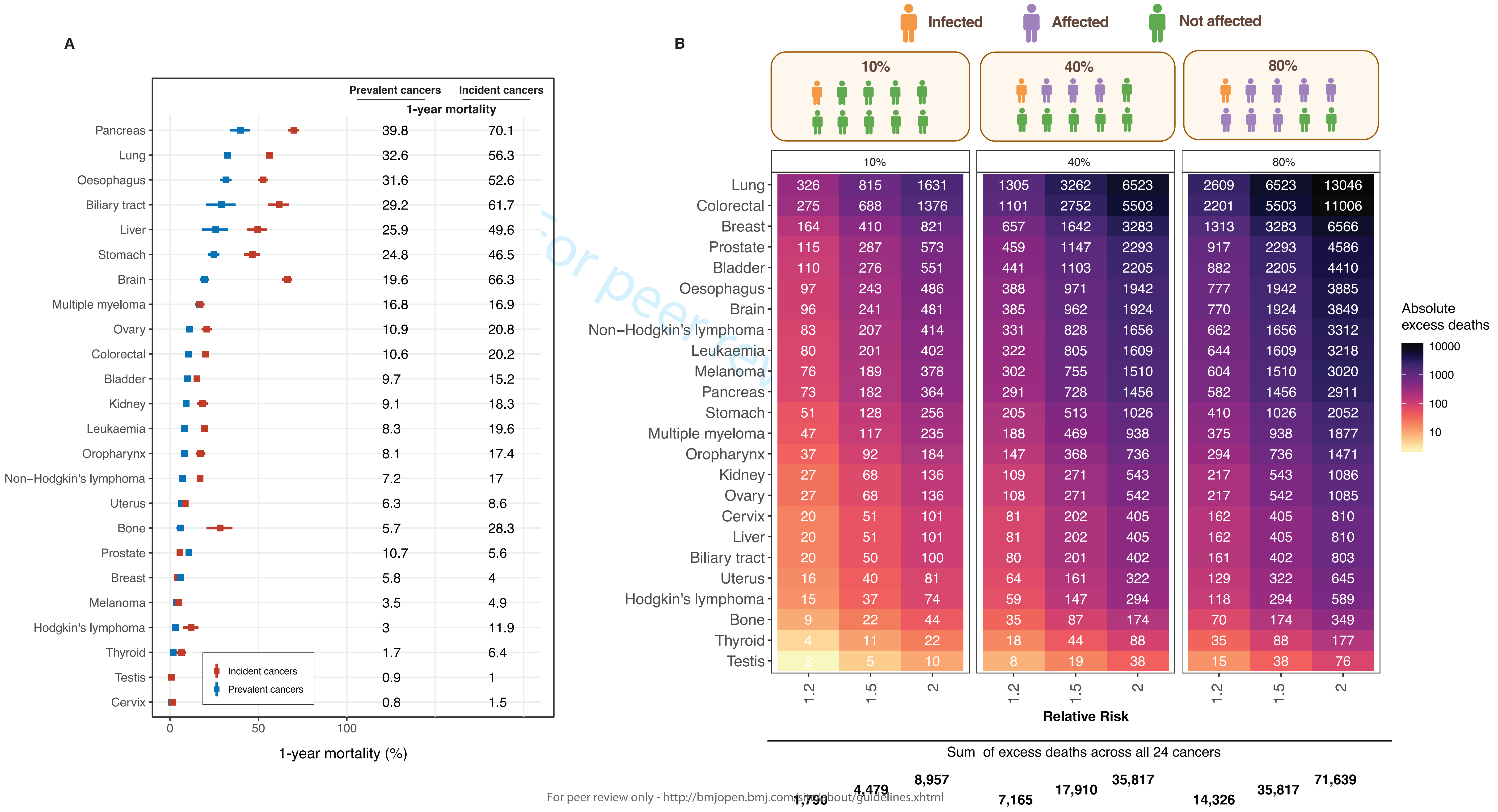

Figure 4. Total (direct and indirect) excess deaths for both incident and prevalent cancers by cancer site and number of comorbidities over a 1-year period. Stacked bar chart indicates the proportion of individuals with 0, 1, 2 and 3+ comorbidities by cancer site. We estimated total excess deaths for 40% (10% infected, 30% affected) of the population. Total excess deaths were scaled up to the population of England aged 30+ consisting of 35 million individuals using England mortality estimates for both incident and prevalent cancers combined.

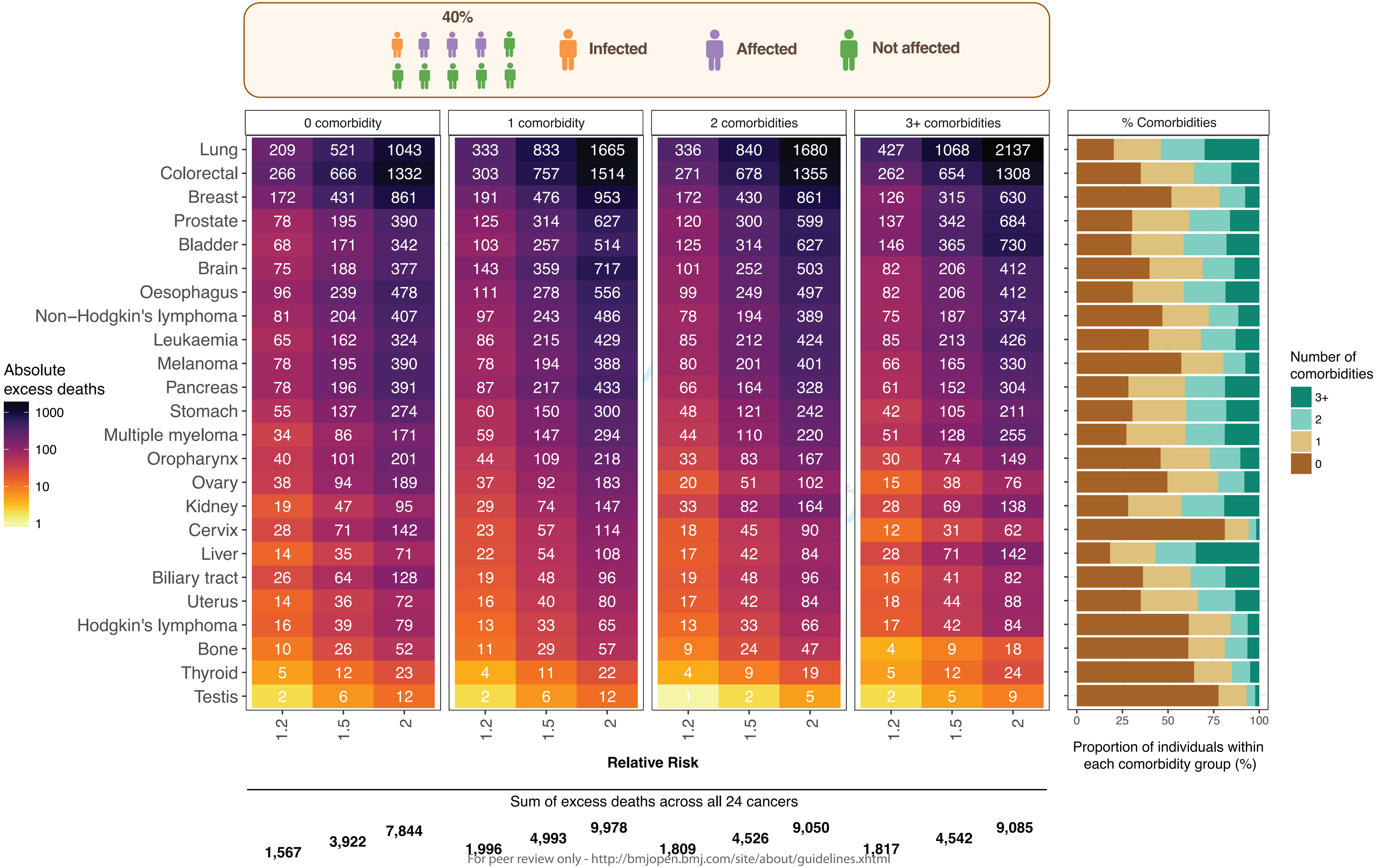

For peer review only - http://bmjopen.bmj.com/site/about/guidelines.xhtml

Figure S1. Age-adjusted incidence rates for 24 primary cancers from England and the UK (International Agency for Research on Cancer [IARC]) For England data, cervix refers to both carcinoma in situ of cervix and primary malignancy of cervix. For IARC data, only cervix uteri are included. CRUK: Cancer Research UK.

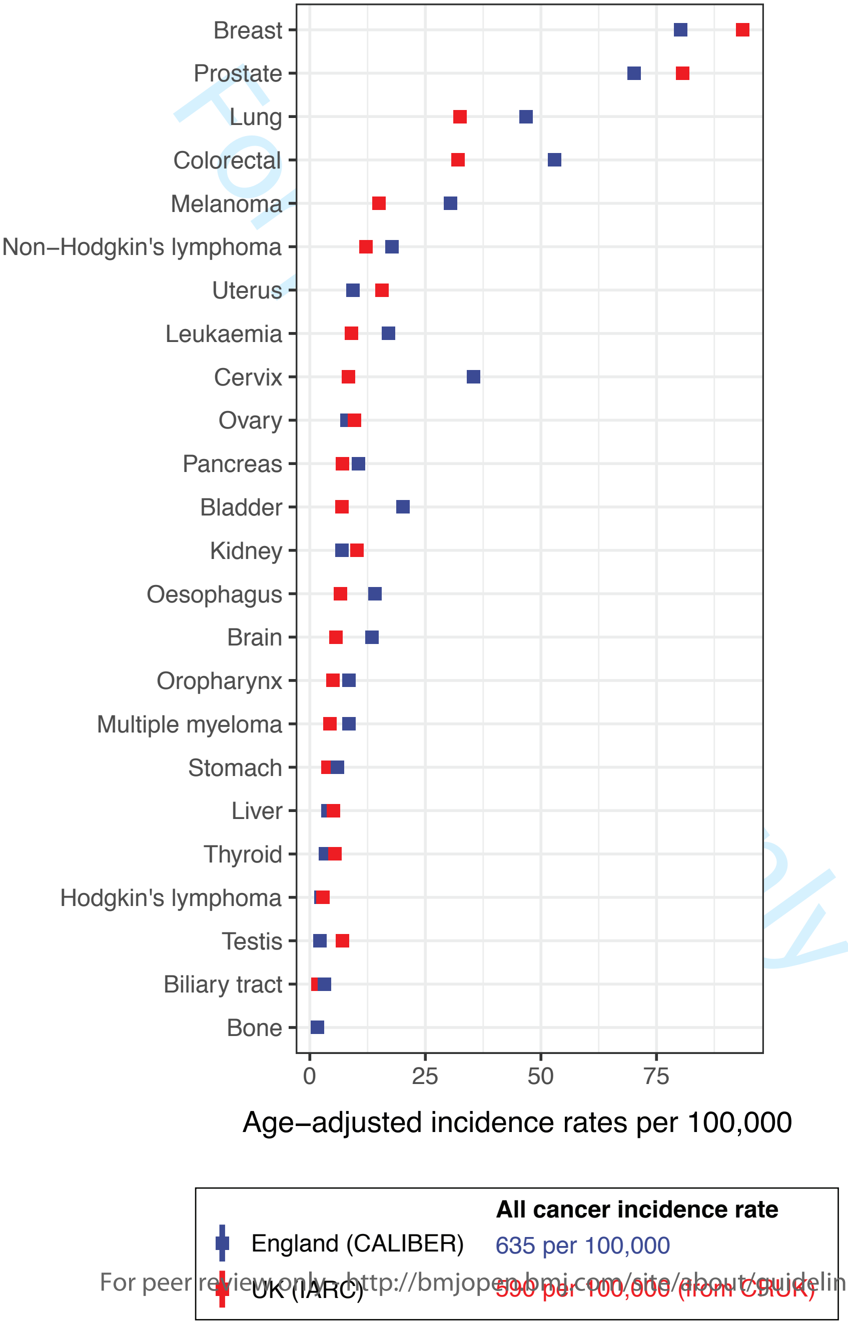

For peer review only - http://bmjopen.bmj.com/site/about/guidelines.xhtml

Figure S2. Total excess deaths for incident cancers over a 1-year period scaled up to the population of England aged 30+ consisting of 35 million individuals using England mortality estimates. We estimated direct excess deaths at a 10% infection rate. We estimated total (direct and indirect) excess deaths for 40% (10% infected, 30% affected) and 80% (10% infected, 70% affected) of the population.

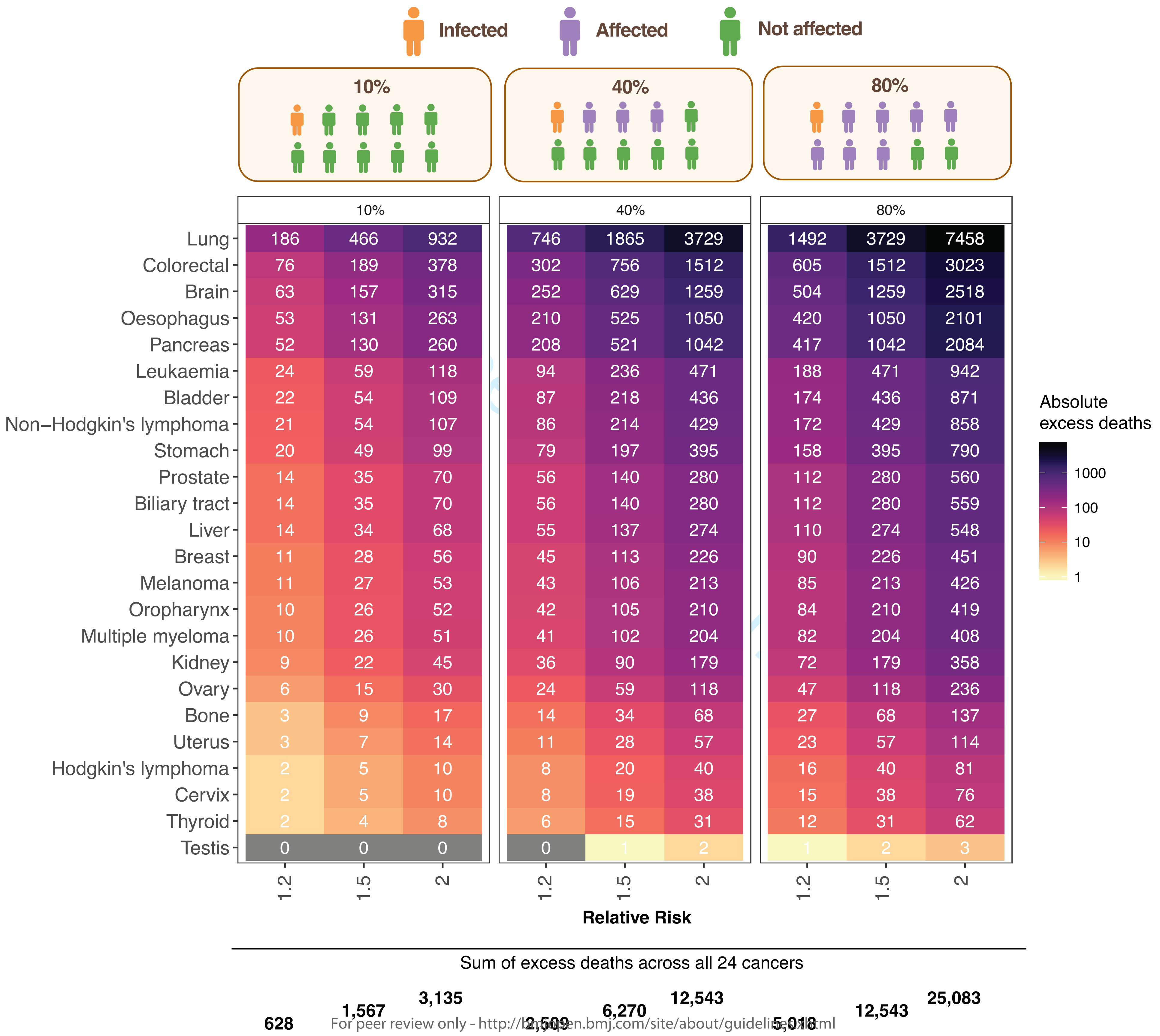

Figure S3. Total excess deaths for prevalent cancers over a 1-year period scaled up to the population of England aged 30+ consisting of 35 million individuals using England mortality estimates. We estimated direct excess deaths at a 10% infection rate. We estimated total (direct and indirect) excess deaths for 40% (10% infected, 30% affected) and 80% (10% infected, 70% affected) of the population.

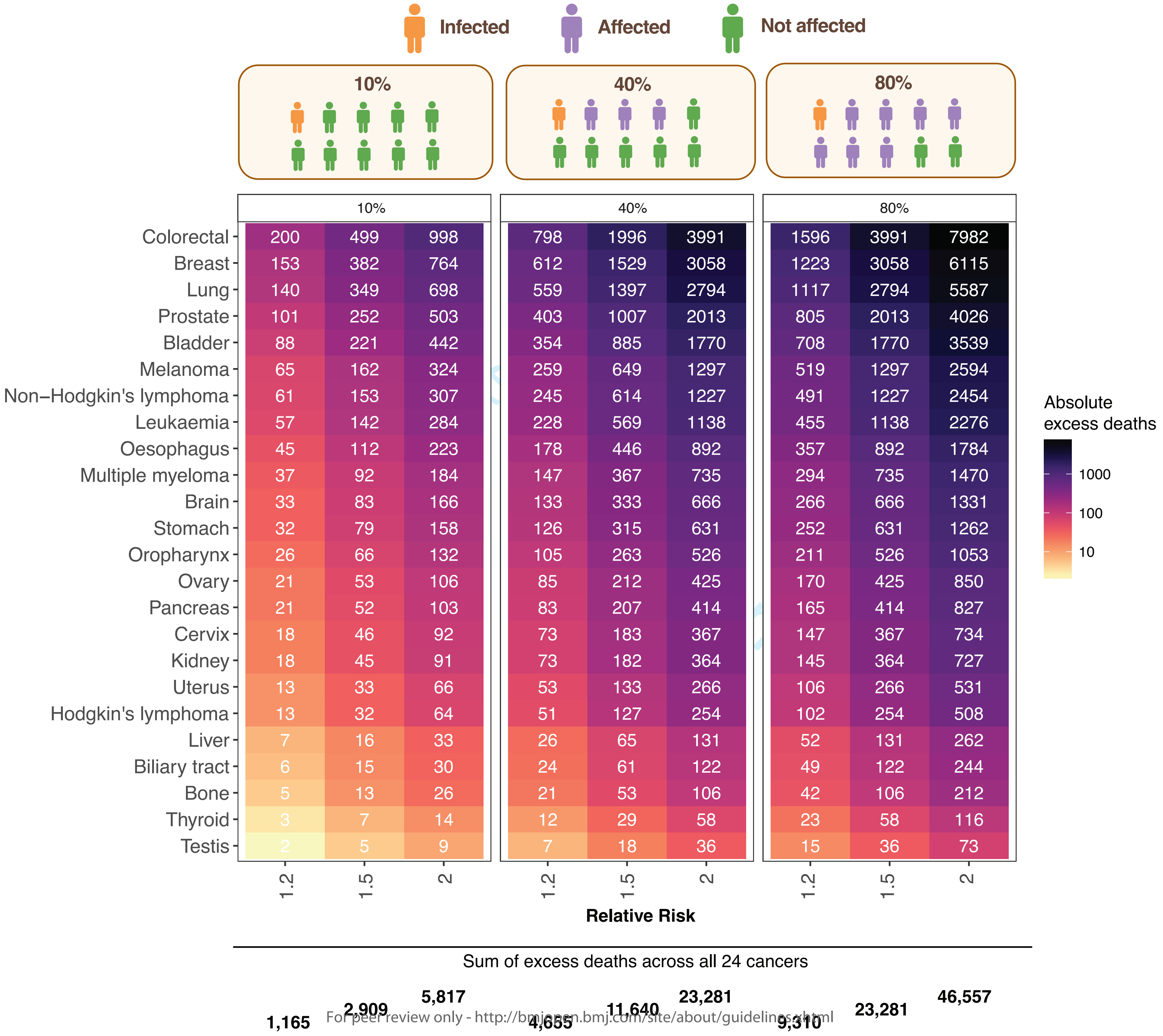

Figure S4. Proportion of patients with any of the 15 comorbidity clusters by cancer site for incident cancers (N=199,057) in a population of 3,862,012 adults in England. Age indicates mean age at diagnosis.

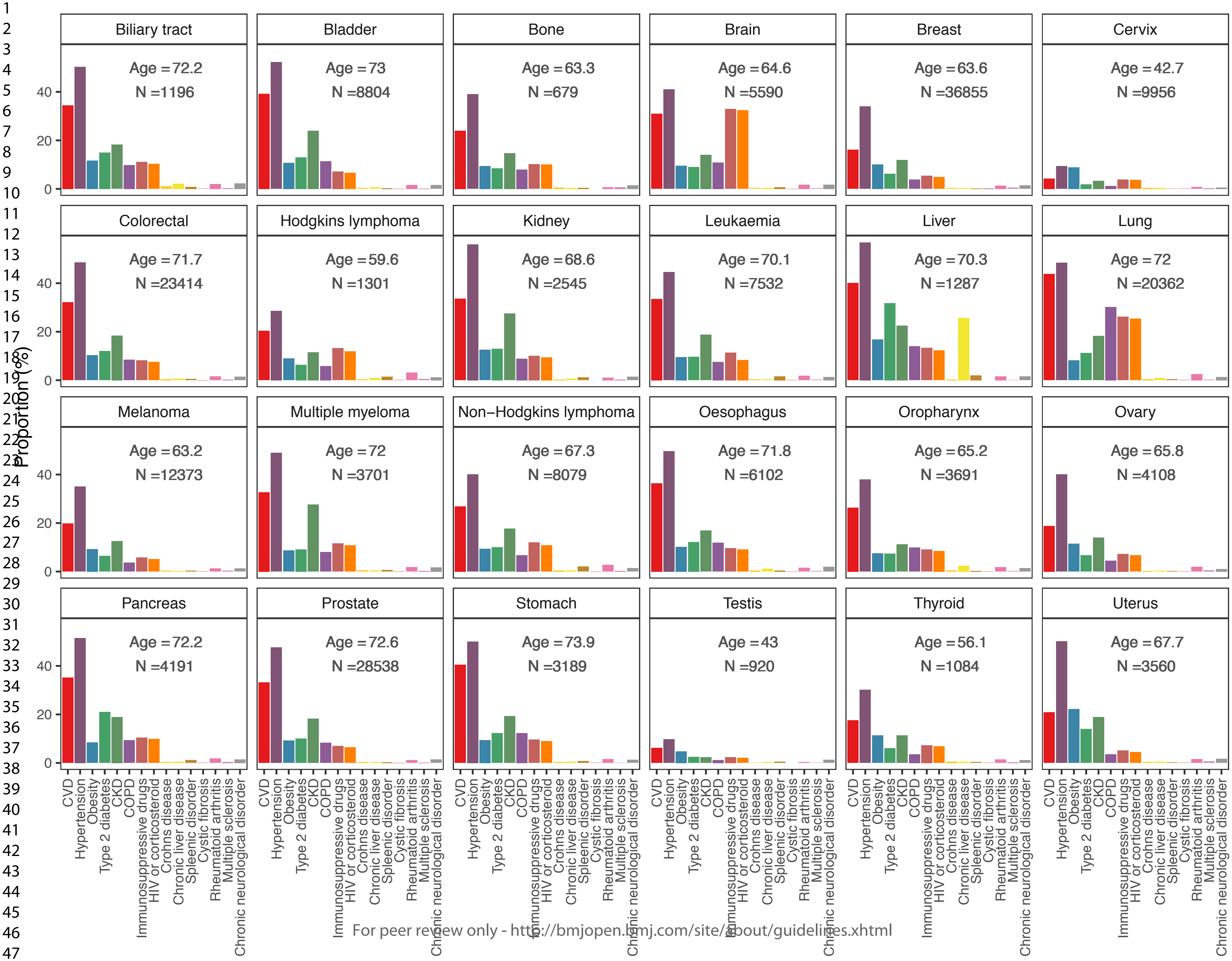

Figure S5. Proportion of patients with any of the 15 comorbidity clusters by cancer site for prevalent cancers (N=117,978) in a population of 3,862,012 adults in England. Age indicates mean age at diagnosis.

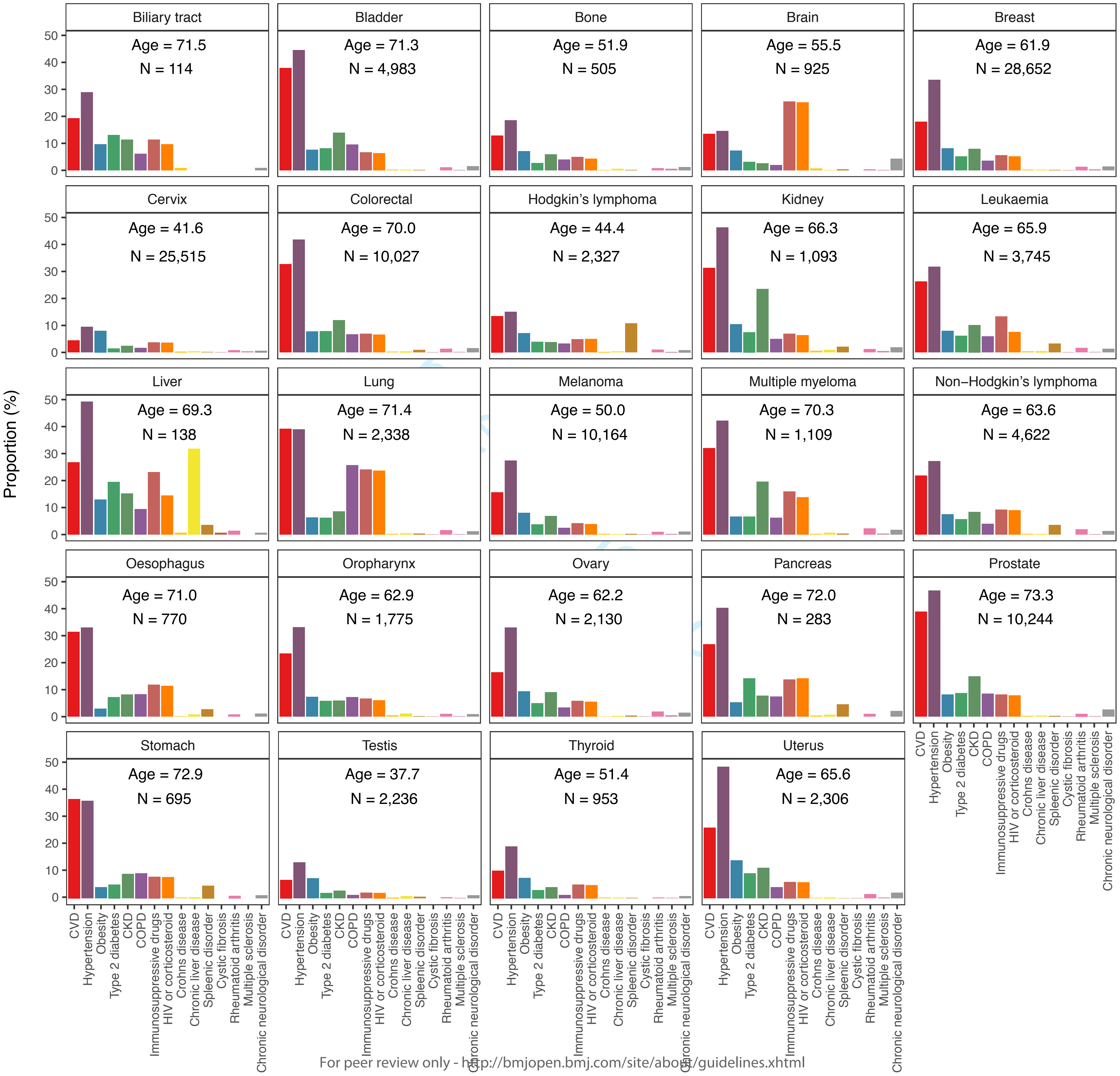

Figure S6. Forest plot of background (pre-COVID-19) 1-year cancer mortality for incident cases according to cancer site and number of underlying comorbidities in England. The whiskers are 95% confidence intervals.

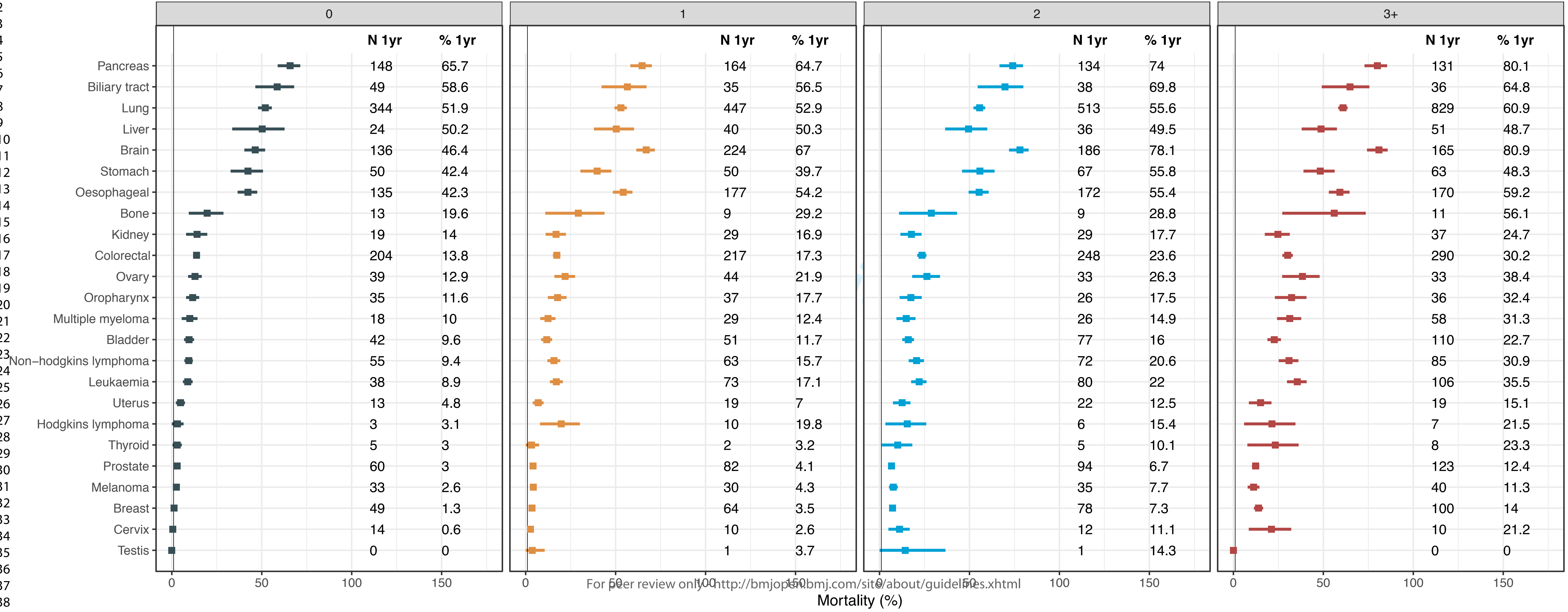

Figure S7. Forest plot of background (pre-COVID-19) 1-year cancer mortality for prevalent cases according to cancer site and number of underlying comorbidities in England. The whiskers are 95% confidence intervals.

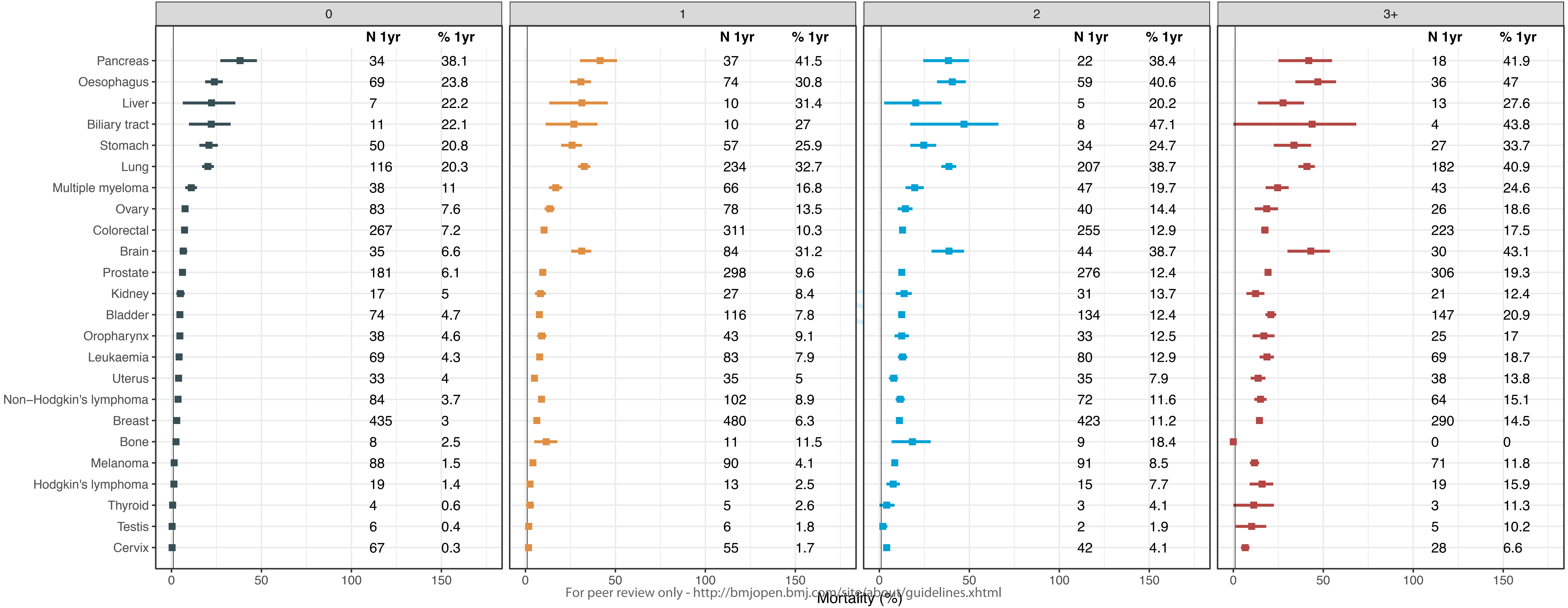

Figure S8. Total (direct and indirect) excess deaths for incident cancers by cancer site and number of comorbidities over a 1-year period. Stacked bar chart indicates the proportion of individuals with 0, 1, 2 and 3+ comorbidities by cancer site. We estimated total excess deaths for 40% (10% infected, 30% affected) of the population. Total excess deaths were scaled up to the population of England aged 30+ consisting of 35 million individuals using England mortality estimates for incident cancers.

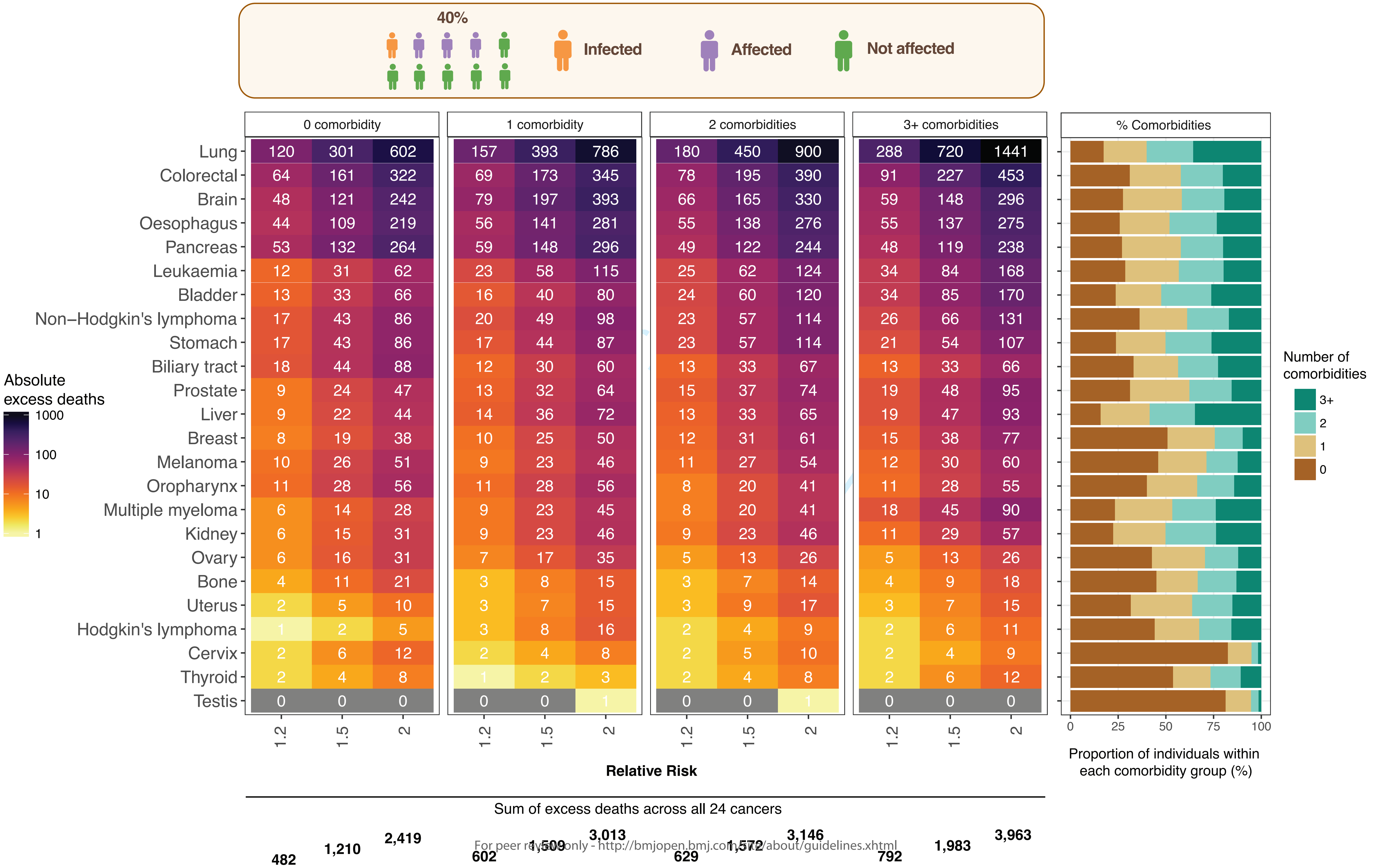

Figure S9. Total (direct and indirect) excess deaths for prevalent cancers by cancer site and number of comorbidities over a 1-year period. Stacked bar chart indicates the proportion of individuals with 0, 1, 2 and 3+ comorbidities by cancer site. We estimated total excess deaths for 40% (10% infected, 30% affected) of the population. Total excess deaths were scaled up to the population of England aged 30+ consisting of 35 million individuals using England mortality estimates for prevalent cancers.

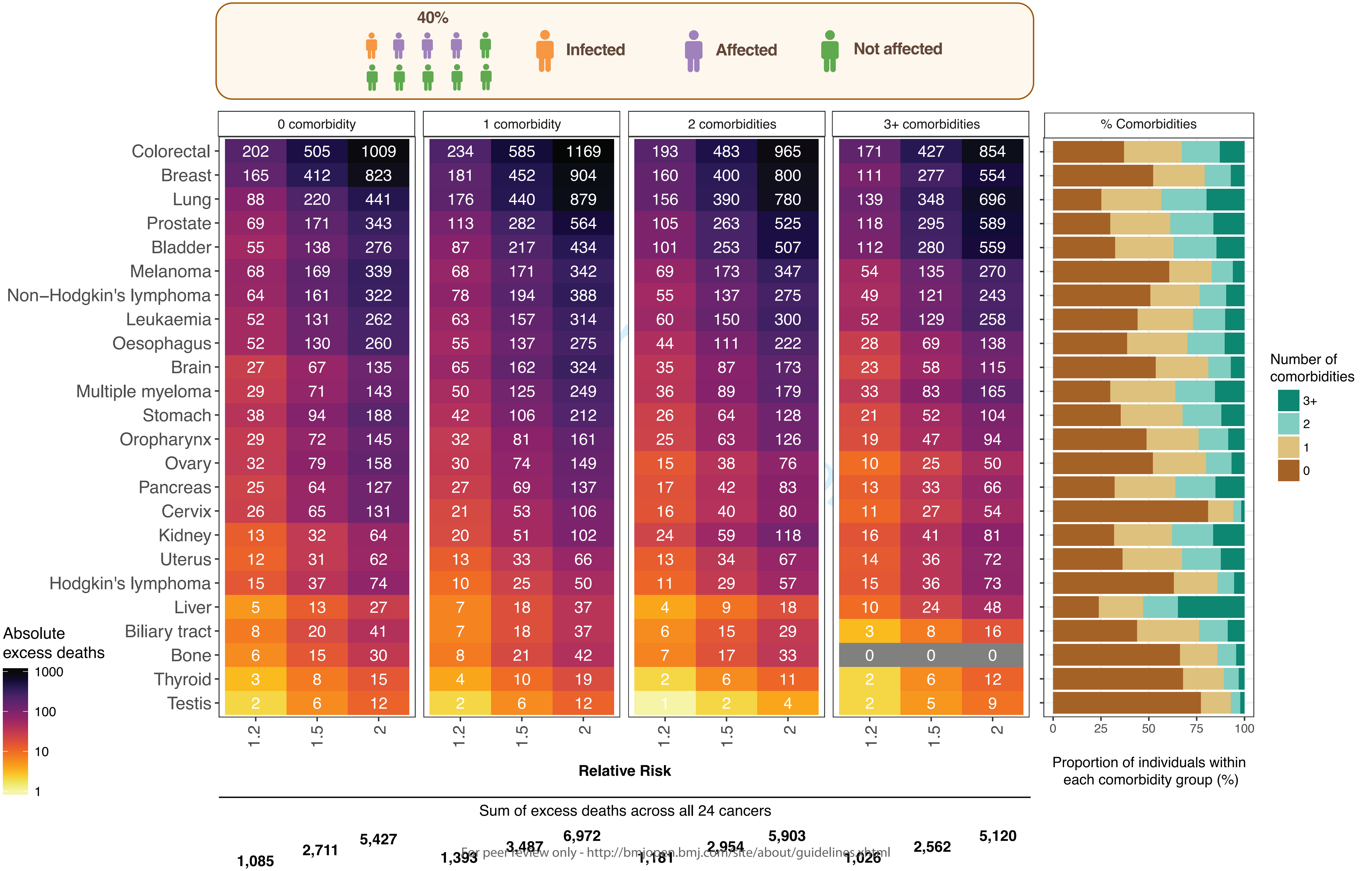

BMJ Open

Estimated impact of the Covid-19 pandemic on cancer services and excess 1-year mortality in people with cancer and multimorbidity: near-real-time data on cancer care, cancer deaths and a population-based cohort study

Journal:	*BMJ Open*
Manuscript ID	bmjopen-2020-043828.R1
Article Type:	Original research
Date Submitted by the Author:	20-Oct-2020

Complete List of Authors:	Lai, Alvina; University College London, Institute of Health Informatics<br>Pasea, Laura; The Farr Institute of Health Informatics Research, University College ,<br>Banerjee, Amitava; University College London, Farr Institute of Health Informatics Research<br>Hall, Geoff; St James's University Teaching Hospital,<br>Denaxas, S; University College London, Institute of Health Informatics<br>Chang, Wai Hoong; University College London, Institute of Health Informatics<br>Katsoulis, M; University College London, Institute of Health Informatics<br>Williams, Bryan; University College London, Institute of Cardiovascular Science<br>Pillay, Deenan; University College London, Medicine<br>Noursadeghi, Mahdad; University College London, 4Division of Infection & Immunity<br>Linch, David; University College London<br>Hughes, Derralynn; Royal Free Hospital and University College Medical School, Lysosomal Storage Disorders Unit, Department of Academic Haematology<br>Forster, Martin; University College London, Institute of Health Informatics<br>Turnbull, Clare; Institute of Cancer Research<br>Fitzpatrick, Natalie; UCL, Epidemiology and Public Health<br>Boyd, Kathryn; Northern Ireland Cancer Network<br>Foster, Graham; Barts and The London School of Medicine,<br>Enver, Tariq; University College London, Cancer Institute<br>Nafilyan, Vahe; Office for National Statistics<br>Humberstone, Ben; Office for National Statistics<br>Neal, Richard; University of Leeds, Leeds Institute of Health Sciences<br>Cooper, Matt; UCL Partners<br>Jones, Monica; University of Leeds<br>Pritchard-Jones, Kathy; University College London, UCL Great Ormond Street Institute of Child Health; University College London Hospitals NHS Foundation Trust, North Central London Cancer Alliance<br>Sullivan, Richard; Kings Health Partners<br>Davie, Charlie; UCL Partners<br>Lawler, Mark; Queen's University belfast, CCRCB<br>Hemingway, Harry; UCL, Epidemiology and Public Health

**Primary Subject Heading**:	Oncology
Secondary Subject Heading:	Epidemiology, Public health
Keywords:	COVID-19, ONCOLOGY, Health informatics < BIOTECHNOLOGY & BIOINFORMATICS

I, the Submitting Author has the right to grant and does grant on behalf of all authors of the Work (as defined in the below author licence), an exclusive licence and/or a non-exclusive licence for contributions from authors who are: i) UK Crown employees; ii) where BMJ has agreed a CC-BY licence shall apply, and/or iii) in accordance with the terms applicable for US Federal Government officers or employees acting as part of their official duties; on a worldwide, perpetual, irrevocable, royalty-free basis to BMJ Publishing Group Ltd ("BMJ") its licensees and where the relevant Journal is co-owned by BMJ to the co-owners of the Journal, to publish the Work in this journal and any other BMJ products and to exploit all rights, as set out in our licence.

The Submitting Author accepts and understands that any supply made under these terms is made by BMJ to the Submitting Author unless you are acting as an employee on behalf of your employer or a postgraduate student of an affiliated institution which is paying any applicable article publishing charge ("APC") for Open Access articles. Where the Submitting Author wishes to make the Work available on an Open Access basis (and intends to pay the relevant APC), the terms of reuse of such Open Access shall be governed by a Creative Commons licence – details of these licences and which Creative Commons licence will apply to this Work are set out in our licence referred to above.

Other than as permitted in any relevant BMJ Author's Self Archiving Policies, I confirm this Work has not been accepted for publication elsewhere, is not being considered for publication elsewhere and does not duplicate material already published. I confirm all authors consent to publication of this Work and authorise the granting of this licence.

For peer review only

Estimated impact of the Covid-19 pandemic on cancer services and excess 1-year mortality in people with cancer and multimorbidity: near-real-time data on cancer care, cancer deaths and a population-based cohort study

Alvina G. Lai§[1,2], Laura Pasea*[1,2], Amitava Banerjee*[1,2,3], Geoff Hall*[4,5,6], Spiros Denaxas[1,2,7,8], Wai Hoong Chang[1,2], Michail Katsoulis[1,2], Bryan Williams[7,9,10], Deenan Pillay[11], Mahdad Noursadeghi[11], David Linch[7,12], Derralynn Hughes[13,14], Martin D. Forster[9,13], Clare Turnbull[15], Natalie K. Fitzpatrick[1,2], Kathryn Boyd[16], Graham R. Foster[17], Tariq Enver[13], Vahe Nafilyan[18], Ben Humberstone[18], Richard D. Neal[19], Matt Cooper[4,5], Monica Jones[4,5], Kathy Pritchard-Jones[4,20,2122], Richard Sullivan[23], Charlie Davie[4,14,20], Mark Lawler§[4,24], Harry Hemingway§[1,2,6]

[1]Institute of Health Informatics, University College London, London, UK
[2]Health Data Research UK, University College London, London, UK
[3]Barts Health NHS Trust, The Royal London Hospital, Whitechapel Rd, London, UK
[4]DATA-CAN, Health Data Research UK hub for cancer hosted by UCLPartners, London, UK
[5]Leeds Institute of Medical Research, University of Leeds, Leeds, UK
[6]Leeds Teaching Hospitals NHS Trust, Leeds, UK
[7]University College London Hospitals NIHR Biomedical Research Centre, London, UK
[8]The Alan Turing Institute, London, UK
[9]University College London Hospitals NHS Trust, London, UK
[10]Institute of Cardiovascular Science, University College London, London, UK
[11]Division of Infection and Immunity, University College London, London, UK
[12]Department of Hematology, University College London Cancer Institute, London, UK
[13]University College London Cancer Institute, London, UK
[14]Royal Free NHS Foundation Trust, London, UK
[15]Division of Genetics and Epidemiology, Institute of Cancer Research, London, UK
[16]Northern Ireland Cancer Network, UK
[17]Barts Liver Centre, Blizard Institute, Queen Mary University of London, London, UK
[18]Office for National Statistics, London, UK
[19]Leeds Institute for Health Sciences, University of Leeds, Leeds, UK
[20]UCLPartners Academic Health Science Partnership, London UK
[21]Centre for Cancer Outcomes, University College London Hospitals NHS Foundation Trust, London, UK
[22]UCL Great Ormond Street Institute for Child Health, University College London, London, UK
[23]Conflict and Health Research Group, Institute of Cancer Policy, King's College London, London, UK
[24]Patrick G Johnston Centre for Cancer Research, Queen's University Belfast, UK

§ Joint senior authors
* Joint second authors

For correspondence: Alvina G. Lai (alvina.lai@ucl.ac.uk)

For peer review only - http://bmjopen.bmj.com/site/about/guidelines.xhtml

Abstract:

Objectives: To estimate the impact of the covid-19 pandemic on cancer care services and overall (direct and indirect) excess deaths in people with cancer.

Methods: We employed near real-time weekly data on cancer care to determine the adverse effect of the pandemic on cancer services. We also used these data, together with national death registrations until June 2020 to model deaths, in excess of background (pre-covid-19) mortality, in people with cancer. Background mortality risks for 24 cancers with and without covid-19-relevant comorbidities were obtained from population-based primary care cohort (Clinical Practice Research Datalink) on 3,862,012 adults in England.

Results: Declines in urgent referrals (median = -70.4%) and chemotherapy attendances (median = -41.5%) to a nadir (lowest point) in the pandemic were observed. By 31st May, these declines have only partially recovered; urgent referrals (median = -44.5%) and chemotherapy attendances (median = -31.2%). There were short-term excess death registrations for cancer (without covid-19), with peak relative risk (RR) of 1.17 at week ending 3rd April. The peak RR for all-cause deaths was 2.1 from week ending 17th April. Based on these findings and recent literature, we modelled 40% and 80% of cancer patients being affected long-term by the pandemic. At 40% affected, we estimated 1-year total (direct and indirect) excess deaths in people with cancer as between 7,165 and 17,910, using RR of 1.2 and 1.5 respectively, where 78% of excess deaths occur in patients with ≥ 1 comorbidity.

Conclusions: Dramatic reductions were detected in the demand for, and supply of, cancer services which have not fully recovered with lockdown easing. These may contribute, over a 1-year time horizon, to substantial excess mortality among people with cancer and multimorbidity. It is urgent to understand how the recovery of general practitioner, oncology and other hospital services might best mitigate these long-term excess mortality risks.

For peer review only

Strengths and limitations of this study

- This is the first study that used hospital data and a predictive model to dissect and quantify the adverse impact on mortality of the pandemic on patients with cancer and multimorbidity.
- This study used the breadth of longitudinal information in primary care records from the Clinical Practice Research Datalink to generate background (pre-COVID-19) mortality estimates for patients with cancer.
- This study generated 1-year mortality estimates for 24 cancer types and evaluated the extent by which multimorbidity influences mortality risk in patients with cancer. We considered 15 comorbidity clusters, which include 40 non-malignant comorbidities defined by the Public Health England as associated with severe and fatal covid-19 infection.
- This study modelled excess deaths using information on background mortality risk and plausible relative risk estimates obtained from the Office for National Statistics and other published studies.
- A limitation of this study is the use of primary care health records which may have missed cases of cancer resulting in more conservative estimations of excess deaths.

For peer review only - http://bmjopen.bmj.com/site/about/guidelines.xhtml

Introduction:

The covid-19 pandemic may cause additional (excess) deaths due both to the direct effects of infection and the indirect effects that result from the repurposing of health services designed to address the pandemic[1]. People with cancer are at increased risk of contracting and dying from SARS-CoV-2 infection[2,3]. Optimal cancer care must balance protecting patients from SARS-CoV-2 infection, with the need for continued access to early diagnosis and delivery of optimal treatment[4,5]. Professional cancer associations internationally have recommended reducing systemic anti-cancer treatment, surgery and risk-adapted radiotherapy[6]. In June 2020, the NHS released statistics for April 2020, indicating that referrals to a consultant for urgent diagnosis of cancer had fallen by 60%[7]. Some cancer surgical procedures have been postponed and cancer screening programmes paused[8–13].

However, covid-19-induced healthcare service reconfiguration and recovery have, to date, not been informed by near real-time hospital data quantifying the extent of disruption for cancer patients resulting from this service reconfiguration, nor its impact on excess deaths in people with cancer. A previous study has employed literature-based estimates to model the impact of potential diagnostic delays in colorectal cancer during the covid-19 pandemic[14,15]. Short-term (30 days) death in people with cancer and covid-19 is importantly driven by (treatable) comorbidities such as hypertension and cardiovascular disease[16]. Public Health England (PHE) have identified patients with these and a wide range of other non-malignant conditions as at greater risk of developing severe illness from SARS-CoV-2 exposure[17,18], while multimorbidity in cancer is an increasing clinical concern[19,20]. For general practitioners and oncologists, evidence is required on the pan-cancer estimation of mortality risks according to type and number of comorbid conditions. Such evidence may inform individual decisions about physical isolation and shielding, as well as the need to ensure that patients access specialist cancer care and seek preventive care for non-malignant comorbidities.

Our objectives were: (i) to quantify changes in cancer care, reporting near-real-time weekly data (to June 2020) for urgent referral (for early diagnosis of cancer) and chemotherapy attendance (for treatment of cancer); (ii) to quantify short-term direct and indirect excess deaths using near real-time weekly death registrations from the Office for National Statistics (ONS); (iii) to estimate the number of annual direct (covid-19) and indirect excess deaths using population-based 1-year Kaplan-Meier mortality estimates for 24 cancer types and (iv) to determine the extent by which multimorbidity contributes to these excess deaths.

For peer review only

For peer review only - http://bmjopen.bmj.com/site/about/guidelines.xhtml

Methods:

Weekly near real-time hospital data

To estimate the extent to which changes in cancer services during different phases of the pandemic (pre-lockdown, lockdown, post lockdown easing) have impacted on cancer care delivery, we sought weekly information for urgent cancer referrals for early diagnosis ('two-week-wait' [2WW]), an indicator of both patient demand and health service supply i.e., how well the service is ensuring that individuals with suspicious symptoms are rapidly prioritised to the diagnostic cancer pathway) and chemotherapy attendances (an indicator of supply and a proxy for possible adverse effects of the pandemic on the cancer treatment pathway). We employed the UK's Health Data Research Hub for Cancer (DATA-CAN) [21] to approach eight hospital trusts (in Leeds, London and Northern Ireland) and sought data from January 2019 to June 2020 to control for seasonal changes. Each hospital trust rapidly provided the requested data and permission to share these data in the public domain. We estimated the % change in weekly activity compared to the mean activity in 2019.

Weekly near real-time death registration data

To estimate direct (among those infected) and indirect impact of the covid-19 pandemic on deaths, we sought weekly counts of deaths in England and Wales from the Office for National Statistics (ONS), with causes classified by the ONS as covid-19 deaths, non-covid-19 deaths excluding cancer and cancer deaths.

Study population: primary care population-based cohort

To estimate pre-covid-19 incidence and mortality in individuals with cancer, we used population-based electronic health records in England from primary care data from the Clinical Practice Research Datalink (CPRD) linked to the ONS death registration. We used this primary care data source because of the extensive information on comorbidities (which may be lacking in cancer registry data). The study population was 3,862,012 adults aged ≥ 30 years, registered with a general practice from 1 January 1997 to 1 January 2017, with at least 1 year of follow-up data. CPRD data are representative of the English population in terms of age, sex, mortality and ethnicity[22–24], with extensive evidence of validity[25]. This study was performed as part of the CALIBER programme (https://www.ucl.ac.uk/health-informatics/caliber). CALIBER is an open-access research resource consisting of information, tools and phenotyping algorithms available through the CALIBER Portal (https://caliberresearch.org/portal)[26,27]. The study was approved by the MHRA (UK) Independent Scientific Advisory Committee (20_074R2), under Section 251 (NHS Social Care Act 2006).

Open-access definitions of disease using electronic health records

We defined non-fatal incident cases (as alive for at least 30 days following cancer diagnosis) and prevalent cases of cancer across 24 primary cancer sites according to previously validated CALIBER

For peer review only - http://bmjopen.bmj.com/site/about/guidelines.xhtml

electronic health record phenotypes. Incident cancers were defined as new cancer diagnoses after the study entry into CPRD (baseline). Prevalent cancers were defined as cancer diagnoses recorded at any time prior to baseline. The cancers included: biliary tract, bladder, bone, brain, breast, cervix, colorectal, Hodgkin's lymphoma, kidney, leukaemia, liver, lung, melanoma, multiple myeloma, non-Hodgkin's lymphoma, oesophagus, oropharynx, ovary, pancreas, prostate, stomach, testis, thyroid and uterus[28]. Phenotype definitions of cancers and covid-19-relevant comorbidities are available at https://caliberresearch.org/portal and have previously been validated[29–32]. Phenotypes were generated from hospital and primary care information recorded in primary care, using Read clinical terminology (version 2).

Comorbidities relevant to covid-19

We examined 15 comorbidity clusters, which include 40 non-malignant comorbidities defined by PHE as associated with severe and fatal covid-19 infection[17,18]. We separately estimated the proportion of patients with each comorbidity at study entry (prevalent cancers), and at the date of the first diagnosis of incident cancer. The PHE list included chronic respiratory disease, chronic heart disease, immunocompromised individuals, HIV, use of corticosteroids, obesity, diabetes, chronic kidney disease, chronic liver disease, chronic neurological disorders and splenic disorders. A full list of the conditions we examined, and their definitions is provided in Supplementary Methods.

Estimating incidence rates and 1-year mortality

We estimated incidence rates per 100,000 person-years and 1-year mortality in our study population. Estimated incidence rates for the number of new cancers by cancer site were compared with those for the UK from the International Agency for Research on Cancer (IARC) and were found to be representative. We estimated baseline 1-year mortality risk following cancer diagnosis for both incident and prevalent cancers using Kaplan-Meier analyses stratified by cancer sites and number of (non-cancer) comorbid conditions (0, 1, 2 and 3+). We used the most recent 5 years of data (2012-2016) to estimate 1-year mortality.

Estimating 1-year direct excess deaths

Excess deaths were estimated by applying relative risks (RRs) to the background 1-year mortality risk. Direct excess deaths (due to or with covid-19) were modelled using the range of relative risks (1.2, 1.5 and 2.0) previously reported in studies of cancer and covid-19 deaths.[3,33] We applied these RRs to 10% of the population (the directly "infected"), based on recent SARS CoV-2 seroprevalence estimates in the UK[34,35] and other countries[36,37]. Although the infection rate will change depending on the phase of the pandemic, we assumed an infection rate over 1 year in line with the first wave of the pandemic.

For peer review only - http://bmjopen.bmj.com/site/about/guidelines.xhtml

Estimating 1-year total (direct and indirect) excess deaths

Indirect excess deaths (due to pandemic-induced health service reconfiguration) were estimated by applying RRs for excess cancer deaths observed using ONS data, by taking the number of weekly cancer deaths from January 2020 divided by the weekly average over the last 5 years. We assumed that the effects of service change may not translate to an immediate increase in excess deaths. We have applied the RR of 1.2, 1.5 and 2 to 40% (10% infected, 30% affected) and 80% (10% infected, 70% affected) of the population and modelled excess deaths over a 12-month period to capture medium term effects. We chose this range of indirectly "affected" population based on our real-time estimates of the degree of perturbation in cancer care during the pandemic and patient reports that clinical care had been cancelled during the pandemic for 53%-70% of patients with cancer or other conditions[38].

To project the study estimates of excess deaths to the whole English population, we employed the 2018 population estimate, where the number of deaths is scaled up to a population of 35,407,313 individuals aged 30 and above[39]. All analyses were performed using R (version 3.4.3).

For peer review only

Results

Near real-time data on cancer care

Evaluating data from 291,792 people with suspected cancer and 150,636 patients with cancer attending for chemotherapy from January 2019 to June 2020, we initially characterised the pre-pandemic basal level of activity (2019 average), including seasonal variations (Figure 1). Using the date of the 50th patient diagnosed with covid-19 as the starting point of the pandemic, we observed that urgent referrals fell by 70.4% (range: -68.7% to -84.3%), while chemotherapy attendances declined by 41.5% (range: -26.3% to -63.4%) (Figure 1). To highlight these adverse impacts, we provided these data to Chief Medical Officers in all 4 nations of the United Kingdom and the National Director for Cancer (England). We have also continued to provide regular updates of this intelligence to the Scientific Advisory Group for Emergencies. Since the NHS letter on 29 April 2020 re-starting cancer and other services[40], and since easing of lockdown (11 May 2020), there has been evidence of recovery for the urgent two-week-wait referrals (-55.4% to -40.0%; median = -44.5%), and chemotherapy attendances (-37.1% to 3.9%; median = -31.2%) (Figure 1).

Near real-time data on cancer, covid-19 and other deaths

There were 1,307 excess cancer deaths from 13th March to 15th May 2020 compared to the 5-year average based on weekly registration of deaths for England and Wales (Figure 2A). We found an excess in cancer deaths with a peak in the week ending 3rd April 2020 with a relative risk (RR) of 1.17 (Figure 2B). There were 41,105 covid-19 deaths up until 15th May 2020. For non-covid-19 deaths (excluding cancers), we found that the peak occurred with a RR of 1.37 on 24th April 2020. The peak RR for all-cause deaths was 2.1, from week of 17th April 2020.

Estimations on direct excess deaths by cancer site over 1-year

We estimated direct excess covid-19 deaths based on a SARS CoV-2 infection rate of 10% and background 1-year mortality risks (Figure 3A). For both incident and prevalent cancers combined, we estimated 1,790, 4,479 and 8,957 direct excess deaths at RR of 1.2, 1.5 and 2.0 respectively (Figure 3B). Incidence rates for 24 cancer types were shown in Figure S1. Figures S2 and S3 show the separate direct excess death estimates for incident and prevalent cancers.

Estimations of total (direct and indirect) excess deaths by cancer site over a 1-year

When applying RRs of 1.2 or 1.5 to 40% (10% infected, 30% affected) of the population of people with cancer (both incident and prevalent cancers), we estimated 7,165 and 17,910 total excess deaths respectively (Figure 3B). When applying these RRs to 80% (10% infected, 70% affected) of the population of people with cancer, we estimated 14,326 and 35,817 total excess deaths respectively (Figure 3B). Figures S2 and S3 show the separate total excess death estimates for incident and prevalent cancers.

For peer review only - http://bmjopen.bmj.com/site/about/guidelines.xhtml

Comorbidities relevant to covid-19 risk: prevalence and association with 1-year mortality

Comorbidities were common in people with incident cancer: hypertension (83,313 [41.9%]), cardiovascular disease (55,742 [28.0%]), chronic kidney disease (31,935 [16.0%]), obesity (19,589 [9.8%]), type 2 diabetes (18,957 [9.5%]) and COPD (18,373 [9.2%]) (Figure S4). Similar patterns were seen in prevalent cancers (Figure S5). Multimorbidity (≥1 comorbidity) was associated with a higher 1-year mortality (Figure S6 for incident cancers; Figure S7 for prevalent cancers). For example, for incident colorectal cancer, 1-year mortality for 0, 1, 2 and 3+ comorbidities, was 13.8%, 17.3%, 23.6% and 30.2% respectively (Figure S6).

Estimations of total (direct and indirect) excess deaths by cancer site and number of comorbidities over a 1-year period

To ascertain the influence of multimorbidity on total excess deaths, we provide estimates based on 40% (10% infected, 30% affected). For both incident and prevalent cancers, 78% of the predicted excess deaths occur in people with 1+ comorbidity. For example, at RR of 1.2, there are 5,622 excess deaths in those with 1+comorbidity compared to 1,567 in those with no comorbidities (total 7,189) (Figure 4). Even though the size of patient group in 0, 1, 2 and 3+ comorbidities declines (49.8%, 24.7%, 15.0% and 10.6% respectively) the absolute numbers of excess deaths in each comorbidity group are similar, suggesting that patients with comorbidities contribute to a large proportion of excess deaths compared to those without non-cancer comorbidities. For example, at RR of 1.5, the numbers of total excess deaths for both incident and prevalent cancers were 3,922, 4,993, 4,526 and 4,542 in individuals with 0, 1, 2 and 3+ non-cancer comorbidities respectively (Figure 4). The findings for incident and prevalent cases are presented separately in Figure S8 and Figure S9.

We share the underlying study estimates from this paper (online data supplement) and provide an open-access tool for researchers to interact with the model (https://pasea.shinyapps.io/cancer_covid_app/).

For peer review only - http://bmjopen.bmj.com/site/about/guidelines.xhtml

Discussion:

Statement of principal findings

To our knowledge, this is the first study with near real–time evidence of covid-19's negative impact on cancer services at different phases of the pandemic, its potential to lead to significant excess deaths in people with cancer and the substantial role that comorbidities may play in these excess deaths.

<u>*Changes in cancer care at different phases of pandemic:*</u> We delineate both the nadir and the incomplete recovery of UK cancer services that have resulted from the covid-19 pandemic. We observed profound declines in urgent two-week wait (2WW) referrals for early cancer diagnosis, which have not returned to pre-covid-19 levels. These may reflect patients' deciding not to seek care due to the perceived risk of infection, but may also be in part due to difficulty in securing appointments due to reprioritised health systems[19]. An unintended consequence of this reprioritisation may be excess deaths due to delayed diagnoses, increased emergency presentations, more advanced stage at presentation, and changes in care pathways that adversely affect outcomes. We also observed large declines in chemotherapy attendance, presumably reflecting capacity/resources being redirected to care for infected patients (e.g., to intensive care) and the desire of clinicians and patients to minimise the risks of covid-19 for susceptible cancer patients [10].

<u>*Direct (covid-19) excess deaths:*</u> It is important to note that our model estimates deaths additional to those that would be expected (without covid-19) in people with cancer. At a RR of 2, we estimate about 9,000 direct covid-19 excess deaths in 1 year in people with cancer but acknowledge there is uncertainty in this estimate. There is increasing concern that those discharged from hospital with covid-19 may have long term, (including fatal) sequelae.

<u>*Total (direct and indirect) excess deaths:*</u> Based on our observations regarding the adverse effects of cancer service reprioritization, we consider a proportion affected by the pandemic of 40% plausible, if perhaps somewhat conservative. But, given that adverse effects could be more profound (our 2WW referrals data, for example, would suggest this), we present excess deaths for a range of both 40% and 80%. Adding credibility to our estimates, in a survey in April 2020 of 17,000 UK adults, 56% of cancer patients reported that the NHS had cancelled their treatment[38] Overall, we conservatively estimate, at RR of 1.5, that 17,910 total excess deaths for 1 year will occur in patients with cancer, but this could rise to 35,817. We note the degree of uncertainty in the observed RR at different points in the pandemic. Patients affected by changes in cancer services in March–June 2020 may not necessarily directly contribute to an increase in excess indirect deaths in these four months, as the effects on health and mortality outcomes are more likely to occur in a longer time frame.

For peer review only - http://bmjopen.bmj.com/site/about/guidelines.xhtml

Importance of multimorbidity: We demonstrate that the majority (78%) of excess deaths in people with cancer during the covid-19 pandemic occur in people with at least 1 comorbidity. While many of these comorbidities are treatable, services for these conditions have also been affected by the pandemic. For example, 65% of patients with hypertension and 70% of patients with diabetes reported that the NHS had recently cancelled their care, as captured in the same April 2020 survey noted above[38]. Importantly, the pandemic prompts new questions about which cancer patients are most vulnerable and how best to mitigate an individual's personal risk.

Strengths of this study

There are three major strengths of this study. First, the acquisition and deployment of near real-time data to signal the significant adverse impact of the covid-19 pandemic on cancer services and how this has profound implications for cancer diagnostic and treatment pathways. These data were also used to inform and enhance our existing model that estimates excess mortality due to the pandemic. Second, we provide a pan-cancer comorbidity atlas using a population-based 3.8million primary care cohort to underpin estimates of the additional adverse effect of multimorbidity in cancer patients; cancer registry data tend to lack this more comprehensive information. Third, we provide separate estimates of excess deaths for prevalent cancers and incident (newly diagnosed) cancers, because these represent different patterns of risk, treatment priorities, and roles of general practitioner and oncologist.

Weaknesses of this study

Our model has important limitations. First, there is a lack in the literature of studies on clinical cohorts of cancer patients investigating all-cause mortality rates in those with and without infection; such studies are needed in order to obtain better estimates of the direct effects of the pandemic. Second, the primary care health records we used may have missed some cases of cancer and thus underestimated incidence[41]. If so, our estimates of excess deaths may be conservative. The NHS has national linked hospital admissions and cancer registration data with information on stage and details of surgical, chemotherapeutic and radiotherapy treatment of cancer. However, information governance for such data can take months to secure, making data-enabled research and time-sensitive responsive service improvement difficult. Third, we did not have access to data on children. Fourth, we only have access to empirical cancer service change data from eight hospitals in the UK. Whilst the data may be a representative sample of the UK population, and patterns of decline in service change is corroborated in another study[42], more widespread access to other trusts may be beneficial to ascertain national and regional effects.

Implications for clinicians and policymakers

For peer review only - http://bmjopen.bmj.com/site/about/guidelines.xhtml

Our study may inform decision-making at three levels. First, from a healthcare policy and healthcare implementation perspective, it is clear that the NHS cannot simply be ‘switched on’ again at full capacity for hospital or primary care services as there will be a significant backlog of untreated patients, with waiting lists predicted to expand to 10 million patients. Data published on June 13th 2020 indicate ~100,000 “missing” cancer referrals in April 2020 alone[7]. More granular weekly intelligence from the centres contributing data to this study suggests that this negative impact will continue for at least six to nine months, placing many more patients at risk.

Second, there are currently no accessible national systems available for near real-time data on care and outcomes of cancer patients. Our study suggests that we should expand our near real-time data approach across the UK to collect actionable information on (i) death certification – in particular distinguishing the contribution of cancer, comorbid conditions and covid-19 to death; (ii) cancer health services activity data, to monitor how changes at each phase of the pandemic (including clearing backlogs for under-referral, under-diagnosis and under-treatment) might influence future health outcomes and (iii) treatment services data for non-malignant comorbidities of cancer patients, such as cardiovascular disease, diabetes and hypertension.

Third, with knowledge of mortality risk based on type of cancer, age and comorbidities that we provide in an online format (https://pasea.shinyapps.io/cancer_covid_app/), supplemented with local knowledge of health service resilience, we propose that weekly indicators and warnings for vulnerable cancer patients with multimorbidity could be provided. Using this intelligence, treatment prioritization as we resume cancer services could be enhanced by patient-specific risk/benefit assessments which include multimorbidity, particularly in situations where treatment provision outweighs non-treatment/safety issues related to covid-19[19].

Unanswered questions and future research

There are important areas for further research. First, there is a need for long-term (1 to 5 years) monitoring of the extent to which cancer patients experience excess mortality due to the pandemic. We chose a 1-year time horizon, because the adverse consequences on health are likely to extend beyond the initial wave of the pandemic. But its impact on excess mortality in patients with cancer, particularly those whose diagnosis/treatment is delayed, may take years to understand. The specific impact of paused cancer screening, particularly for breast and colorectal cancer may be profound. The social and psychological consequences of physical distancing on mortality may also be particularly important in cancer[43,44], while international studies across 75 countries signpost how unemployment negatively impacts mortality in cancer patients[45]. Hence, the socio-economic effects of the current pandemic are likely to last for a considerable period beyond one year[46]. As new empirical data become available on heath service, social/psychological and economic changes,

For peer review only - http://bmjopen.bmj.com/site/about/guidelines.xhtml

our model can better specify the proportion and type of cancer patients thus affected and look to develop appropriate mitigation strategies.

Conclusion

We mobilised usually inaccessible near real-time hospital data to quantify the immediate adverse impacts of the covid-19 pandemic on cancer services, on people who may demonstrate symptoms of cancer and on patients who are being treated for cancer. The marked reductions observed in the demand for, and supply of, cancer services have only partially recovered with lockdown easing. Such perturbations in cancer care may contribute, over a 1-year time horizon, to substantial excess mortality among people with cancer and multimorbidity. There is an urgent need to better understand and mitigate these excess mortality risks, some of which may be revealed only over the longer term.

For peer review only

For peer review only - http://bmjopen.bmj.com/site/about/guidelines.xhtml

Contributorship statement:

Research question: AGL, HH

Funding: AGL, AB, ML, HH

Study design and analysis plan: AGL, LP, AB, MK, WHC, HH

Preparation of data, including electronic health record phenotyping in the CALIBER portal: AGL, LP, SD

Provision of weekly hospital data: GH, KPJ, MDF, DH, ML, KB, CD

Statistical analysis: AGL, LP, WHC, MK

Drafting initial versions of manuscript: AGL, ML, HH

Drafting final versions of manuscript: AGL, GH, CD, ML, HH,

Critical review of early and final versions of manuscript: AGL, LP, AB, GH, SD, WHC, MK, BW, DP, MN, DL, DH, MDF, CT, NKF, KB, GRF, TE, VN, BH, RDN, MC, MJ, KPJ, RS, CD, ML, HH

Declaration of interests:

ML has received honoraria from Pfizer, EMD Serono and Roche for presentations unrelated to this research. ML has received an unrestricted educational grant from Pfizer for research unrelated to the research presented in this paper. AB has received research funding from AstraZeneca. MDF has received research funding from AstraZeneca, Boehringer Ingelheim, Merck and MSD and honoraria from Achilles, AstraZeneca, Bayer, Boehringer Ingelheim, Bristol-Meyers Squibb, Celgene, Guardant Health, Merck, MSD, Nanobiotix, Novartis, Pharmamar, Roche and Takeda for advisory roles or presentations unrelated to this research. GRF receives funding from companies that manufacture drugs for hepatitis C virus (AbbVie, Gilead, MSD) and consult for GSK, Arbutus and Shionogi in areas unrelated to this research.

Acknowledgments:

We thank Tony Hagger, Shiva Thapa, Mohammed Emran, Cara Anderson, Louise Herron, Joy Beaumont, Maurice Loughrey, Philip Melling and Lee Cogger for their help on collating data on urgent cancer referrals and chemotherapy attendances. We thank the HDR UK DATA-CAN Patient and Public Involvement and Engagement panel for critical feedback on the manuscript. We thank Charles Swanton for his valuable comments on the manuscript. This study is based in part on data from the Clinical Practice Research Datalink obtained under licence from the UK Medicines and Healthcare products Regulatory Agency. The data is provided by patients and collected by the NHS as part of their care and support. Mortality data are from the Office for National Statistics (ONS).

Funding statement:

We acknowledge Health Data Research UK (HDR UK) support for the HDR UK substantive sites involved in this research (HDR London, HDR Wales and Northern Ireland) and DATA-CAN. DATA-CAN (MC_PC_19006) is part of the Digital Innovation Hub Programme, delivered by HDR UK and

For peer review only - http://bmjopen.bmj.com/site/about/guidelines.xhtml

funded by UK Research and Innovation through the government's Industrial Strategy Challenge Fund. AGL is supported by funding from the Wellcome Trust (204841/Z/16/Z), National Institute for Health Research (NIHR) University College London Hospitals Biomedical Research Centre (BRC714/HI/RW/101440), NIHR Great Ormond Street Hospital Biomedical Research Centre (19RX02) and the Health Data Research UK Better Care Catalyst Award. AB is supported by research funding from NIHR, British Medical Association, Astra-Zeneca and UK Research and Innovation. KPJ is supported by the NIHR Great Ormond Street Hospital Biomedical Research Centre. CD and KPJ is funded by UCLPartners. HH is an NIHR Senior Investigator and is funded by the NIHR University College London Hospitals Biomedical Research Centre, supported by Health Data Research UK (grant No. LOND1), which is funded by the UK Medical Research Council, Engineering and Physical Sciences Research Council, Economic and Social Research Council, Department of Health and Social Care (England), Chief Scientist Office of the Scottish Government Health and Social Care Directorates, Health and Social Care Research and Development Division (Welsh Government), Public Health Agency (Northern Ireland), British Heart Foundation, Wellcome Trust, The BigData@Heart Consortium, funded by the Innovative Medicines Initiative-2 Joint Undertaking under grant agreement No. 116074.

Data sharing statement:

Data used in this study was accessed through the Clinical Practice Research Datalink that is subject to protocol approval by an Independent Scientific Advisory Committee and cannot directly be shared. All results are reported in the manuscript and no additional data is available.

Patient and public involvement statement:

The Health Data Research UK hub for cancer (DATA-CAN) patient and public advisory panel were consulted during the writing of this manuscript.

Ethics approval:

The study was approved by the MHRA (UK) Independent Scientific Advisory Committee (20_074R2), under Section 251 (NHS Social Care Act 2006).

For peer review only

For peer review only - http://bmjopen.bmj.com/site/about/guidelines.xhtml

References:

1 Banerjee A, Pasea L, Harris S, *et al.* Estimating excess 1-year mortality associated with the COVID-19 pandemic according to underlying conditions and age: a population-based cohort study. *Lancet* 2020;**395**:1715–25. doi:10.1016/S0140-6736(20)30854-0

2 Kuderer NM, Choueiri TK, Shah DP, *et al.* Clinical impact of COVID-19 on patients with cancer (CCC19): a cohort study. *Lancet* 2020.

3 Williamson E, Walker AJ, Bhaskaran KJ, *et al.* OpenSAFELY: factors associated with COVID-19-related hospital death in the linked electronic health records of 17 million adult NHS patients. *MedRxiv* 2020.

4 Rosenbaum L. The Untold Toll — The Pandemic's Effects on Patients without Covid-19. *N Engl J Med* doi:10.1056/NEJMms2009984

5 Vrdoljak E, Sullivan R, Lawler M. Cancer and COVID-19; how do we manage cancer optimally through a public health crisis? *Eur J Cancer* 2020;**132**:P98-99.

6 Thomson DJ, Palma D, Guckenberger M, *et al.* Practice recommendations for risk-adapted head and neck cancer radiotherapy during the COVID-19 pandemic: an ASTRO-ESTRO consensus statement. *Int J Radiat Oncol Biol Phys* 2020.

7 NHS England. Provider-based Cancer Waiting Times for April 2019. Accessed 17 June 2020. https://www.england.nhs.uk/statistics/statistical-work-areas/cancer-waiting-times/monthly-prov-cwt/2019-20-monthly-provider-cancer-waiting-times-statistics/provider-based-cancer-waiting-times-for-april-2019-provisional/

8 Burki TK. Cancer guidelines during the COVID-19 pandemic. *Lancet Oncol* 2020.

9 British Society of Gastroenterology. Endoscopy activity and COVID-19: BSG and JAG guidance. Accessed 19 April 2020. https://www.bsg.org.uk/covid-19-advice/endoscopy-activity-and-covid-19-bsg-and-jag-guidance/

10 National Institute for Health and Care Excellence. COVID-19 rapid guideline: delivery of systemic anticancer treatments. Accessed 19 April 2020. https://www.nice.org.uk/guidance/ng161/chapter/6-Prioritising-systemic-anticancer-treatments

11 American Cancer Society. Survey: COVID-19 Affecting Patients' Access to Cancer Care. Accessed 19 April 2020. https://www.fightcancer.org/releases/survey-covid-19-affecting-patients'-access-cancer-care-5

12 American College of Surgeons. COVID-19 Guidelines for Triage of Breast Cancer Patients. Accessed 19 April 2020. https://www.facs.org/covid-19/clinical-guidance/elective-case/breast-cancer

13 American Society for Radiation Oncology. COVID-19 Coding Guidance. Accessed 19 April 2020. https://www.astro.org/Daily-Practice/Coding/Coding-Guidance/Coding-Updates/COVID-19-Coding-Guidance

14 Sud A, Jones M, Broggio J, *et al.* Collateral damage: the impact on outcomes from cancer

For peer review only

For peer review only - http://bmjopen.bmj.com/site/about/guidelines.xhtml

surgery of the COVID-19 pandemic. *Ann Oncol* 2020.

15 Sud A, Jones ME, Broggio J, *et al.* Quantifying and mitigating the impact of the COVID-19 pandemic on outcomes in colorectal cancer. 2020.

16 Lee LYW, Cazier JB, Starkey T, *et al.* COVID-19 mortality in patients with cancer on chemotherapy or other anticancer treatments: a prospective cohort study. *Lancet* 2020.

17 Public Health England. Guidance on shielding and protecting people defined on medical grounds as extremely vulnerable from COVID-19. Accessed 19 April 2020. https://www.gov.uk/government/publications/guidance-on-shielding-and-protecting-extremely-vulnerable-persons-from-covid-19/guidance-on-shielding-and-protecting-extremely-vulnerable-persons-from-covid-19

18 Public Health England. Guidance on social distancing for everyone in the UK. Accessed 19 April 2020. https://www.gov.uk/government/publications/covid-19-guidance-on-social-distancing-and-for-vulnerable-people/guidance-on-social-distancing-for-everyone-in-the-uk-and-protecting-older-people-and-vulnerable-adults

19 Hanna TP, Evans GA, Booth CM. Cancer, COVID-19 and the precautionary principle: prioritizing treatment during a global pandemic. *Nat Rev Clin Oncol* 2020;:1–3.

20 Renzi C, Kaushal A, Emery J, *et al.* Comorbid chronic diseases and cancer diagnosis: disease-specific effects and underlying mechanisms. *Nat Rev Clin Oncol* Published Online First: 2019. doi:10.1038/s41571-019-0249-6

21 Health Data Research UK. DATA-CAN - The Health Data Research Hub for Cancer. Accessed 20 April 2020. https://www.hdruk.ac.uk/infrastructure/the-hubs/data-can/

22 George J, Mathur R, Shah AD, *et al.* Ethnicity and the first diagnosis of a wide range of cardiovascular diseases: Associations in a linked electronic health record cohort of 1 million patients. *PLoS One* 2017;**12**:1–17. doi:10.1371/journal.pone.0178945

23 Bhaskaran K, Forbes HJ, Douglas I, *et al.* Representativeness and optimal use of body mass index (BMI) in the UK Clinical Practice Research Datalink (CPRD). *BMJ Open* 2013;**3**:e003389.

24 Mathur R, Bhaskaran K, Chaturvedi N, *et al.* Completeness and usability of ethnicity data in UK-based primary care and hospital databases. *J Public Health (Bangkok)* 2014;**36**:684–92.

25 Herrett E, Thomas SL, Schoonen WM, *et al.* Validation and validity of diagnoses in the General Practice Research Database: a systematic review. *Br J Clin Pharmacol* 2010;**69**:4–14.

26 Denaxas S, Gonzalez-Izquierdo A, Direk K, *et al.* UK phenomics platform for developing and validating electronic health record phenotypes: CALIBER. *J Am Med Informatics Assoc* 2019.

27 Denaxas SC, George J, Herrett E, *et al.* Data resource profile: cardiovascular disease research using linked bespoke studies and electronic health records (CALIBER). *Int J Epidemiol* 2012;**41**:1625–38.

For peer review only - http://bmjopen.bmj.com/site/about/guidelines.xhtml

28 Kuan V, Denaxas S, Gonzalez-Izquierdo A, *et al.* A chronological map of 308 physical and mental health conditions from 4 million individuals in the English National Health Service. *Lancet Digit Heal* 2019;**1**:e63–77. doi:10.1016/S2589-7500(19)30012-3

29 Shah AD, Langenberg C, Rapsomaniki E, *et al.* Type 2 diabetes and incidence of cardiovascular diseases: A cohort study in 1·9 million people. *Lancet Diabetes Endocrinol* 2015;**3**:105–13. doi:10.1016/S2213-8587(14)70219-0

30 Rapsomaniki E, Timmis A, George J, *et al.* Blood pressure and incidence of twelve cardiovascular diseases: Lifetime risks, healthy life-years lost, and age-specific associations in 1·25 million people. *Lancet* 2014;**383**:1899–911. doi:10.1016/S0140-6736(14)60685-1

31 Pikoula M, Quint JK, Nissen F, *et al.* Identifying clinically important COPD sub-types using data-driven approaches in primary care population based electronic health records. *BMC Med Inform Decis Mak* 2019;**19**:86.

32 Pujades-Rodriguez M, Duyx B, Thomas SL, *et al.* Rheumatoid arthritis and incidence of twelve initial presentations of cardiovascular disease: a population record-linkage cohort study in England. *PLoS One* 2016;**11**.

33 Dai M, Liu D, Liu M, *et al.* Patients with cancer appear more vulnerable to SARS-COV-2: a multi-center study during the COVID-19 outbreak. *Cancer Discov* Published Online First: 2020. doi:10.1158/2159-8290.CD-20-0422

34 Office for National Statistics. Coronavirus (COVID-19) Infection Survey. https://www.ons.gov.uk/peoplepopulationandcommunity/healthandsocialcare/conditionsanddiseases/datasets/coronaviruscovid19infectionsurveydata. Accessed 3 September 2020.

35 GOV UK. Sero-surveillance of COVID-19. https://www.gov.uk/government/publications/national-covid-19-surveillance-reports/sero-surveillance-of-covid-19. Accessed 3 September 2020.

36 Valenti L, Bergna A, Pelusi S, *et al.* SARS-CoV-2 seroprevalence trends in healthy blood donors during the COVID-19 Milan outbreak. *medRxiv* 2020.

37 Salje H, Kiem CT, Lefrancq N, *et al.* Estimating the burden of SARS-CoV-2 in France. *Science (80-)* 2020.

38 Understanding Society COVID-19 survey. Accessed 17 June 2020. https://www.understandingsociety.ac.uk/sites/default/files/downloads/general/ukhls_briefingnote_covid_health_final.pdf.

39 Office for National Statistics. Estimates of the population for the UK, England and Wales, Scotland and Northern Ireland. Accessed 23 April 2020. https://www.ons.gov.uk/peoplepopulationandcommunity/populationandmigration/populationestimates/datasets/populationestimatesforukenglandandwalesscotlandandnorthernireland

40 NHS England. NHS warning to seek help for cancer symptoms, as half of public report concerns with getting checked. https://tinyurl.com/y6owtj4u.

41 Margulis A V, Fortuny J, Kaye JA, *et al.* Validation of cancer cases using primary care,

For peer review only

cancer registry, and hospitalization data in the United Kingdom. *Epidemiology* 2018;**29**:308.

42 Banerjee A, Chen S, Pasea L, *et al.* Excess deaths in people with cardiovascular diseases during the COVID-19 pandemic. *medRxiv* Published Online First: 2020. doi:10.1101/2020.06.10.20127175

43 Kroenke CH, Kubzansky LD, Schernhammer ES, *et al.* Social networks, social support, and survival after breast cancer diagnosis. *J Clin Oncol* 2006;**24**:1105–11.

44 Elovainio M, Hakulinen C, Pulkki-Råback L, *et al.* Contribution of risk factors to excess mortality in isolated and lonely individuals: an analysis of data from the UK Biobank cohort study. *Lancet Public Heal* 2017;**2**:e260--e266.

45 Maruthappu M, Watkins J, Noor AM, *et al.* Economic downturns, universal health coverage, and cancer mortality in high-income and middle-income countries, 1990--2010: a longitudinal analysis. *Lancet* 2016;**388**:684–95.

46 Longer-Run Economic Consequences of Pandemics. Accessed 1 May 2020. https://www.frbsf.org/economic-research/files/wp2020-09.pdf

For peer review only

For peer review only - http://bmjopen.bmj.com/site/about/guidelines.xhtml

Figure legends:

Figure 1. Weekly hospital data (January 2019 to June 2020) on changes in urgent referrals and chemotherapy clinic attendance from eight hospitals in the UK mapped to phases of the pandemic. Weekly changes from January 2020 to June 2020 were mapped to phases of the pandemic. Weekly values were plotted as percentage increase or decrease relative to the 2019 average. The data for Northern Ireland includes five Health and Social Care Trusts (HSCs) that cover all health service provision in Northern Ireland: Belfast HSC, Northern HSC, South Eastern HSC, Southern HSC and Western HSC. Vertical dotted lines indicate the Christmas Bank Holiday.

Figure 2. Office for National Statistics data on weekly registrations of deaths in the England and Wales from 3 January 2020 to 15 May 2020. (A) Upper panel indicates the number of weekly deaths. (B) Lower panel indicates weekly changes in relative risk estimates calculated by comparing the current weekly deaths to 5-year weekly averages. Dates indicate week ending on a particular date.

Figure 3. Estimated total (direct and indirect) excess deaths by cancer site over a 1-year period (A) 1-year mortality for incident and prevalent cancers. The whiskers are 95% confidence intervals. (B) Total excess deaths were scaled up to the population of England aged 30+ consisting of 35 million individuals using England mortality estimates for both incident and prevalent cancers combined. We estimated direct excess deaths at a 10% infection rate. We estimated total (direct and indirect) excess deaths for 40% (10% infected, 30% affected) and 80% (10% infected, 70% affected) of the population.

Figure 4. Total (direct and indirect) excess deaths for both incident and prevalent cancers by cancer site and number of comorbidities over a 1-year period. Stacked bar chart indicates the proportion of individuals with 0, 1, 2 and 3+ comorbidities by cancer site. We estimated total excess deaths for 40% (10% infected, 30% affected) of the population. Total excess deaths were scaled up to the population of England aged 30+ consisting of 35 million individuals using England mortality estimates for both incident and prevalent cancers combined.

Supplementary figure legends:

Figure S1. Age-adjusted incidence rates for 24 primary cancers from England and the UK (International Agency for Research on Cancer [IARC]) For England data, cervix refers to both carcinoma in situ of cervix and primary malignancy of cervix. For IARC data, only cervix uteri are included. CRUK: Cancer Research UK.

For peer review only - http://bmjopen.bmj.com/site/about/guidelines.xhtml

Figure S2. Total excess deaths for incident cancers over a 1-year period scaled up to the population of England aged 30+ consisting of 35 million individuals using England mortality estimates. We estimated direct excess deaths at a 10% infection rate. We estimated total (direct and indirect) excess deaths for 40% (10% infected, 30% affected) and 80% (10% infected, 70% affected) of the population.

Figure S3. Total excess deaths for prevalent cancers over a 1-year period scaled up to the population of England aged 30+ consisting of 35 million individuals using England mortality estimates. We estimated direct excess deaths at a 10% infection rate. We estimated total (direct and indirect) excess deaths for 40% (10% infected, 30% affected) and 80% (10% infected, 70% affected) of the population.

Figure S4. Proportion of patients with any of the 15 comorbidity clusters by cancer site for incident cancers (N=199,057) in a population of 3,862,012 adults in England. Age indicates mean age at diagnosis.

Figure S5. Proportion of patients with any of the 15 comorbidity clusters by cancer site for prevalent cancers (N=117,978) in a population of 3,862,012 adults in England. Age indicates mean age at diagnosis.

Figure S6. Forest plot of background (pre-COVID-19) 1-year cancer mortality for incident cases according to cancer site and number of underlying comorbidities in England. The whiskers are 95% confidence intervals.

Figure S7. Forest plot of background (pre-COVID-19) 1-year cancer mortality for prevalent cases according to cancer site and number of underlying comorbidities in England. The whiskers are 95% confidence intervals.

Figure S8. Total (direct and indirect) excess deaths for incident cancers by cancer site and number of comorbidities over a 1-year period. Stacked bar chart indicates the proportion of individuals with 0, 1, 2 and 3+ comorbidities by cancer site. We estimated total excess deaths for 40% (10% infected, 30% affected) of the population. Total excess deaths were scaled up to the population of England aged 30+ consisting of 35 million individuals using England mortality estimates for incident cancers.

Figure S9. Total (direct and indirect) excess deaths for prevalent cancers by cancer site and number of comorbidities over a 1-year period. Stacked bar chart indicates the proportion of individuals with 0, 1, 2 and 3+ comorbidities by cancer site. We estimated total excess deaths for 40% (10% infected,

For peer review only - http://bmjopen.bmj.com/site/about/guidelines.xhtml

30% affected) of the population. Total excess deaths were scaled up to the population of England aged 30+ consisting of 35 million individuals using England mortality estimates for prevalent cancers.

For peer review only

For peer review only - http://bmjopen.bmj.com/site/about/guidelines.xhtml

Supplementary Methods

Description on 15 comorbidity clusters relevant to COVID-19

The PHE list included chronic respiratory disease, chronic heart disease, immunocompromised individuals, HIV, use of corticosteroids, obesity, diabetes, chronic kidney disease, chronic liver disease, chronic neurological disorders and splenic disorders. We have performed analyses for all the above conditions and have additionally considered hypertension, Crohn's disease, cystic fibrosis and rheumatoid arthritis. Given that condition clusters such as **(i)** chronic heart disease would involve a range of conditions, we have derived composite variables to include 15 conditions considered as cardiovascular disease (CVD) that included acute myocardial infarction, unstable angina, chronic stable angina, heart failure, cardiac arrest or sudden coronary death, transient ischemic attack, intracerebral haemorrhage, subarachnoid haemorrhage, ischemic stroke, abdominal aortic aneurysm, peripheral arterial disease, atrial fibrillation, congenital heart disease, hypertrophic and dilated cardiomyopathy and valve disease (multiple, mitral and aortic)[29]. We also considered **(ii)** Hypertension, defined as ≥140 mmHg systolic blood pressure (or ≥150 mmHg for people aged ≥60 years without diabetes and chronic kidney disease) and/or ≥90 mmHg diastolic blood pressure[30], **(iii)** type 2 diabetes, **(iv)** obesity, defined as a body mass index of ≥40kg/m^2, **(v)** chronic kidney disease (CKD), **(vi)** chronic obstructive pulmonary disease (COPD)[31], **(vii)** patients on immunosuppressive drugs (not cancer chemotherapy), **(viii)** patients with HIV or corticosteroid prescription, **(ix)** chronic neurological disorders, defined as a composite of Parkinson's disease, motor neuron disease, learning disability and cerebral palsy, **(x)** multiple sclerosis separately, **(xi)** splenic disorders, defined as a composite of splenomegaly, splenectomy and hyposplenism, **(xii)** chronic liver diseases, defined as a composite of chronic viral hepatitis B or C, primary biliary cholangitis, liver fibrosis, liver cirrhosis and non-alcoholic fatty liver disease, **(xiii)** Crohn's disease, **(xiv)** cystic fibrosis and **(xv)** rheumatoid arthritis[32].

For peer review only

For peer review only - http://bmjopen.bmj.com/site/about/guidelines.xhtml

Figure 1. Weekly hospital data (January 2019 to June 2020) on changes in urgent referrals and chemotherapy clinic attendance from eight hospitals in the UK mapped to phases of the pandemic. Weekly changes from January 2020 to June 2020 were mapped to phases of the pandemic. Weekly values were plotted as percentage increase or decrease relative to the 2019 average. The data for Northern Ireland includes five Health and Social Care Trusts (HSCs) that cover all health service provision in Northern Ireland: Belfast HSC, Northern HSC, South Eastern HSC, Southern HSC and Western HSC. Vertical dotted lines indicate the Christmas Bank Holiday.

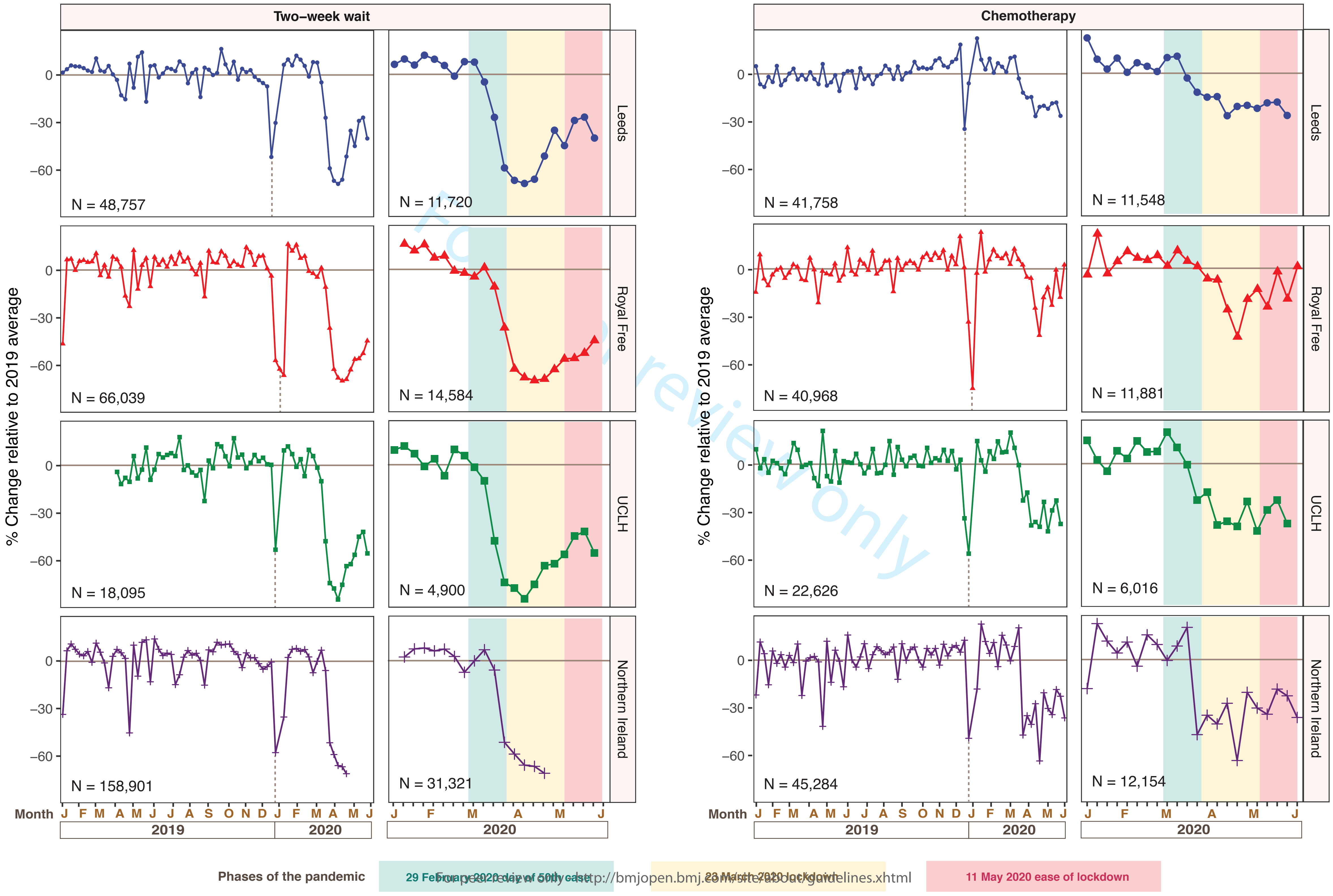

For peer review only

Figure 2. Office for National Statistics data on weekly registrations of deaths in the England and Wales from 3 January 2020 to 15 May 2020. (A) Upper panel indicates the number of weekly deaths. (B) Lower panel indicates weekly changes in relative risk estimates calculated by comparing the current weekly deaths to 5-year weekly averages. Dates indicate week ending on a particular date.

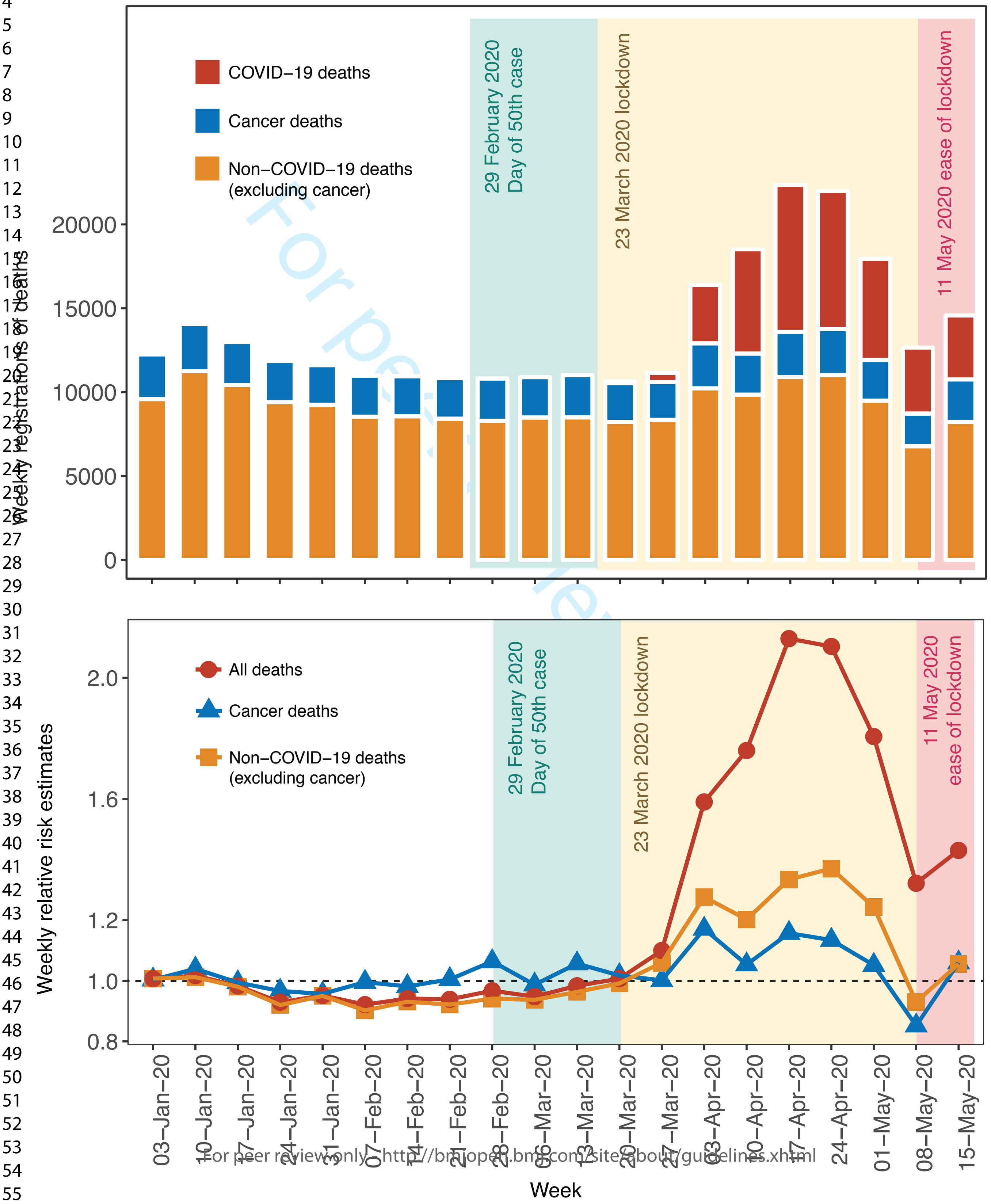

Figure 3. Estimated total (direct and indirect) excess deaths by cancer site over a 1-year period (A) 1-year mortality for incident and prevalent cancers. The whiskers are 95% confidence intervals. (B) Total excess deaths were scaled up to the population of England aged 30+ consisting of 35 million individuals using England mortality estimates for both incident and prevalent cancers combined. We estimated direct excess deaths at a 10% infection rate. We estimated total (direct and indirect) excess deaths for 40% (10% infected, 30% affected) and 80% (10% infected, 70% affected) of the population.

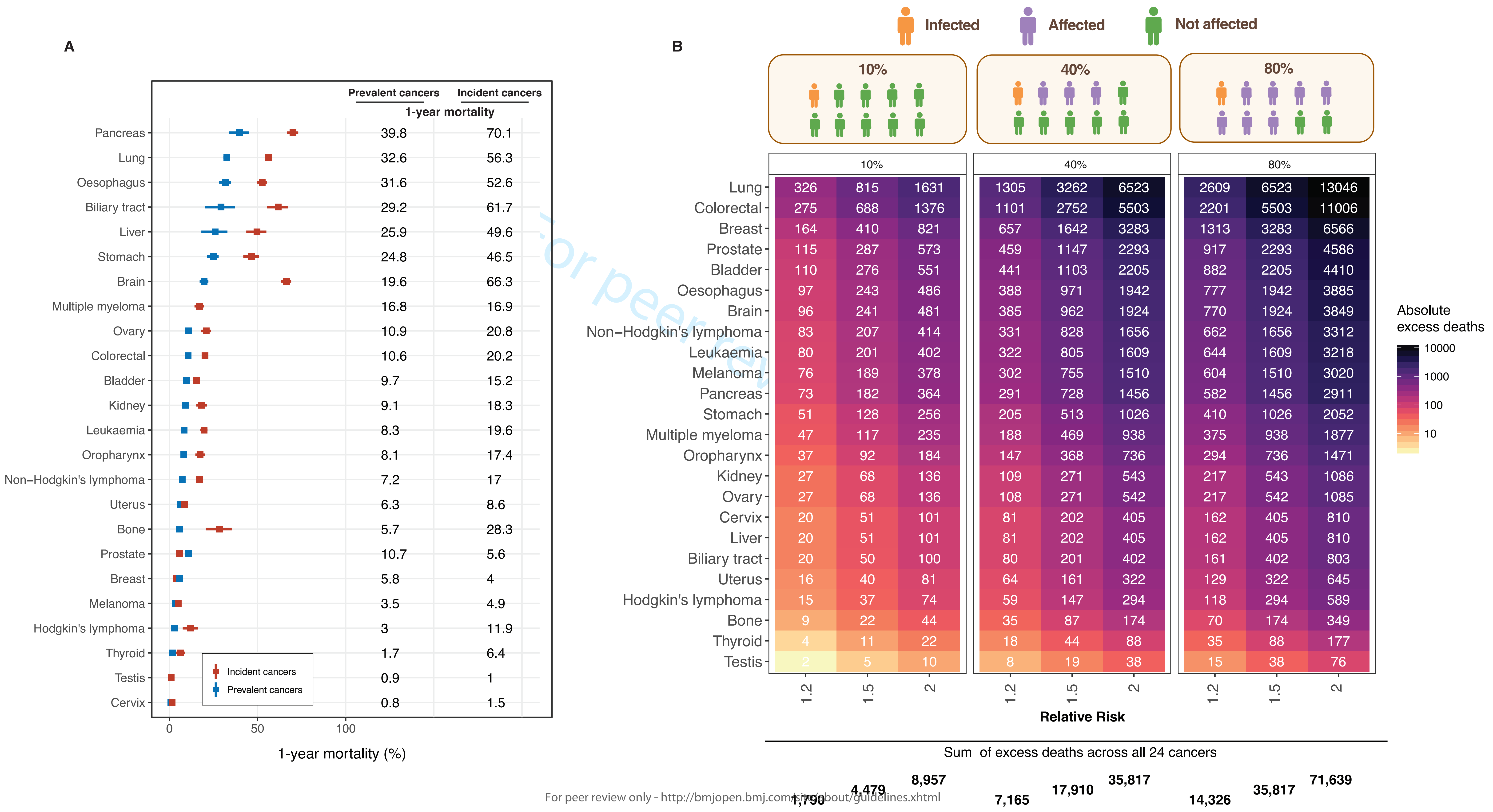

Figure 4. Total (direct and indirect) excess deaths for both incident and prevalent cancers by cancer site and number of comorbidities over a 1-year period. Stacked bar chart indicates the proportion of individuals with 0, 1, 2 and 3+ comorbidities by cancer site. We estimated total excess deaths for 40% (10% infected, 30% affected) of the population. Total excess deaths were scaled up to the population of England aged 30+ consisting of 35 million individuals using England mortality estimates for both incident and prevalent cancers combined.

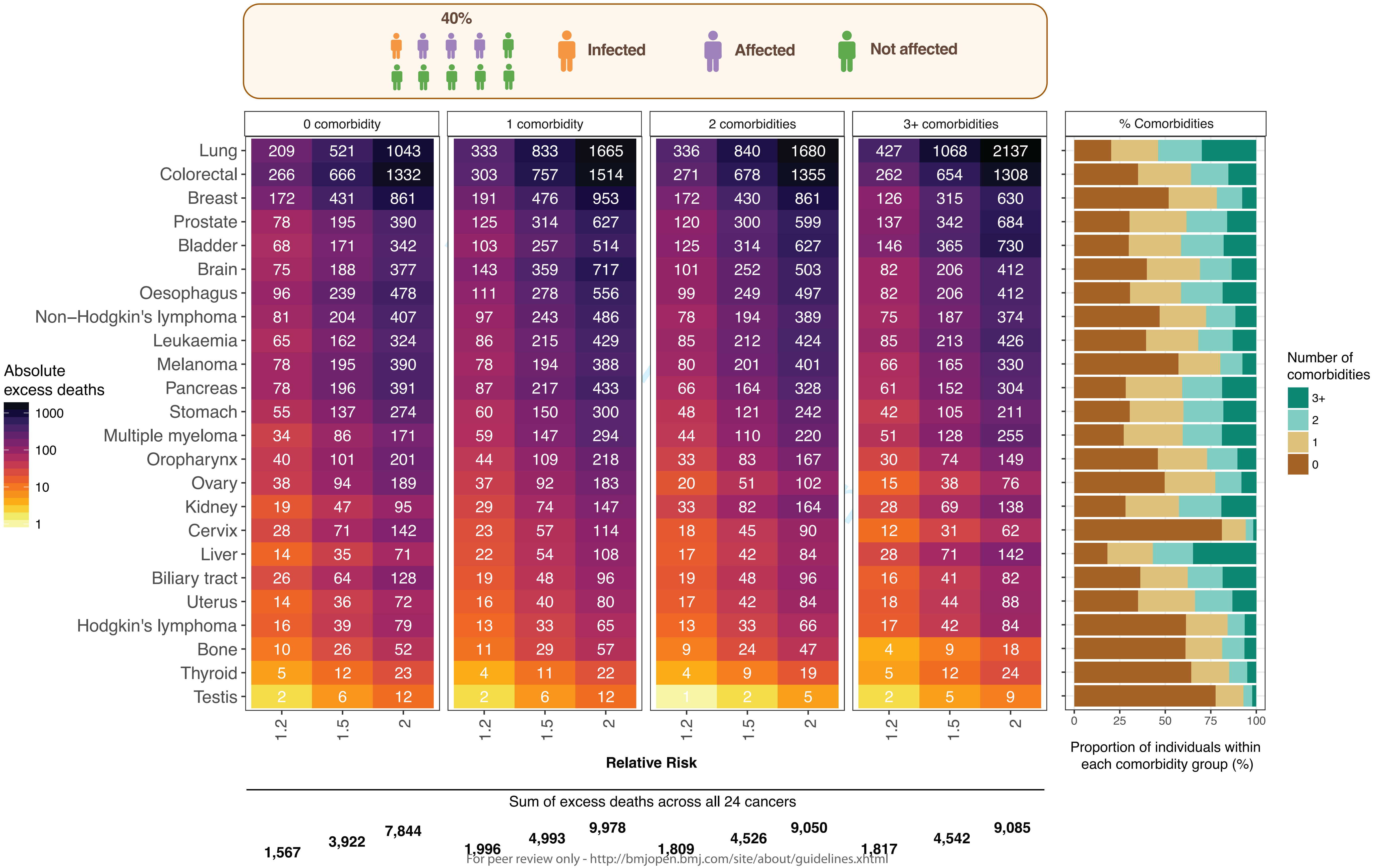

Figure S1. Age-adjusted incidence rates for 24 primary cancers from England and the UK (International Agency for Research on Cancer [IARC]) For England data, cervix refers to both carcinoma in situ of cervix and primary malignancy of cervix. For IARC data, only cervix uteri are included. CRUK: Cancer Research UK.

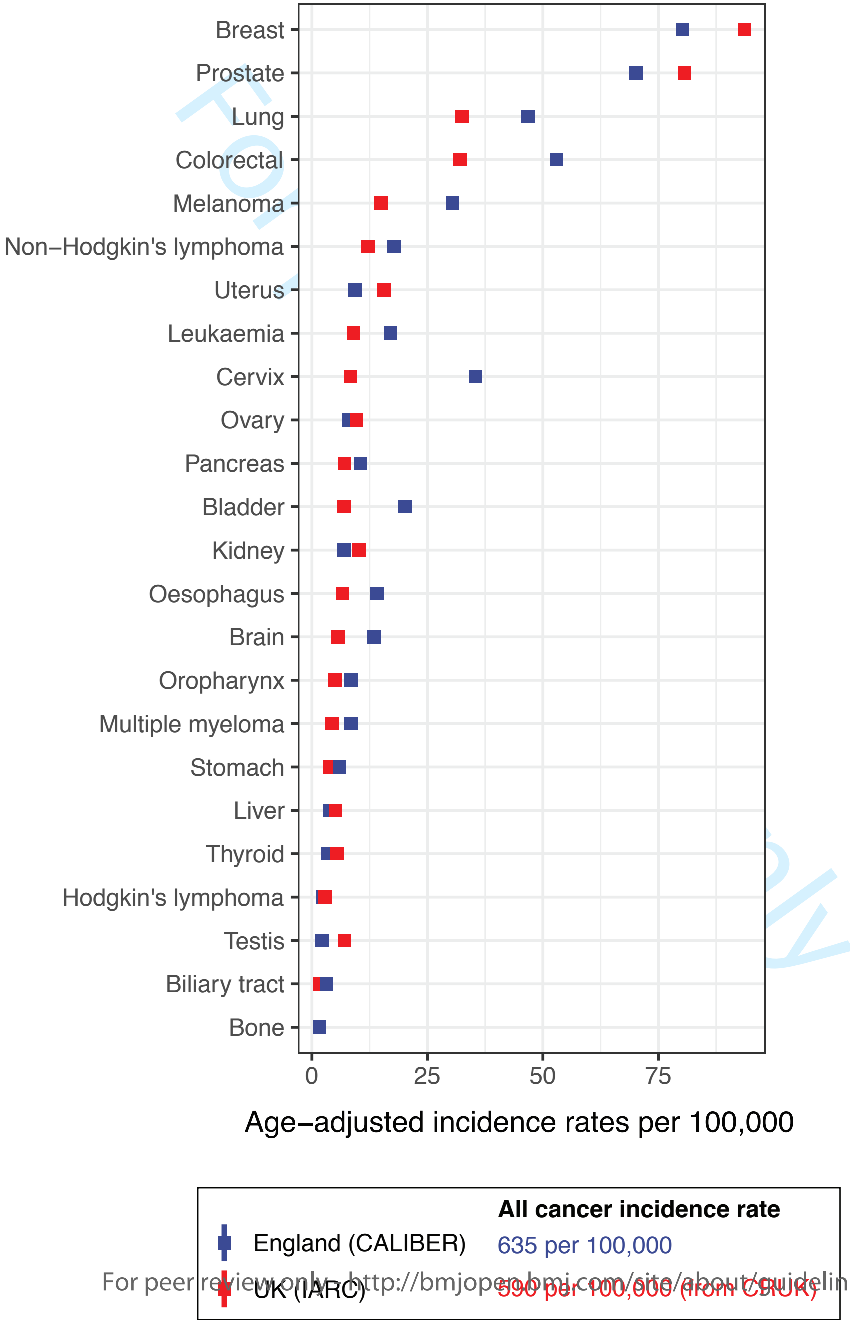

Figure S2. Total excess deaths for incident cancers over a 1-year period scaled up to the population of England aged 30+ consisting of 35 million individuals using England mortality estimates. We estimated direct excess deaths at a 10% infection rate. We estimated total (direct and indirect) excess deaths for 40% (10% infected, 30% affected) and 80% (10% infected, 70% affected) of the population.

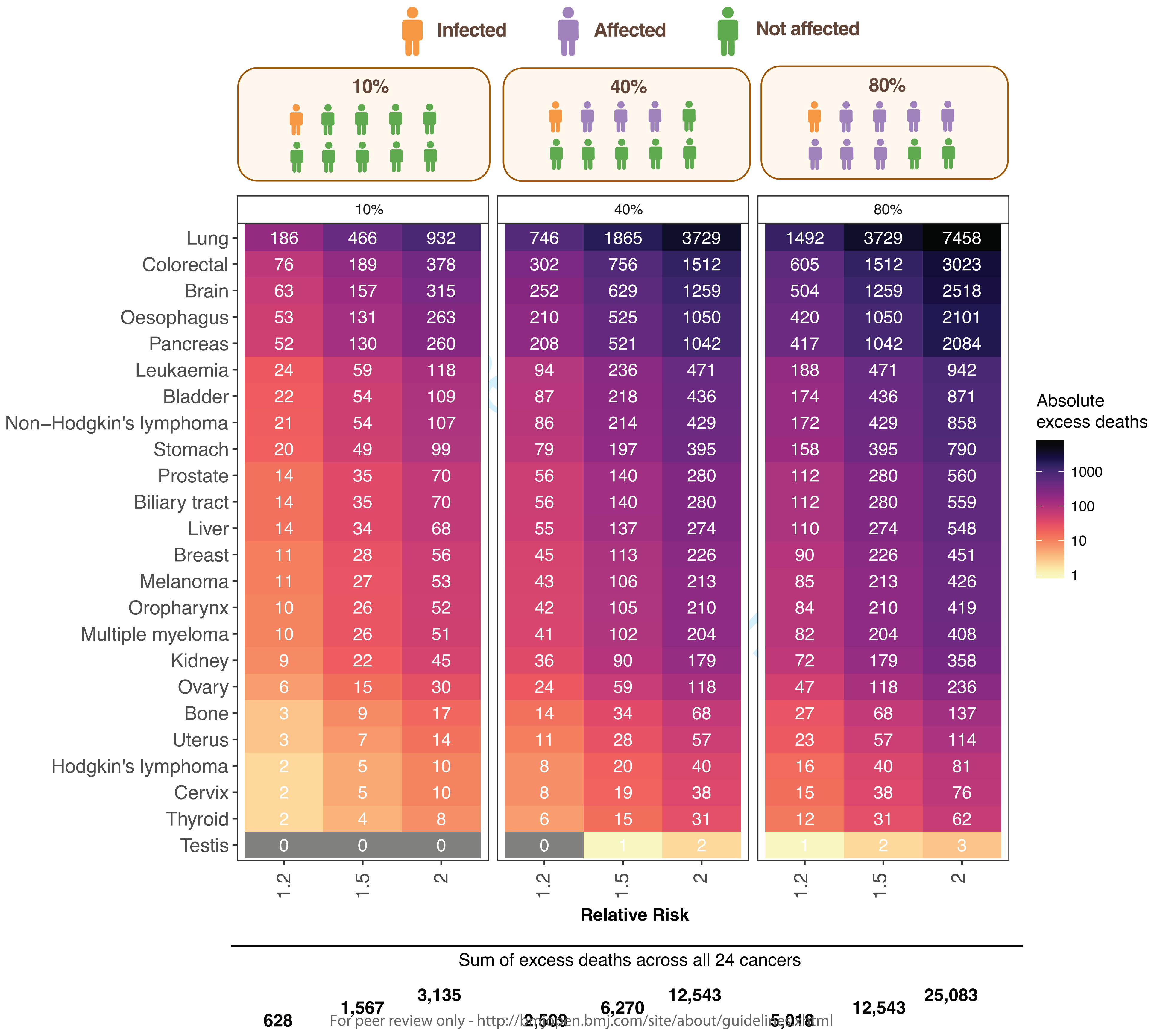

Figure S3. Total excess deaths for prevalent cancers over a 1-year period scaled up to the population of England aged 30+ consisting of 35 million individuals using England mortality estimates. We estimated direct excess deaths at a 10% infection rate. We estimated total (direct and indirect) excess deaths for 40% (10% infected, 30% affected) and 80% (10% infected, 70% affected) of the population.

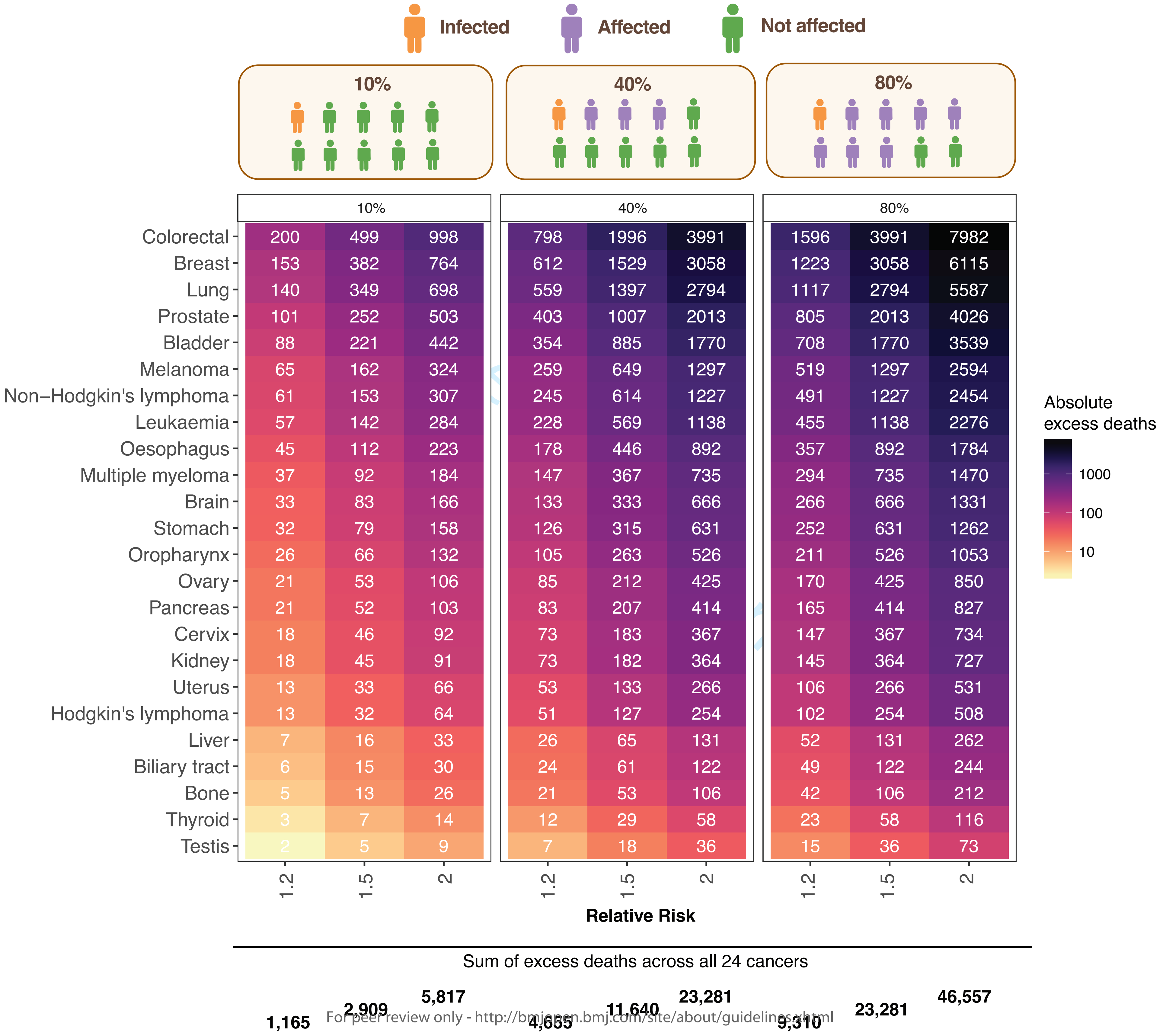

Figure S4. Proportion of patients with any of the 15 comorbidity clusters by cancer site for incident cancers (N=199,057) in a population of 3,862,012 adults in England. Age indicates mean age at diagnosis.

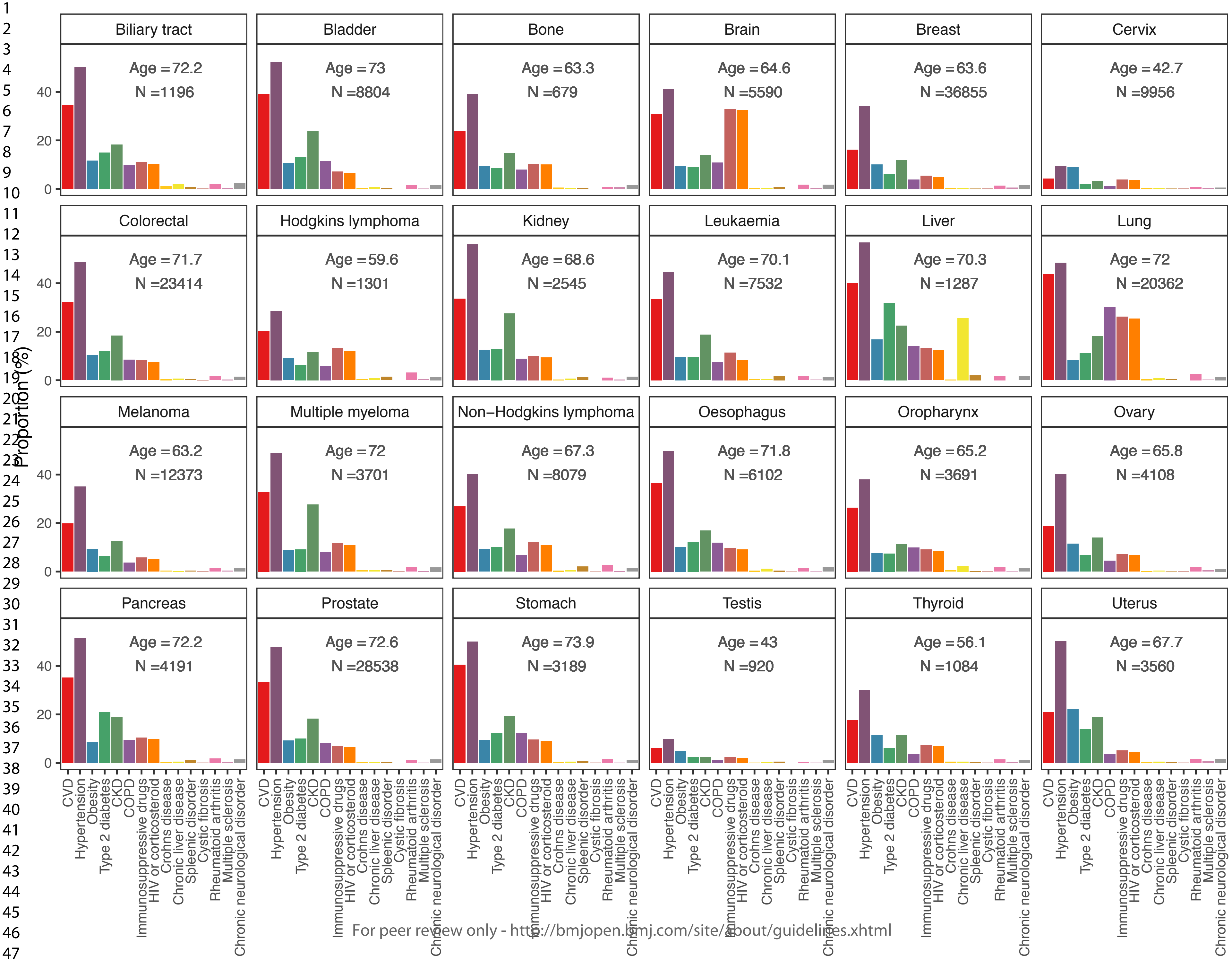

Figure S5. Proportion of patients with any of the 15 comorbidity clusters by cancer site for prevalent cancers (N=117,978) in a population of 3,862,012 adults in England. Age indicates mean age at diagnosis.

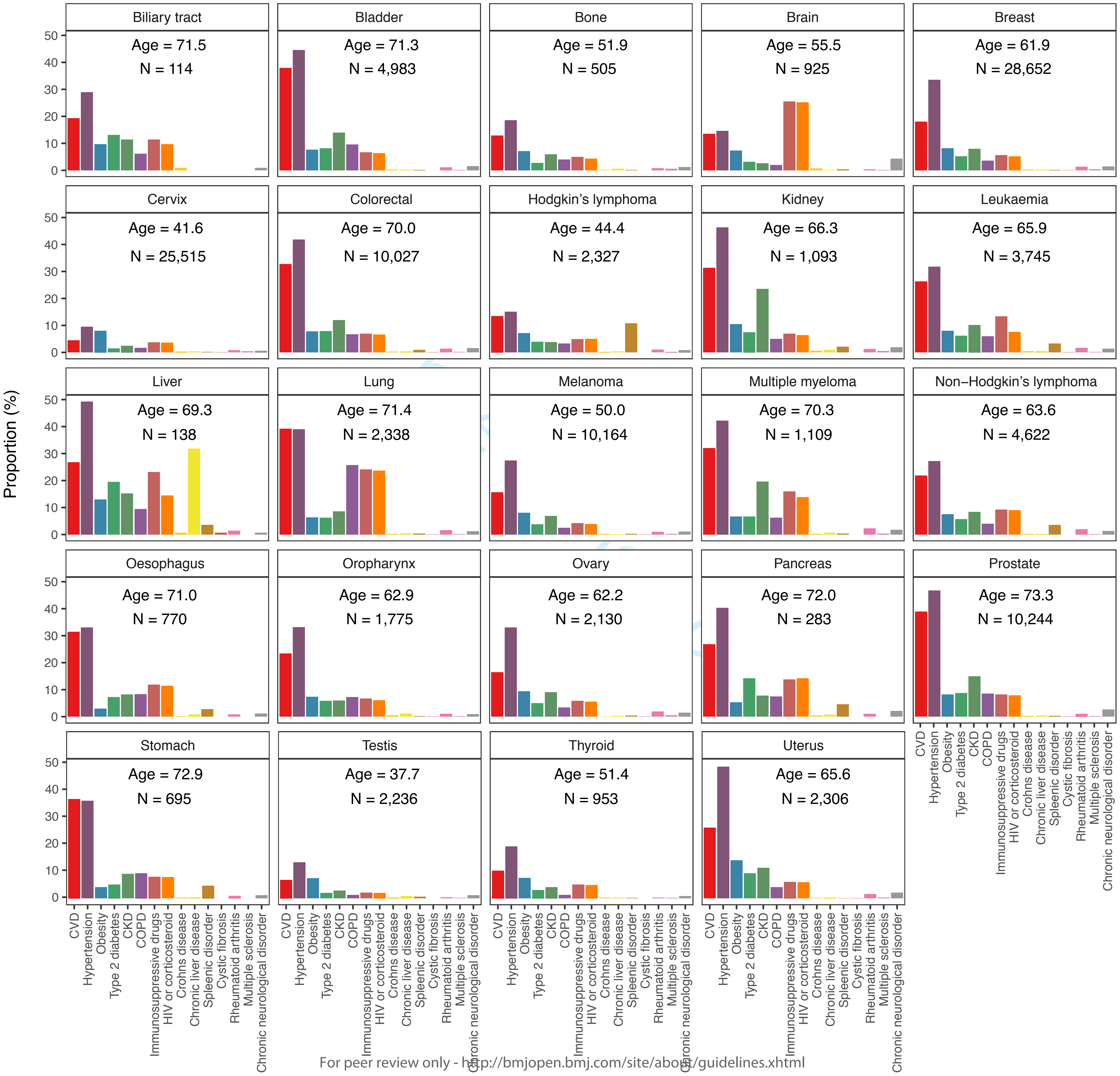

Figure S6. Forest plot of background (pre-COVID-19) 1-year cancer mortality for incident cases according to cancer site and number of underlying comorbidities in England. The whiskers are 95% confidence intervals.

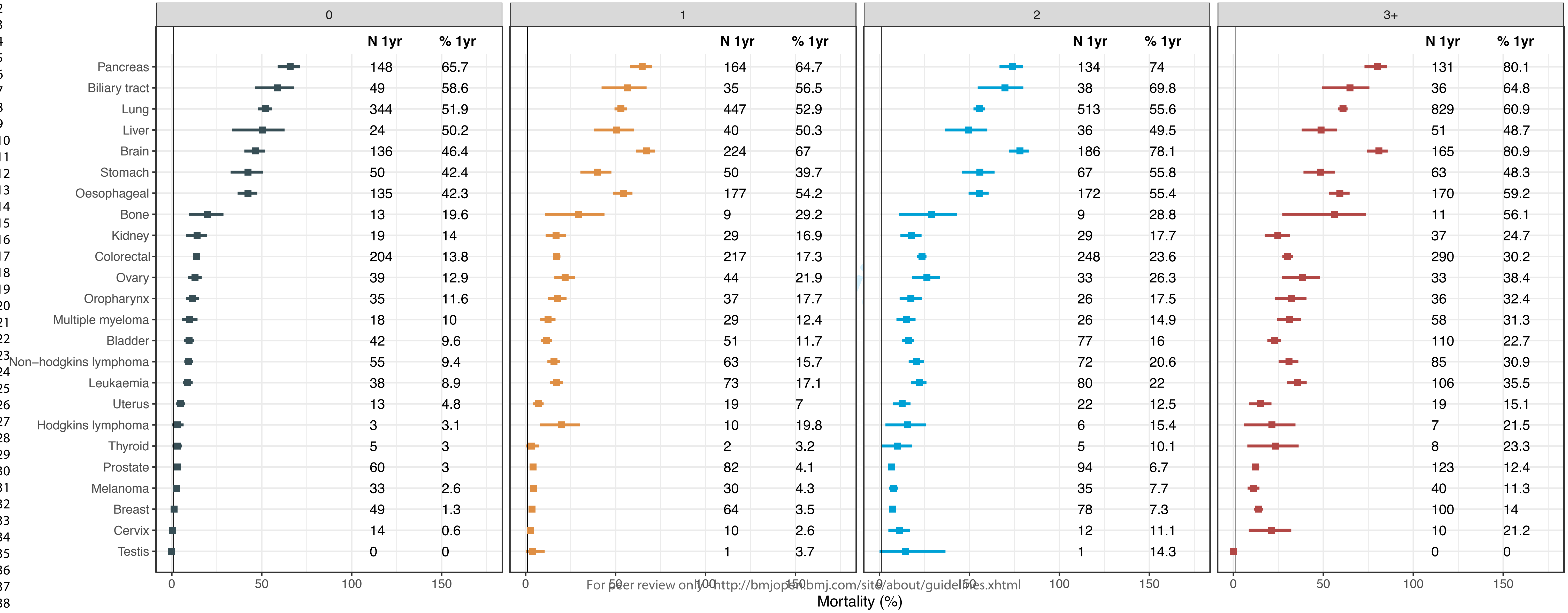

Figure S7. Forest plot of background (pre-COVID-19) 1-year cancer mortality for prevalent cases according to cancer site and number of underlying comorbidities in England. The whiskers are 95% confidence intervals.

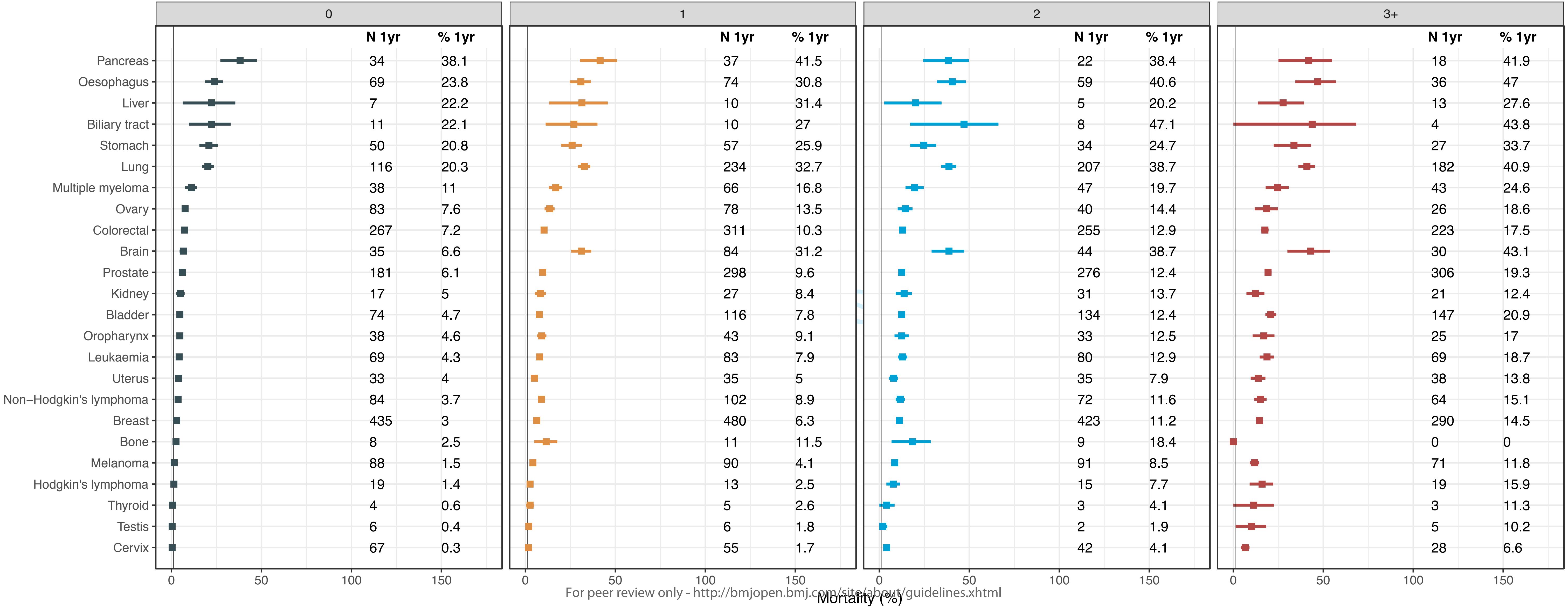

Figure S8. Total (direct and indirect) excess deaths for incident cancers by cancer site and number of comorbidities over a 1-year period. Stacked bar chart indicates the proportion of individuals with 0, 1, 2 and 3+ comorbidities by cancer site. We estimated total excess deaths for 40% (10% infected, 30% affected) of the population. Total excess deaths were scaled up to the population of England aged 30+ consisting of 35 million individuals using England mortality estimates for incident cancers.

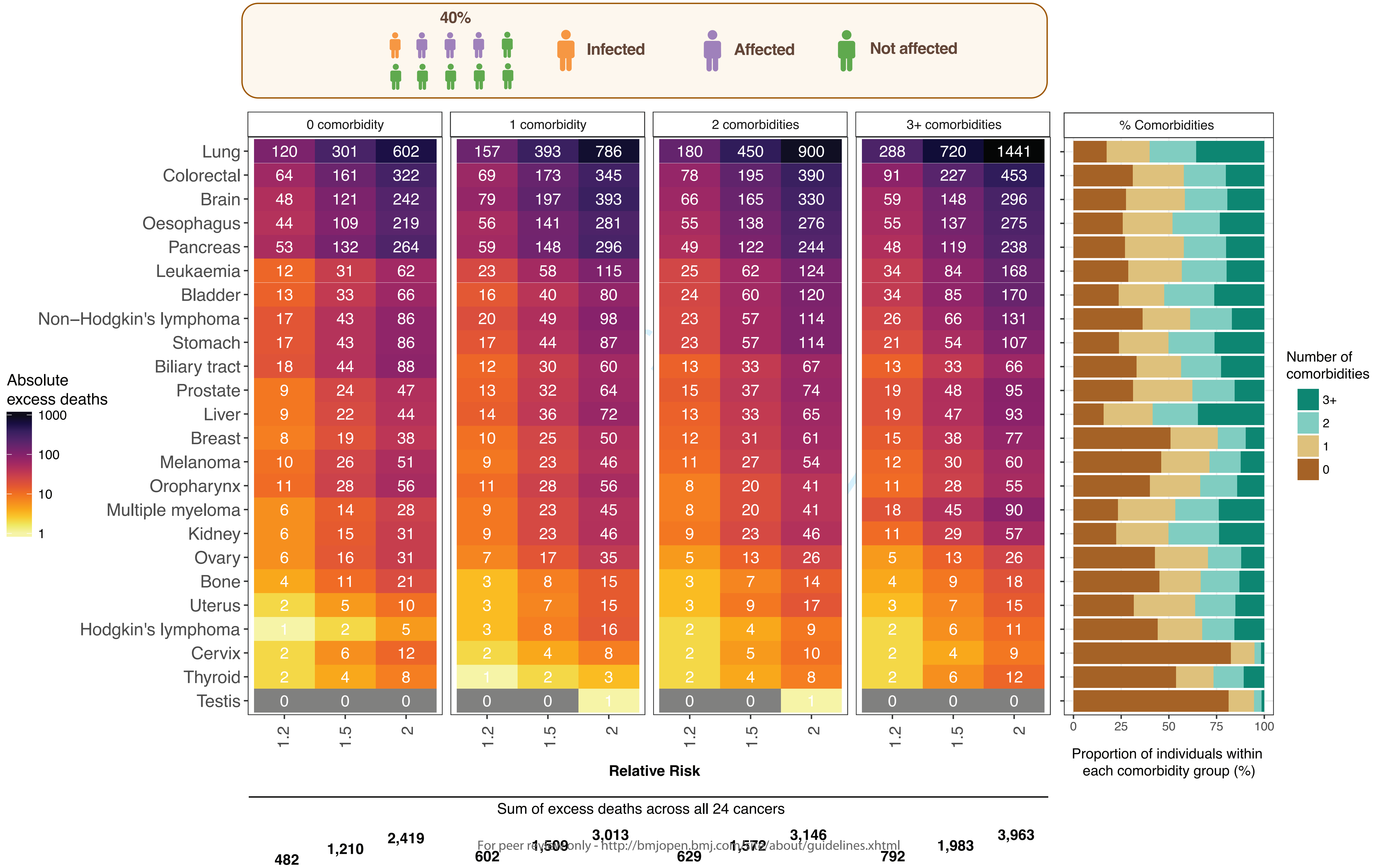

Figure S9. Total (direct and indirect) excess deaths for prevalent cancers by cancer site and number of comorbidities over a 1-year period. Stacked bar chart indicates the proportion of individuals with 0, 1, 2 and 3+ comorbidities by cancer site. We estimated total excess deaths for 40% (10% infected, 30% affected) of the population. Total excess deaths were scaled up to the population of England aged 30+ consisting of 35 million individuals using England mortality estimates for prevalent cancers.

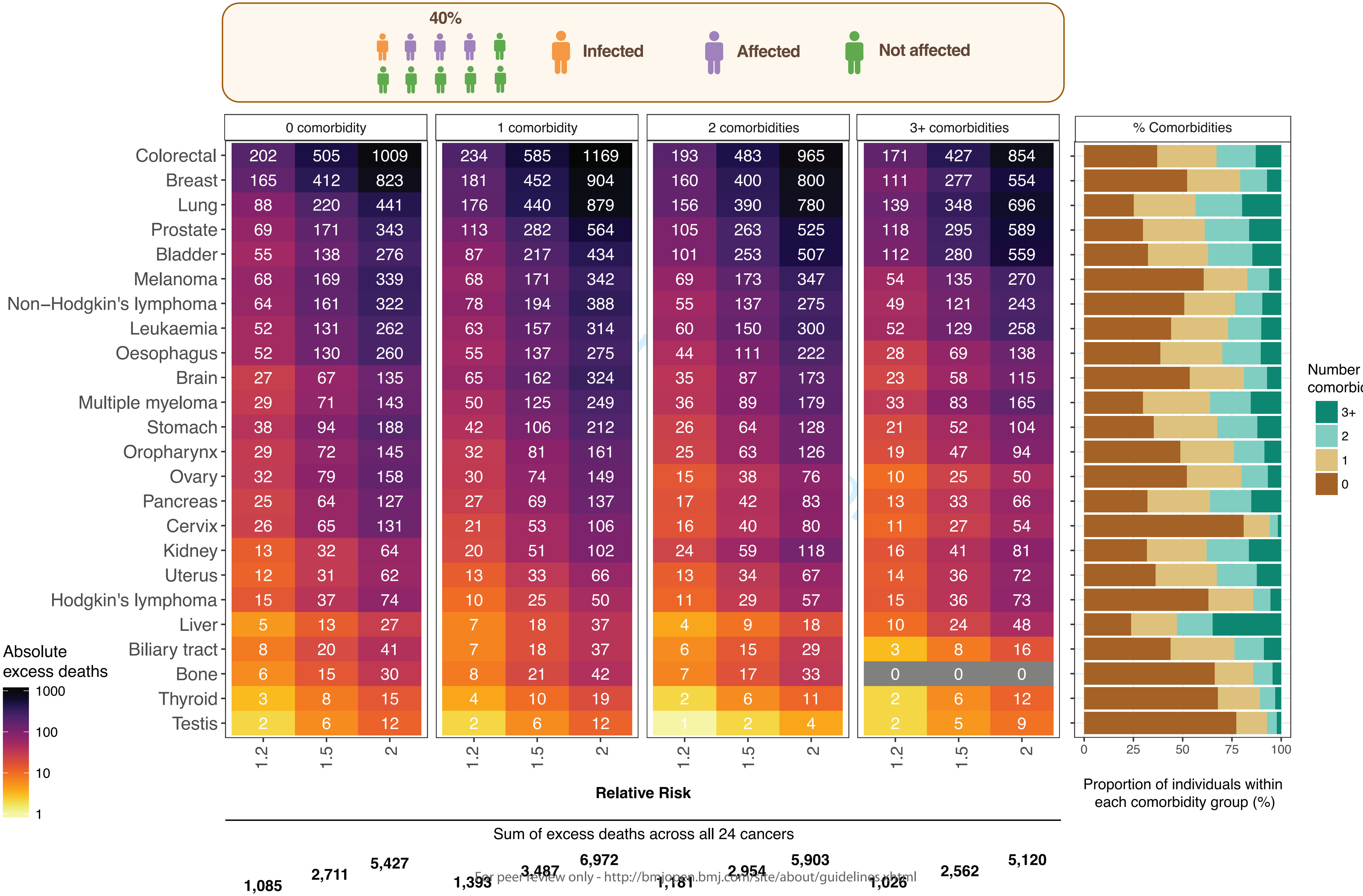

STROBE Statement—Checklist of items that should be included in reports of ***cohort studies***

	Item No	Recommendation
**Title and abstract**	1	(*a*) Indicate the study's design with a commonly used term in the title or the abstract **(p. 2)**
		(*b*) Provide in the abstract an informative and balanced summary of what was done and what was found **(p. 3)**
**Introduction**		
Background/rationale	2	Explain the scientific background and rationale for the investigation being reported **(pp. 4)**
Objectives	3	State specific objectives, including any prespecified hypotheses **(p. 4)**
**Methods**		
Study design	4	Present key elements of study design early in the paper **(pp. 5)**
Setting	5	Describe the setting, locations, and relevant dates, including periods of recruitment, exposure, follow-up, and data collection **(pp. 5)**
Participants	6	(*a*) Give the eligibility criteria, and the sources and methods of selection of participants. Describe methods of follow-up **(pp. 5)**
		(*b*) For matched studies, give matching criteria and number of exposed and unexposed **(-)**
Variables	7	Clearly define all outcomes, exposures, predictors, potential confounders, and effect modifiers. Give diagnostic criteria, if applicable **(pp. 5-7)**
Data sources/ measurement	8*	For each variable of interest, give sources of data and details of methods of assessment (measurement). Describe comparability of assessment methods if there is more than one group **(pp. 5)**
Bias	9	Describe any efforts to address potential sources of bias **(-)**
Study size	10	Explain how the study size was arrived at **(pp. 5)**
Quantitative variables	11	Explain how quantitative variables were handled in the analyses. If applicable, describe which groupings were chosen and why **(pp. 5-7)**
Statistical methods	12	(*a*) Describe all statistical methods, including those used to control for confounding **(pp. 5-7)**
		(*b*) Describe any methods used to examine subgroups and interactions **(-)**
		(*c*) Explain how missing data were addressed **(-)**
		(*d*) If applicable, explain how loss to follow-up was addressed **(-)**
		(*e*) Describe any sensitivity analyses **(-)**
**Results**		
Participants	13*	(a) Report numbers of individuals at each stage of study—eg numbers potentially eligible, examined for eligibility, confirmed eligible, included in the study, completing follow-up, and analysed **(pp. 8-9)**
		(b) Give reasons for non-participation at each stage **(-)**
		(c) Consider use of a flow diagram **(-)**
Descriptive data	14*	(a) Give characteristics of study participants (eg demographic, clinical, social) and information on exposures and potential confounders **(pp. 5-7)**
		(b) Indicate number of participants with missing data for each variable of interest **(-)**
		(c) Summarise follow-up time (eg, average and total amount) **(-)**
Outcome data	15*	Report numbers of outcome events or summary measures over time **(pp. 5-7)**
Main results	16	(*a*) Give unadjusted estimates and, if applicable, confounder-adjusted estimates and their precision (eg, 95% confidence interval). Make clear which confounders were adjusted for and why they were included **(pp. 8-9)**

For peer review only - http://bmjopen.bmj.com/site/about/guidelines.xhtml

		(*b*) Report category boundaries when continuous variables were categorized **(-)**
		(*c*) If relevant, consider translating estimates of relative risk into absolute risk for a meaningful time period **(-)**
Other analyses	17	Report other analyses done—eg analyses of subgroups and interactions, and sensitivity analyses **(-)**
**Discussion**		
Key results	18	Summarise key results with reference to study objectives **(pp. 10)**
Limitations	19	Discuss limitations of the study, taking into account sources of potential bias or imprecision. Discuss both direction and magnitude of any potential bias **(pp. 11)**
Interpretation	20	Give a cautious overall interpretation of results considering objectives, limitations, multiplicity of analyses, results from similar studies, and other relevant evidence **(pp. 11)**
Generalisability	21	Discuss the generalisability (external validity) of the study results **(p. 12)**
**Other information**		
Funding	22	Give the source of funding and the role of the funders for the present study and, if applicable, for the original study on which the present article is based **(pp. 14-15)**

For peer review only
